# Comprehensive Transcriptome Profiling of Peripheral Blood Mononuclear Cells from Patients with Sepsis

**DOI:** 10.7150/ijms.46910

**Published:** 2020-07-25

**Authors:** Tianzhou Wu, Xi Liang, Yongpo Jiang, Qi Chen, Huaping Zhang, Sheng Zhang, Chao Zhang, Yuhang Lv, Jiaojiao Xin, Jing Jiang, Dongyan Shi, Xin Chen, Jun Li, Yinghe Xu

**Affiliations:** 1 Precision Medicine Center, Taizhou Central Hospital, Taizhou University Medical School, Taizhou, China.; 2 Department of intensive care unit, Taizhou Hospital of Zhejiang Province, Wenzhou Medical University, Taizhou, China.; 3 Department of intensive care unit, Taizhou Enze Medical Center (Group) Enze Hospital, Taizhou, China.; 4 State Key Laboratory for Diagnosis and Treatment of Infectious Diseases, Collaborative National Clinical Research Center for Infectious Diseases, The First Affiliated Hospital, Zhejiang University School of Medicine.; 5 Institute of Pharmaceutical Biotechnology, Zhejiang University School of Medicine, Hangzhou, China.; 6 Department of intensive care unit, Taizhou Central Hospital, Taizhou University Medical School, Taizhou, China.

**Keywords:** sepsis, transcriptomics, peripheral blood mononuclear cells, biomarker

## Abstract

**Background:** Sepsis, as a clinical emergency, usually causes multiorgan dysfunction and can lead to high mortality. Establishment of specific and sensitive biomarkers for early diagnosis is critical to identify patients who would benefit from targeted therapy. In this study, we investigated this syndrome by analyzing the transcriptome of peripheral blood mononuclear cells (PBMCs) from patients with sepsis and identified sepsis-specific biomarkers.

**Methods:** In this study, a total of 87 patients with sepsis and 40 healthy controls from a prospective multicenter cohort were enrolled. Samples from 44 subjects (24 patients with sepsis and 20 healthy controls) were sequenced and the remaining patients were included in the validation group. Using high-throughput sequencing, a gene expression profile of PBMCs from patients with sepsis was generated to elucidate the pathophysiology of sepsis and identify sepsis-specific biomarkers.

**Results:** Principal component analysis (PCA) and unsupervised hierarchical cluster analysis showed that patients with sepsis separated from healthy controls. A total of 1639 differentially expressed genes (DEGs) were identified (|log2 fold change|>2, adjusted P value <0.05) between these two groups, with 1278 (78.0%) upregulated and 361 (22.0%) downregulated in patients with sepsis. Gene Ontology (GO) analysis of the upregulated DEGs identified 194 GO terms that were clustered into 27 groups, and analysis of the downregulated DEGs identified 20 GO terms that were clustered into 4 groups. Four unique genes were identified that could be predictive of patients with sepsis. External validation of the four genes using quantitative real-time polymerase chain reaction (qRT-PCR) was consistent with the results of mRNA sequencing, revealing their potential in sepsis diagnosis.

**Conclusions:** The transcriptome characteristics of PBMCs, which were significantly altered in sepsis patients, provide new insights into sepsis pathogenesis. The four identified gene expression changes differentiated patients with sepsis from healthy subjects, which could serve as a convenient tool contributing to sepsis diagnosis.

## Introduction

Sepsis is a potentially life-threatening condition in the intensive care unit (ICU) caused by the body's response to an infection, that can lead to tissue damage, organ failure, and death. The incidence of sepsis is very high (2.8 million deaths per year worldwide) in adults, with high mortality rates of 10-50%, depending on age and disease severity 1, 2. Despite the burden on patients and the healthcare service system, treatment remains mainly futile. Thus, precise diagnosis and targeted therapy for sepsis is crucial to reducing mortality, with every hour of delay increasing mortality risk 3.

Recent findings in transcriptome analysis have given us a greater understanding of the mechanisms behind diseases. Genome-wide gene expression profiling is extensively used to discover new potential biomarkers for the diagnosis or prediction of disease severity and identification of novel drug targets. However, tissue sampling has limited the accurate investigation of sepsis pathogenesis. Therefore, blood can be used as a surrogate and can be obtained with a minimally invasive procedure. Many studies have indicated that the messenger ribonucleic acid (mRNA) expression levels of specific genes in peripheral blood mononuclear cells (PBMCs) can serve as a signature of specific diseases, such as active pulmonary tuberculosis 4 and enterocolitis syndrome 5. Recently, many genes have also been identified for sepsis diagnosis and prognosis; for example, SeptiCyte Lab combined with CEACAM4, LAMP1, PLA2G7, and PLAC8 genes was identified to determine which patients had sepsis 6. Meanwhile, seven genes (DYRK2, CCNB1IP1, TDRD9, ZAP70, ARL14EP, MDC1, and ADGRE3) had been identified as predictors of septic patients and their relationships with clinical outcomes were observed 7. However, these biomarkers lack sensitivity and specificity and have been analyzed by microarray data 8, 9.

We hypothesized that the pathological process of sepsis changes the transcriptome profile of PBMCs. In this study, we characterized the transcriptome profile of PBMCs in patients with sepsis using high-throughput sequencing to establish alterations present in gene expression patterns and identify potential biomarkers that could serve as a convenient tool to improve sepsis diagnosis.

## Methods

The study was approved by the Clinical Research Ethics Committee of Taizhou Central Hospital (Taizhou University Medical School) (Registration number: 2019-016, Principal investigator: Yinghe Xu, Date of registration: February 26, 2019). This study was registered in the Chinese Clinical Trial Registry (ChiCTR1900022081, Principal investigator: Yinghe Xu, Date of registration: March 21, 2019). The trial was registered prior to patient enrollment. Written informed consent was obtained from all participators or their legal representative.

### Study design

Patients were prospectively screened at 3 participating ICUs and enrolled between April 1, 2019, and November 20, 2019. The last follow-up was completed on February 18, 2020. Adult patients (aged >18 years) who were admitted to the ICU with sepsis were enrolled in the study. Healthy subjects (with 0 in SOFA and without infection) were also recruited as the control group from Physical Examination Center during the same time. Sepsis was defined as the presence of an infection combined with an acute change in SOFA score of 2 or more points 10. Detailed clinical data and outcomes for all enrolled patients were collected and recorded in case report forms at admission and during the 28/90-day follow-up.

### Methods of mRNA sequencing/mRNA sequencing sample preparation

Samples were obtained from 105 patients with an acute change in SOFA score of 2 or more points following admission to ICU, and 10 mL whole blood sample were taken. PBMCs were immediately isolated by Ficoll-PaqueTM PLUS medium (GE Healthcare, Uppsala, Sweden). Eighty-seven patients diagnosed with infection were finally defined as sepsis, and their samples were used for mRNA sequencing or validation, as well as, samples of 40 healthy controls. Total RNA, which was freshly extracted, was stored at -80°C for subsequent testing. A sequencing library was then prepared following the manufacturer's instructions (TruSeqTM Small RNA Sample Preparation Kit, Illumina, San Diego, CA, USA), consisting of purification of poly-A-containing mRNA molecules, fragmentation of mRNA, end repair, addition of a single 'A' base, ligation of adapters, reverse transcription, PCR amplification and pooled gel purification steps. The pooled library consisted of sequences with lengths of approximately 250 nucleotides. The library was sequenced using the HiSeq 2500 sequencing system (Illumina, San Diego, CA, USA).

### Quantitative real-time polymerase chain reaction

Quantitative real-time polymerase chain reaction (qRT-PCR) was used to validate the results of the transcriptome analysis. Total RNA was isolated from PBMCs and reverse transcribed. qRT-PCR was performed using a two-step protocol. Complementary DNA (cDNA) was synthesized using a RevertAid First Strand cDNA Synthesis Kit (Thermo, IL, USA). The amount of cDNA was optimized so that amplification of both control gene cDNA and the cDNAs of interest were in the exponential phase. qRT-PCRs were performed with PowerUp SYBR Green Master Mix (Thermo, IL, USA) using specific gene primers (BioSune, Shanghai, China) following the manufacturer's instructions. The specific quantitative primers for 5 mRNAs are provided in Supplementary Table 1. Thermal cycling was performed using a 7500 Real-Time PCR instrument (ABI, CA, USA). The target genes were assayed in triplicate on each plate. Target mRNA levels were quantified using the comparative Ct method and normalized using glyceraldehyde phosphate dehydrogenase (GAPDH) as a housekeeping gene.

### Enzyme-linked immunosorbent assay

Flow cytometric bead array (CBA) was used as a rapid determination tool for Th1/Th2 cytokines, including IL-2, IL-4, IL-6, IL-10, TNF and IFN-γ. The quantification of the 6 cytokines was evaluated by a FACSAria II Cell Sorter (BD, CA, USA) with a human Th1/Th2 subpopulation detection kit (CEGER, Zhejiang, China). The data of the samples acquired from the FACSAria II were analyzed by BD FCAP Array v3.0.1 software. Six standard curves were obtained from one set of calibrators, and six results were obtained from one test sample. The maximum and minimum limits of the six detected cytokines were 1.0 and 5000 pg/ml, respectively. Biochemical measurement was performed by a Siemens advia2400 (Siemens, BER, GER).

### Bioinformatics

The bioinformatics pipeline used to process RNA-Seq data is presented in Supplementary Figure 1. The read quality of the sequencing data was evaluated by using Fastqc 11. Adapter contamination and low-quality read filtering were performed by using Trimmomatic 12 v0.36 with default parameters. Paired-end reads were mapped to the human genome reference GRCh38.87 by using HISAT 13 v2.0.5. All parameters were set to the default values, including mismatch tolerance. The output SAM files were sorted and converted to BAM files by samtools v1.3.1. Raw read counts of each gene were computed by using HTSeq 14. Differential expression analysis was performed by using DESeq2 15. Genes with low abundance were excluded from downstream analysis if their total expression was <10 and if their expression variance was <10 across samples. Significance was defined as an adjusted P value <0.05, which was determined by using the Benjamini-Hochberg procedure (as implemented in the R function p.adjust) to control type I error in multiple tests. The significantly upregulated or downregulated genes in patients with sepsis were then submitted to ClueGO 16 for annotation enrichment analysis. The enriched Gene Ontology (GO) terms with a false discovery rate of <0.05 were considered significantly enriched.

### Statistical analysis

Data are expressed as the mean ± standard deviation, median with interquartile range or percent with number of patients. Normality was evaluated by using the Shapiro-Wilk test (*P* value >0.05). Comparisons between groups of continuous variables, which were normally distributed, were carried out using Student's t-test. Comparisons of other scenarios were performed using the Mann-Whitney U test. Comparisons of categorical variables were performed using the χ^2^ test. *P* values <0.05 were considered significant.

All statistical analyses were performed by using the R software package. Principal component analysis (PCA) of the different groups' samples was performed on the gene expression matrix by using the 'sva' function 17. Unsupervised hierarchical clustering was performed with all protein-coding genes except low-abundance genes by using the 'pheatmap' function 18, based on Ward's cluster method and the Euclidean distance matrix. The significantly differentially expressed genes (DEGs) were analyzed by using the DESeq2 package. An adjusted *P* value <0.05 was considered to indicate a significant difference, which employed the Benjamini-Hochberg method for multiple-test correction. The DEGs validated via qRT-PCR were identified using Student's t-test. A *P* value <0.05 was considered significant.

## Results

### Patient and clinical characteristics

The demographic and clinical characteristics at admission of patients with sepsis and of the healthy controls whose PBMCs were sequenced are summarized in Table 1. A total of 24 patients with sepsis and 20 healthy controls were enrolled in this study. The SOFA score was 7.6 ± 3.7, and the APACHE II score was 22.3 ± 6.8. Among these patients, one patient had a gram-positive bacterial infection, more than 30% had a gram-negative bacterial infection, and one patient had a viral infection. The short-term (28/90 day) mortality of the patients with sepsis was 29.2%/29.2%. The levels of laboratory indices, including white blood cell count, hemoglobin, hematocrit, platelet count, albumin, aspartate aminotransferase, and creatinine, were significantly worse in the sepsis patients compared to those seen in healthy controls.

### Qualitative analysis of the PBMC transcriptomes of patients with sepsis and healthy controls

To identify changes in the transcriptome profile associated with sepsis, the transcriptomes of PBMCs from 44 subjects (24 patients with sepsis, 20 healthy controls) were characterized. The number of raw paired-end sequencing reads for each sample ranged from 20.8 million to 58.2 million, and the number of clean reads for each sample ranged from 15.1 million to 55.8 million. The average amount of clean data was approximately 36.5 million reads. An average of 97.4% of reads from each sample was uniquely mapped to the human genome. Overall, 58056 genes/19960 protein-coding genes were identified in the patients with sepsis and healthy controls. Detailed information about the qualitative analysis of each sample is recorded in Supplementary Table 2.

### Detection of DEGs in sepsis patients

**Figure 1:**
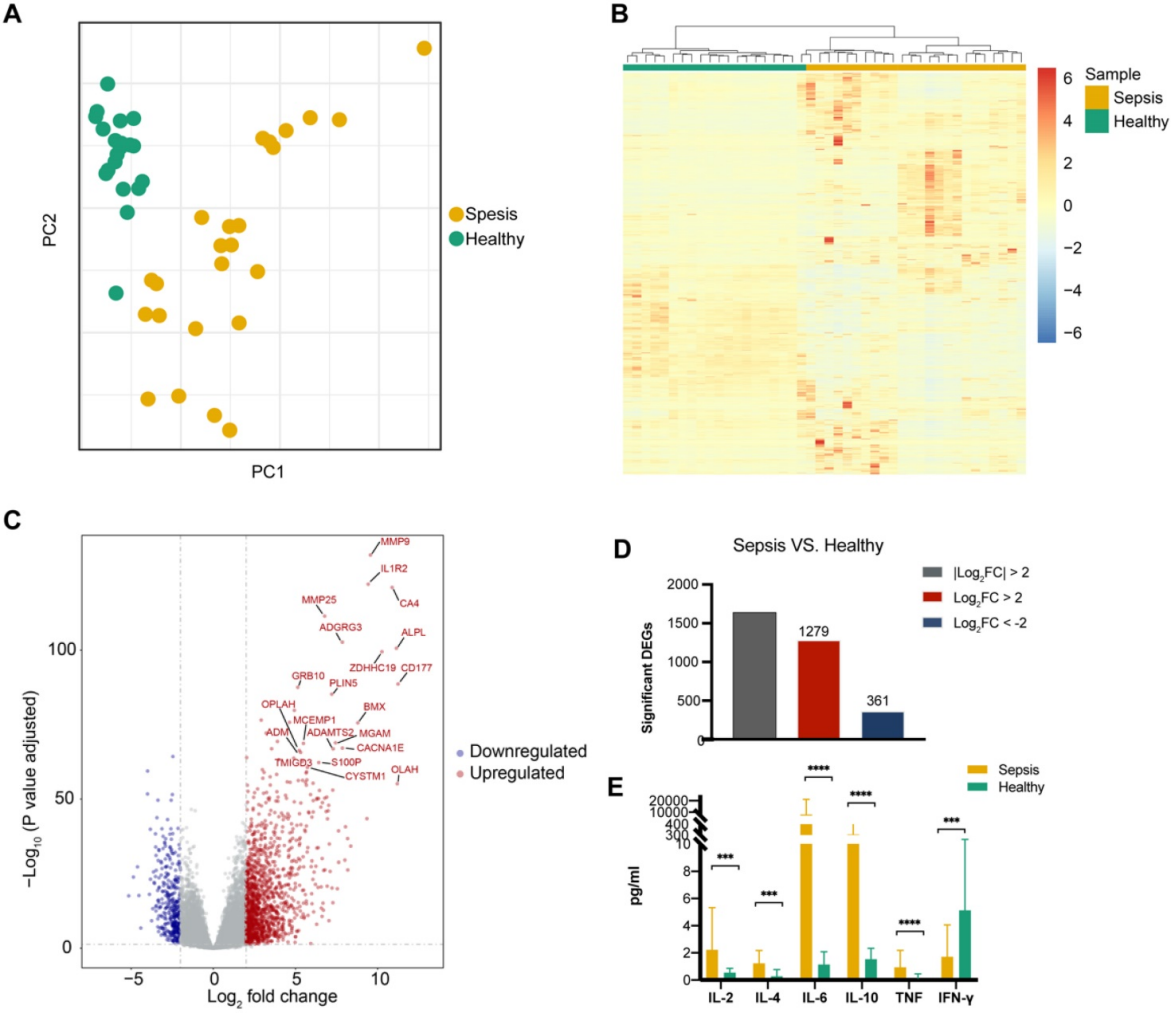
Transcriptional landscape in peripheral blood mononuclear cells (PBMCs) from patients with sepsis and healthy controls. (**A**) Principal component analysis (PCA) of the RNA transcriptome of PBMCs from sepsis patients and healthy subjects. (**B**) Unsupervised hierarchical cluster analysis of sepsis patients and healthy subjects based on the normalized expression of protein-coding genes except the low-abundance genes, the sum and variance of which are lower than 10. Each column represents one sample, and each row represents a gene. The expression level of each gene in a single sample is depicted according to the color scale. (**C**) Volcano plot of differentially expressed genes (DEGs) from the comparison between sepsis patients and healthy subjects. Red coloring shows upregulated genes, and blue coloring shows downregulated genes (|log2 fold change|>2, adjusted P value <0.05). (**D**) Number of up- and downregulated genes, which are colored in red and blue, respectively. (**E**) Enzyme-linked immunosorbent assay (ELISA) measurement of serum IL-2, IL-4, IL-6, IL-10, TNF and IFN-γ levels. *** P value <0.001, **** P value <0.0001; Mann-Whitney U test.

A PCA of global gene expression profiles of PBMCs revealed that sepsis patients were clearly separate from healthy individuals (Figure 1A). Additionally, unsupervised hierarchical cluster analysis based on the normalized expression of protein-coding genes except the low-abundance genes showed that all of the patients with sepsis clustered together (Figure 1B). To determine whether there were distinct patterns of gene expression in sepsis patients, transcriptomic profiles of sepsis patients were compared to those of healthy controls. Differential gene expression analysis showed that sepsis patients had obvious changes in their PBMCs compared to those of healthy subjects (Figure 1C). The expression of 1639 genes was significantly different (using a log2 fold change (log2 fold change) >2 and a Benjamini-Hochberg adjusted P value <0.05) in sepsis patients. Of these genes, 1278 (78.0%) were upregulated and 361 (22.0%) were downregulated (Figure 1D, Supplementary Table 3 and Supplementary Table 4). The expression pattern of DEGs was shown by using unsupervised hierarchical cluster analysis (Supplementary Figure 2).

Significant differences were observed in the expression of the pro-inflammatory cytokine genes IL-2, IL-6, TNF, and IFN-γ and the anti-inflammatory cytokine genes IL-4 and IL-10 between sepsis patients and healthy controls (Figure 1E).

### Annotation enrichment analysis

**Figure 2:**
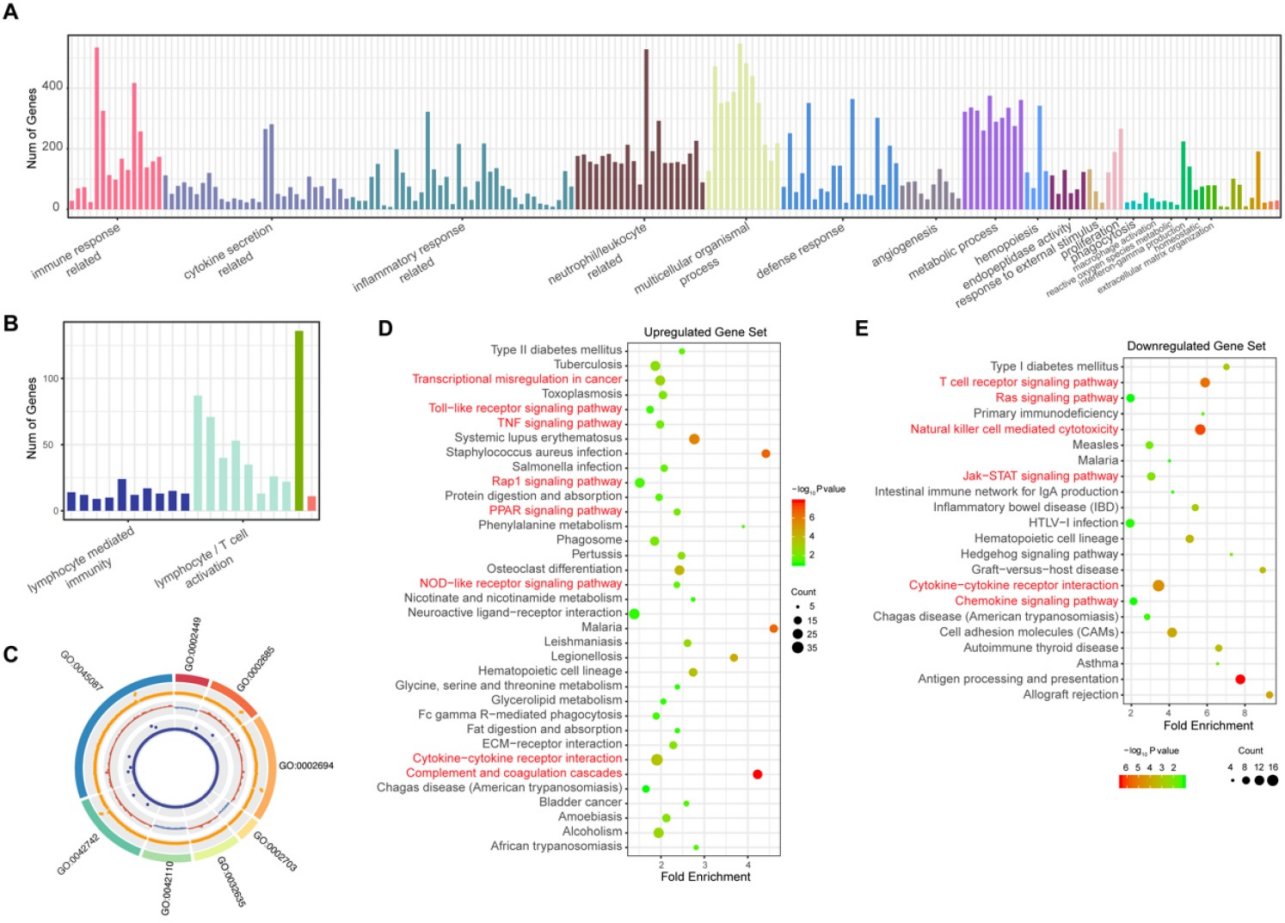
Gene Ontology (GO) and Kyoto Encyclopedia of Genes and Genomes (KEGG) analysis of significantly differentially expressed genes (DEGs) in patients with sepsis and healthy subjects. (**A**) GO terms of the 1279 upregulated genes in sepsis patients. (**B**) GO terms of the 361 downregulated genes in sepsis patients. (**C**) The top 8 GO terms enriched among the up- and downregulated genes. From outside to inside of the circos plot: the most significantly altered GO terms, adjusted P value of DEGs that participated in GO terms, log2 fold change in DEGs, and base mean of DEG expression. (**D-E**) KEGG analysis of the upregulated and downregulated genes.

To elucidate the pathogenesis of sepsis and study the possible functions of these observed changes in gene expression, the biological processes that these DEGs participate in were identified by annotation enrichment analysis using ClueGO. In total, 214 GO terms were identified. The overexpressed genes showed functional significant differences (Benjamini-Hochberg adjusted probability <0.05) related to 194 GO terms, as shown in Figure 2A and detailed in Supplementary Table 5. These GO terms are related to the immune response; cytokine secretion, such as “interleukin-6 production” and “regulation of tumor necrosis factor production”; and the inflammatory response. In addition, biological processes such as the defense response, cell chemotaxis and migration, angiogenesis, hemopoiesis, and metalloendopeptidase activity were also identified among these upregulated genes in sepsis patients. Immune response pathways (Figure 2B, Supplementary Table 6) such as lymphocyte/ (alpha-beta) T cell activation, leukocyte/lymphocyte/natural killer cell-mediated immunity, and the cellular defense response were enriched among downregulated genes. The top eight pathways enriched according to ClueGO are depicted in Figure 2C. These results indicated that excessive inflammation and immune suppression are concurrent in the host response, which is the principal pathophysiology in sepsis. Additionally, to identify whether there were some specific pathways related to changes in the sepsis transcriptome profile, an annotation enrichment analysis was also performed based on the canonical pathways described in the Kyoto Encyclopedia of Genes and Genomes (KEGG) pathway database. The significantly enriched pathways of upregulated DEGs in septic patients compared to those in healthy subjects included “complement and coagulation cascades” (Figure 2D). “T cell receptor signaling pathway” and “natural killer cell-mediated cytotoxicity” were enriched among downregulated DEGs (Figure 2E). Moreover, the TNF signaling pathway, Toll-like receptor signaling pathway and Jak-STAT signaling pathway were also altered in sepsis patient PBMCs.

### Identification of representative and specific genes in sepsis patients

**Figure 3:**
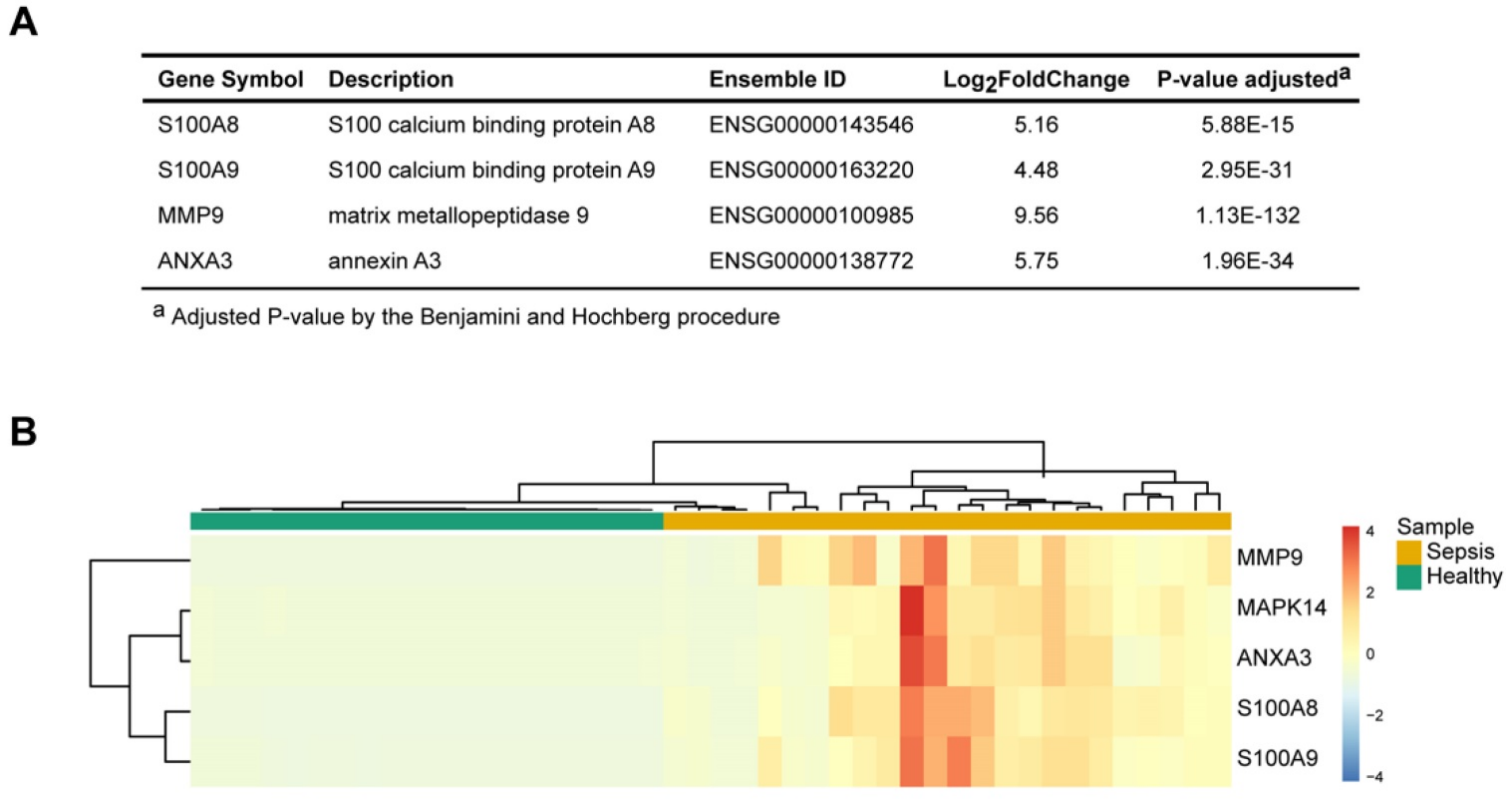
Four pathophysiology-based biomarkers of sepsis. (**A**) Top 4 differentially expressed genes (DEGs) between sepsis patients and healthy subjects, as potential disease biomarkers. The P value was adjusted by the Benjamini-Hochberg procedure. (**B**) Unsupervised hierarchical cluster analysis of sepsis patients and healthy subjects based on the normalized expression of the four biomarkers. Each column represents one sample, and each row represents a gene. The expression level of each gene in a single sample is depicted according to the color scale.

To identify the key molecules that were differentially expressed and associated with sepsis pathophysiology, the differential gene expression profiles between sepsis patients and healthy subjects and their associated functions were used as the foundation for derivation of a candidate sepsis signature and biomarker. The frequencies of all identified DEGs participating in each GO term cluster were calculated. An importance score was defined based on the gene expression, significance of the difference and function participation frequency. Four unique representative genes (matrix metalloendopeptidase 9, MMP9; S100 calcium-binding protein A8, S100A8; S100 calcium-binding protein A9, S100A9; and annexin A3, ANXA3) were found to be correlated with the highest functional importance (Figure 3A). These genes were specifically expressed in the patients with sepsis but were minimally expressed in the healthy controls (Figure 3B). They were related to sepsis pathogenesis; based on functional enrichment analysis, MMP9 has effects on “endothelial barrier dysfunction”, “TNF signaling pathway” and “regulation of defense response”. ANXA3 plays a role in “anticoagulation”, “angiogenesis” and “response to bacterium”. S100A8 and S100A9 have implication on “inflammatory response”, “macrophage activation” and “neutrophil activation” These molecules may serve as signature of sepsis.

### Validation of the mRNA expression levels of four genes using qRT-PCR

**Figure 4:**
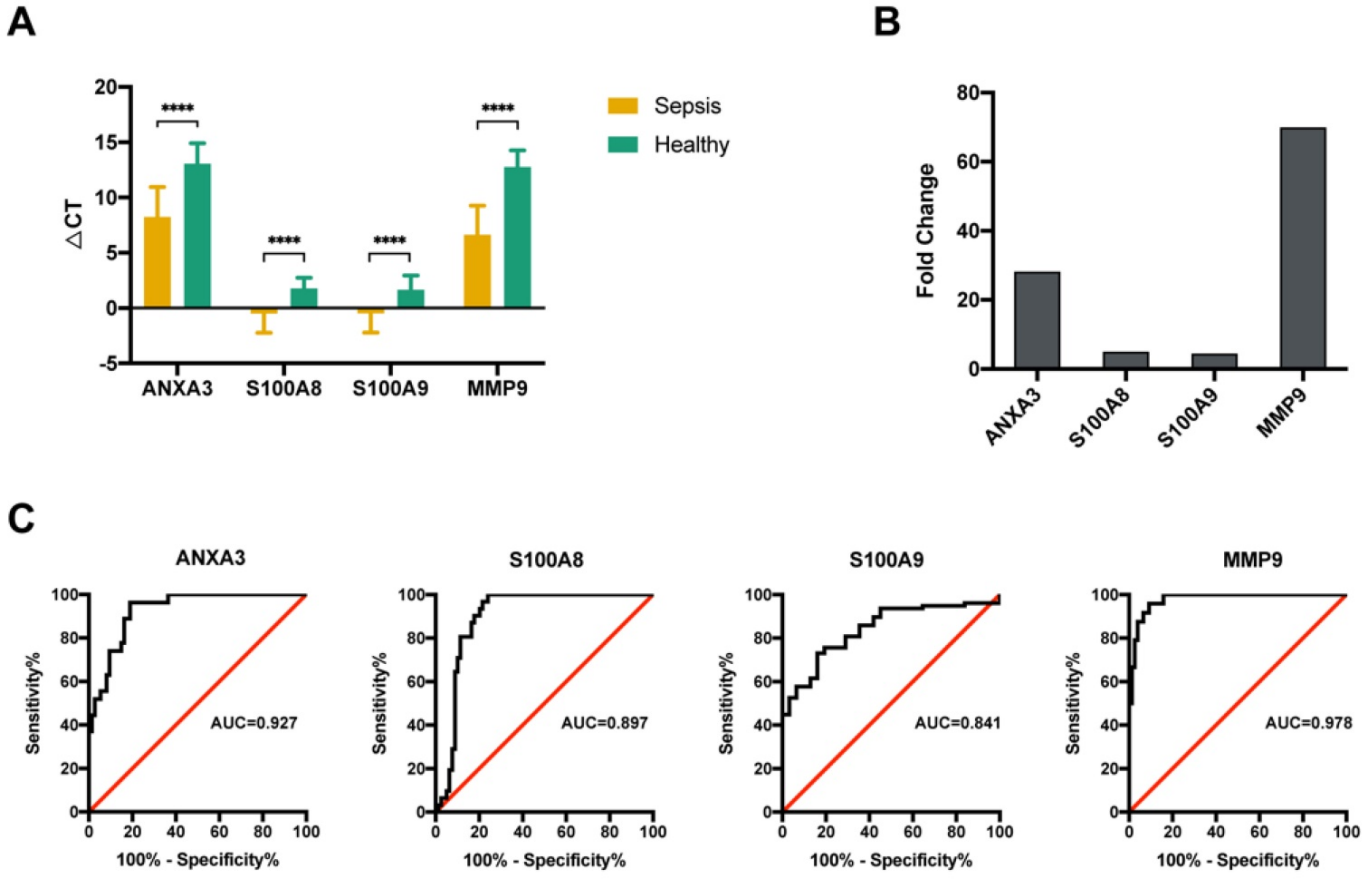
Validation of four potential biomarkers of sepsis. (**A**) The ΔCT results of the top 4 differentially expressed genes (DEGs) identified using quantitative real-time polymerase chain reaction (qRT-PCR). **** P value <0.0001; Mann-Whitney U test. (**B**) Comparison of the fold changes in the top 4 DEGs identified using qRT-PCR between sepsis patients and healthy control subjects. (**C**) ROC curves for the top 4 DEGs: ANXA3, S100A8, S100A9 and MMP9.

The altered expression of the 4 key molecules associated with sepsis was validated by qRT-PCR with PBMCs in the validation group. Samples from another 63 sepsis patients and 20 healthy subjects were subjected to qRT-PCR. The clinical characteristics of the subjects in the validation group were similar to those in the sequencing group (Table 2). The qRT-PCR data indicated that all four genes were significantly upregulated (Figure 4A), which was consistent with the results of the initial screening analysis. Specifically, the expression levels of these 4 genes were increased > 4.0-fold in patients with sepsis compared with those in healthy subjects: ANXA3 (28.1-fold), S100A8 (4.9-fold), S100A9 (4.4-fold), and MMP9 (69.2-fold) (Figure 4B). To investigate the expression levels of these 4 genes as diagnostic biomarkers of sepsis, the qRT-PCR data were subjected to ROC analysis to measure their diagnostic accuracy. As shown in Figure 4C, the areas under the ROCs (AUROCs) of the 4 genes were all > 0.8, especially for ANXA3 and MMP9. These results indicated that, the expression levels of these 4 markers are highly specific and may serve as sensitive biomarkers for predicting septic patients.

## Discussion

In this study, mRNA sequencing was implemented to identify four potential diagnostic biomarkers for sepsis, which were involved in anticoagulation, endothelial barrier dysfunction and inflammatory response. The diagnostic performance of these markers has been validated on independent patients through qRT-PCR-based ROC analysis. Conversion of the classifier from a sequencing format to qRT-PCR format is convenient for clinical utility. These candidates' blood biomarkers assist in the rapid diagnosis of septic patients at ICU admission.

To determine the basic mRNA expression status, this study identified entire mRNAs that changed significantly in the PBMCs of patients with sepsis compared with those in the PBMCs of healthy subjects. According to quality control and qRT-PCR validation, our sequencing results had high reliability and quality. Through bioinformatics analysis, potential functions of significantly DEGs were predicted by GO term enrichment and KEGG pathway enrichment. Identifying important biological function changes could help us to more clearly understand the molecular mechanisms of sepsis.

Based on differential expression analysis, 1639 DEGs were found between the sepsis and healthy groups. GO enrichment analysis was performed and revealed that the biological process terms that were significantly enriched included the immune response, cytokine secretion-related processes, the inflammatory response, and neutrophil/leukocyte-related processes, which are the main pathophysiological conditions of sepsis 19-21. According to the KEGG enrichment results, the Toll-like receptor signaling pathway and TNF signaling pathway were identified in the upregulated gene set. The Jak-STAT signaling pathway, T cell receptor signaling pathway, and natural killer cell-mediated pathway appeared in the downregulated gene set. The potential impact of these pathways in sepsis has already been stated in published studies 22-26. The results showed that genes participating in immune and inflammatory responses have mixed differential expression patterns of both up- and downregulation. These gene expression and functional dysregulation results are consistent with previous studies showing that the host response in sepsis contains both sustained excessive inflammation and immune suppression before returning to normal homeostasis. Apoptosis is another manifest pathological process of sepsis. However, in this study, dysregulation of the genes related to apoptosis was not detected.

The identification of potential biomarkers for sepsis not only improves clinical practice but also provides molecular insights into the pathophysiological basis of this disease. We used a clinical manifestation-directed analysis of the functional synergy among DEGs and discovered four potential biomarkers, which were all validated with external samples. MMP9 has been reported to be involved in extracellular matrix degradation and leukocyte migration, which are essential components of an effective host response to *Escherichia coli* peritonitis 27. In addition, MMP9 has been shown to regulate platelet-dependent infiltration of neutrophils and tissue damage in septic lung injury by controlling CD40L shedding from platelets, which indicates that targeting MMP9 may be a useful strategy to limit acute lung injury in abdominal sepsis 28. S100A8 and S100A9 have been reported to alter MyD88-dependent proinflammatory gene programs, which prevent hyperinflammatory responses without impairing pathogen defense 29. In addition, S100A8 and S100A9 have been demonstrated to mediate endotoxin-induced cardiomyocyte dysfunction via the receptor for advanced glycation end products 30, which may change the condition of cardiomyocyte dysfunction as a result of sepsis, which the leading cause of death in the critically ill. ANXA3 has been reported to play a role in anticoagulation 31. The augmented tendency for thrombosis during sepsis is caused mainly by compromised activity of the three main anticoagulant pathways 21. The level of ANXA3 expression was significantly increased in patients with sepsis, indicating its function in limiting the formation and expansion of thrombosis.

In summary, we performed a comprehensive transcriptome profile analysis of PBMCs to identify the manifest pathological processes of sepsis. Further qRT-PCR validation indicated that four genes are differentially expressed between patients with sepsis and healthy subjects (MMP9, S100A8, S100A9, and ANXA3). These genes have the potential to be treatment targets or biomarkers for sepsis diagnosis and could provide a new direction toward understanding pathological processes. To permit the clinical application of these biomarkers, a larger cohort of sepsis patients' needs to be further validated using high-throughput sequencing. The relationship between infecting microorganisms and the transcriptome profile also needs to be clarified in a further study.

## Supplementary Material

Supplementary figures and tables.

## Figures and Tables

**Table 1:** Characteristics of enrolled patients and healthy subjects included in the sequencing group

	Sepsis	Healthy	*P* value
**n**	24	20	
Male (%)	20 (83.3%)	11 (55.0%)	0.086
Age (years)	76.5 [69.3, 80.3]	69.0 [67.2, 74.0]	0.067
SOFA	7.6 ± 3.7		
APACHE II	22.3 ± 6.8		
**Infection**			
Gram-positive bacteria (%)	1 (4.2%)		
Gram-negative bacteria (%)	9 (37.5%)		
Viral (%)	1 (4.2%)		
Other (%)	13 (54.1%)		
**CRRT**	2 (8.3%)		
**Vasopressors**	16 (66.7%)		
**Mechanical ventilation**	13 (54.2%)		
**Mortality**			
28-day	7 (29.2%)		
90-day	7 (29.2%)		
**Laboratory Data**			
MAP (mmHg)	73.1 ± 16.6		
White blood cell count	12.9 [10.4, 18.2]	5.8 [5.2, 6.6]	<0.001
Hemoglobin (g/L)	114.5 ± 29.4	142.8 ± 20.6	0.001
Hematocrit (%)	35.6 [27.3, 40.9]	44.5 [38.0, 47.2]	0.007
Platelet count	110.3 ± 79.0	212.2 ± 73.1	<0.001
Albumin (g/dL)	25.81 ± 5.09	44.3 ± 2.1	<0.001
Aspartate aminotransferase	44.0 [29.5, 84.5]	16.0 [14.0, 27.0]	<0.001
Alanine aminotransferase	27.0 [17.0, 54.5]	20.0 [17.0, 25.0]	0.150
Total bilirubin (μmol/L)	13.8 [8.0, 18.9]	16.5 [12.0, 20.5]	0.273
Creatinine (μmol/L)	225.5 ± 129.2	69.8 ± 15.6	<0.001
INR	1.2 [1.1, 1.4]		

APACHE II = Acute Physiology and Chronic Health Evaluation II; INR = International Normalized Ratio; MAP = mean arterial pressure; SOFA= Sequential Organ Failure Assessment on day of sampling; Data are expressed as the mean ± standard deviation (SD), median (interquartile range) or number of patients (percentages). The continuous variables were compared by using Student's t-test and the Mann-Whitney U test, and the categorical variables were compared by using the χ^2^ or Fisher's exact test between the discovery and validation groups.

**Table 2:** Characteristics of enrolled patients and healthy subjects included in the validation group

	Sepsis	Healthy	*P* value
**n**	63	20	
Male (%)	29 (46.0%)	12 (60.0%)	0.405
Age (years)	65.5 [55.2, 76.5]	63.5 [57.0, 66.0]	0.142
SOFA	6.0 [4.0, 8.0]		
APACHE II	15.0 [10.7, 22.0]		
**Infection**			
Gram-positive bacteria (%)	9 (14.3%)		
Gram-negative bacteria (%)	25 (39.7%)		
Viral (%)	0		
Other (%)	29 (46.0%)		
**CRRT**	6 (9.5%)		
**Vasopressors**	23 (36.5%)		
**Mechanical ventilation**	22 (34.9%)		
**Mortality**			
28-day	6 (9.5%)		
90-day	7 (11.1%)		
**Laboratory Data**			
MAP (mmHg)	81.2 ± 13.5		
White blood cell count	10.40 [6.30, 17.50]	5.40 [4.45, 6.30]	<0.001
Hemoglobin (g/L)	109.2 ± 30.9	149.9 ± 13.3	<0.001
Hematocrit (%)	32.9 ± 8.6	44.4 ± 3.7	<0.001
Platelet count	134.0 [79.5, 187.0]	228.0 [206.0, 246.0]	<0.001
Albumin (g/dL)	28.0 [25.7, 31.6]	45.7 [45.1, 46.8]	<0.001
Aspartate aminotransferase	47.0 [26.0, 111.5]	20.0 [15.0, 27.5]	<0.001
Alanine aminotransferase	30.0 [16.5, 61.0]	20.0 [18.0, 22.2]	0.052
Total bilirubin (μmol/L)	15.4 [8.7, 25.0]	14.5 [12.0, 18.9]	0.966
Creatinine (μmol/L)	111.0 [76.5, 214.5]	68.5 [57.2, 79.5]	<0.001
INR	1.2 [1.1, 1.3]		

APACHE II = Acute Physiology and Chronic Health Evaluation II; INR = International Normalized Ratio; MAP = mean arterial pressure; SOFA = Sequential Organ Failure Assessment on day of sampling. Data are expressed as the mean ± standard deviation (SD), median (interquartile range) or number of patients (percentages). The continuous variables were compared by using Student's t-test and the Mann-Whitney U test, and the categorical variables were compared by using the χ^2^ or Fisher's exact test between the discovery and validation groups.

## Abbreviations

PBMCs: peripheral blood mononuclear cells
PCA: Principal component analysis
DEGs: differentially expressed genes
GO: Gene Ontology
qRT-PCR: quantitative real-time polymerase chain reaction
ICU: intensive care unit
mRNA: messenger ribonucleic acid
cDNA: complementary DNA
GAPDH: glyceraldehyde phosphate dehydrogenase
CBA: cytometric bead array
KEGG: Kyoto Encyclopedia of Genes and Genomes
MMP9: matrix metalloendopeptidase 9
S100A8: S100 calcium-binding protein A8
S100A9: S100 calcium-binding protein A9
ANXA3: annexin A3
