# Supplementary material for: Comprehensive Transcriptome Profiling of Peripheral Blood Mononuclear Cells from Patients with Sepsis
Source: Int J Med Sci. 2020 Jul 25;17(14):2077–86. doi: 10.7150/ijms.46910 (PMC7484663; doi:10.7150/ijms.46910)
Supplement: Supplementary file 1 — Supplementary figures and tables. [file ijmsv17p2077s1.pdf]

SUPPLEMENTARY MATERIALS

Comprehensive Transcriptome Profiling Of Peripheral Blood Mononuclear Cells From Patients With Sepsis

Tianzhou Wu[1*], Xi Liang[1*], Yongpo Jiang[2*], Qi Chen[1], Huaping Zhang[6], Sheng Zhang[2], Chao Zhang[3], Yuhang Lv[6], Jiaojiao Xin[1,4], Jing Jiang[1,4], Dongyan Shi[1,4], Xin Chen[1,5], Jun Li[1,4], Yinghe Xu[6]

Contents

- Supplementary Figure 1: Flowchart of bioinformatics analysis
- Supplementary Figure 2: Unsupervised hierarchical cluster analysis of 1640 differentially expressed genes
- Supplementary Table 1: qPCR primers used in the study
- Supplementary Table 2: Qualitative analysis results
- Supplementary Table 3: Significantly upregulated genes
- Supplementary Table 4: Significantly downregulated genes
- Supplementary Table 5: Annotation enrichment analysis of the significantly upregulated genes
- Supplementary Table 6: Annotation enrichment analysis of the significantly downregulated genes

SUPPLEMENTARY FIGURES

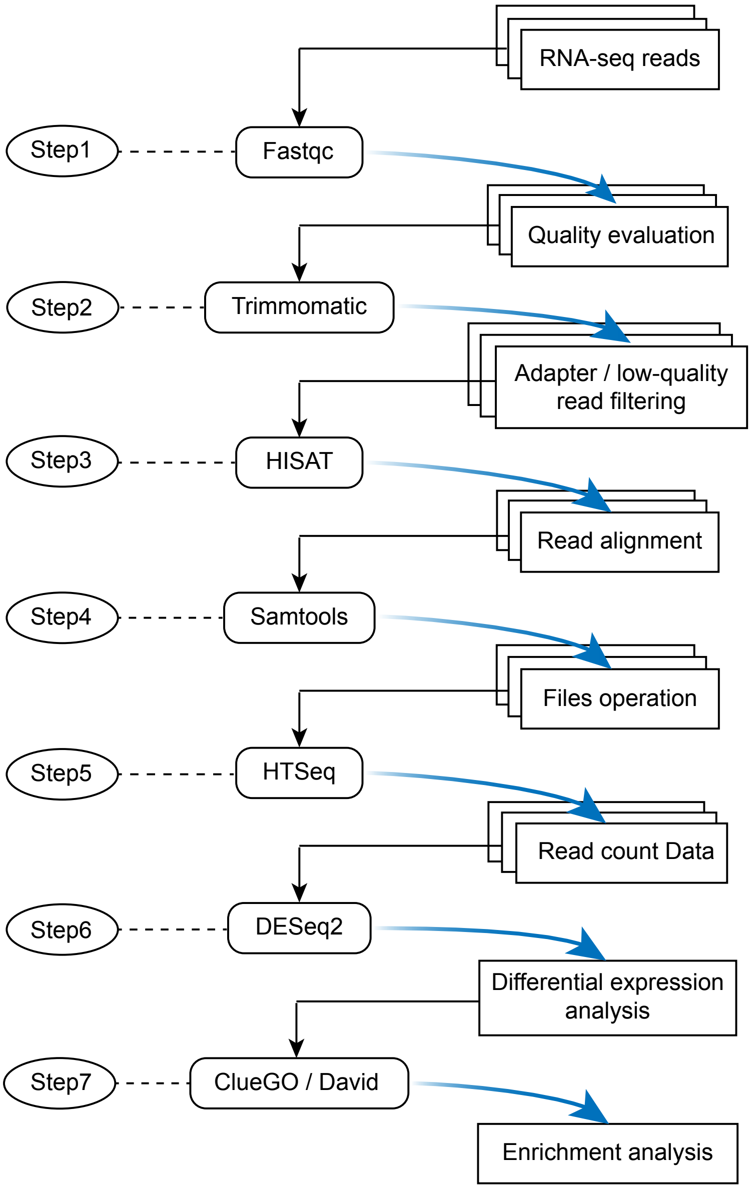

Supplementary Figure 1. Flowchart of bioinformatics analysis.

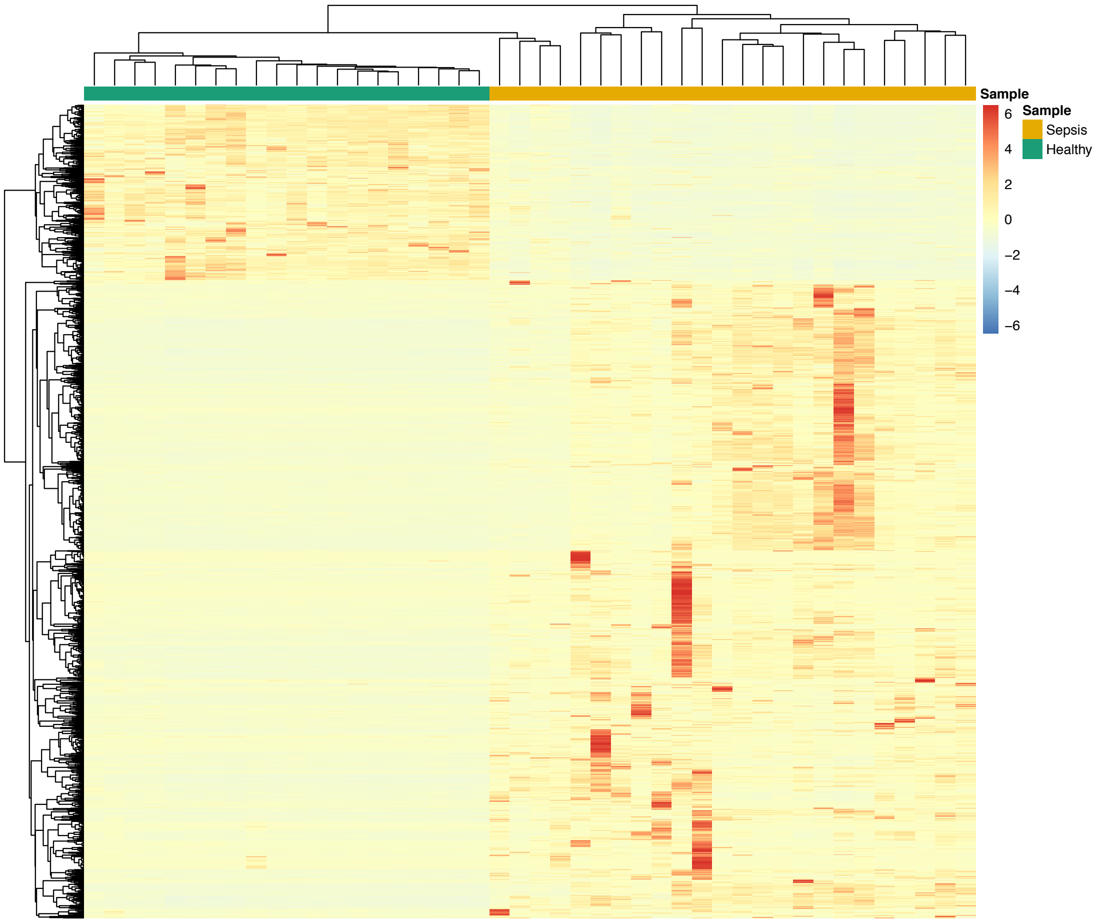

Supplementary Figure 2. Unsupervised hierarchical cluster analysis of sepsis patients and healthy subjects based on the expression of 1640 aberrantly expressed mRNAs in sepsis and normal subjects. Each column represents one sample, and each row represents a gene. The expression level of each gene in a single sample is depicted according to the color scale.

Supplementary Table 1. qPCR primers used in the study

Name		Sequence
GAPDH	F	CTCTCTGCTCCTCCTGTTCG
	R	ACGACCAAATCCGTTGACTC
S100A8	F	ATGCCGTCTACAGGGATGA
	R	TACTCTTTGTGGCTTTCTTC
S100A9	F	TCATCAACACCTTCCACCAA
	R	TTAGCCTCGCCATCAGCA
MMP9	F	CGAACTTTGACAGCGACAAGA
	R	AGGGCGAGGACCATAGAGG
ANXA3	F	ATCTCATGGTGGCCCTAG
	R	ATTTGCCTGCTTGTCCTG

Supplementary Table 2. Qualitative analysis results of transcriptomes of peripheral blood mononuclear cells from patients with sepsis and from healthy subjects

Sample ID	Input Read (PE)	After Trimmomatic	Alignment Rate	Gene Number	PCG Number
S1	29788305	21729543	96.43%	21140	14334
S2	39762278	30403861	97.56%	24941	15341
S3	30551629	22661669	96.59%	20299	14291
S4	35308231	26757420	96.65%	22889	14769
S5	20806190	15127330	95.98%	20945	14334
S6	32399798	24487755	96.70%	18926	13617
S7	42255775	30997790	94.89%	24582	15218
S8	36327346	27360834	96.81%	23287	14940
S9	34426431	33331070	98.30%	26821	16009
S10	28721555	26923052	96.74%	20888	14374
S11	32141442	30509844	98.33%	23087	14832
S12	34289050	32715084	98.34%	24685	15470
S13	56594810	53548224	97.71%	25136	16050
S14	34704033	33387843	98.55%	24081	15150
S15	35735513	34434032	98.27%	25529	15396
S16	33993338	32751432	93.95%	25764	15601
S17	42161651	40787169	98.64%	26446	16183
S18	38468411	37234677	98.77%	24304	15270
S19	41243452	39237996	98.21%	27750	16162
S20	32614121	31182317	91.55%	27652	16558
S21	40487525	39098077	98.75%	26971	16207
S22	33923110	32642532	98.72%	26452	16422
S23	46086493	44536310	98.75%	26897	16276
S24	41605356	40139300	98.88%	24952	15721
H01	51927103	49526672	98.01%	29174	16586
H02	58169222	55767799	98.25%	28708	16712
H03	37642727	36214027	98.31%	28736	16480
H04	39538887	38173431	98.43%	29360	16640
H05	40699574	39216274	98.29%	27773	16505
H06	26886231	25851880	97.66%	27746	16677
H07	30241118	28372100	95.39%	22251	14776
H08	33668190	31812641	97.27%	26258	16365
H09	32634615	31475262	97.28%	26707	15789
H10	34827307	33324668	95.27%	25557	15584
H11	34859893	33632057	96.75%	26670	15781
H12	32449065	31100112	97.19%	25915	15623
H13	32672710	31397204	97.26%	28094	16798

H14	32869932	31692546	96.95%	26392	15720
H15	33605501	32514757	98.38%	25745	15607
H16	33053999	31910143	98.50%	26611	15825
H17	32852502	31299457	97.19%	26441	16530
H18	47228963	45168062	97.41%	23255	15736
H19	33571273	32176433	97.93%	25807	15593
H20	33892555	32662578	98.53%	26442	15764

PCG, Protein-coding gene

Supplementary Table 3. Significantly upregulated genes of comparison between sepsis patients and healthy controls

Ensemble ID	Gene Symbol	log2FoldChange	P-value	P-value adjusted
ENSG00000204936	CD177	11.2454183	1.24E-92	2.33E-89
ENSG00000152463	OLAH	11.19841163	2.24E-58	7.28E-56
ENSG00000162551	ALPL	11.1406467	9.24E-105	2.31E-101
ENSG00000167434	CA4	10.88271017	1.75E-125	8.73E-122
ENSG00000163958	ZDHHC19	10.26253472	1.57E-103	3.36E-100
ENSG00000100985	MMP9	9.563545518	7.58E-137	1.13E-132
ENSG00000115590	IL1R2	9.430487438	9.49E-127	7.10E-123
ENSG00000146122	DAAM2	9.349888759	2.73E-46	3.68E-44
ENSG00000102010	BMX	8.793546181	2.39E-79	2.56E-76
ENSG00000157150	TIMP4	8.365654332	9.46E-37	6.08E-35
ENSG00000257743	MGAM2	8.186090419	1.59E-28	5.34E-27
ENSG00000183578	TNFAIP8L3	8.173736719	2.23E-39	1.79E-37
ENSG00000169174	PCSK9	8.045393744	1.82E-43	2.02E-41
ENSG00000156140	ADAMTS3	8.008678951	6.66E-32	2.94E-30
ENSG00000198216	CACNA1E	7.858749226	9.86E-71	7.77E-68
ENSG00000182885	ADGRG3	7.85626554	6.80E-107	2.04E-103
ENSG00000118113	MMP8	7.634303112	3.77E-31	1.55E-29
ENSG00000162747	FCGR3B	7.624414365	4.86E-42	4.76E-40
ENSG00000124102	PI3	7.551237761	5.67E-22	1.05E-20
ENSG00000257335	MGAM	7.436947174	1.38E-72	1.22E-69
ENSG00000087116	ADAMTS2	7.295687494	1.95E-70	1.39E-67
ENSG00000221937	TAS2R40	7.278293993	4.45E-17	5.31E-16
ENSG00000103723	AP3B2	7.269590192	3.27E-56	9.61E-54
ENSG00000274276	CBSL	7.227524275	3.26E-22	6.21E-21
ENSG00000214456	PLIN5	7.213287369	3.96E-89	5.94E-86
ENSG00000103175	WFDC1	7.112059667	4.91E-28	1.58E-26
ENSG00000180138	CSNK1A1L	7.061159563	1.79E-37	1.22E-35
ENSG00000145384	FABP2	7.039888945	9.81E-11	5.68E-10
ENSG00000113303	BTNL8	7.024302607	6.90E-51	1.22E-48
ENSG00000170439	METTL7B	6.993960194	5.46E-49	8.69E-47
ENSG00000137869	CYP19A1	6.949695319	3.14E-46	4.16E-44
ENSG00000196549	MME	6.930431664	6.85E-23	1.41E-21
ENSG00000111261	MANSC1	6.908537427	8.95E-54	2.16E-51
ENSG00000158089	GALNT14	6.87079286	9.35E-49	1.47E-46
ENSG00000163710	PCOLCE2	6.794490099	1.46E-28	4.92E-27
ENSG00000079393	DUSP13	6.780121871	2.23E-53	5.06E-51
ENSG00000008516	MMP25	6.779208172	9.98E-116	3.74E-112
ENSG00000189221	MAOA	6.715371068	2.43E-28	7.97E-27
ENSG00000283378	CNTNAP3C	6.704788859	1.92E-14	1.70E-13
ENSG00000260234	AC020636.2	6.676983473	6.20E-17	7.25E-16
ENSG00000183762	KREMEN1	6.641826871	8.23E-52	1.54E-49

ENSG00000266265	KLF14	6.624894254	2.04E-32	9.28E-31
ENSG00000164485	IL22RA2	6.617332184	2.91E-12	2.03E-11
ENSG00000162069	BICDL2	6.574520667	3.47E-15	3.36E-14
ENSG00000112333	NR2E1	6.565286151	1.25E-22	2.50E-21
ENSG00000186529	CYP4F3	6.547638705	2.89E-50	4.98E-48
ENSG00000163464	CXCR1	6.465488399	4.51E-56	1.30E-53
ENSG00000163993	S100P	6.418096138	1.03E-65	5.31E-63
ENSG00000140932	CMTM2	6.408209154	3.08E-53	6.59E-51
ENSG00000167994	RAB3IL1	6.383384735	3.58E-36	2.14E-34
ENSG00000267385	AC011498.4	6.374966864	2.01E-22	3.89E-21
ENSG00000179299	NSUN7	6.315865815	2.34E-53	5.22E-51
ENSG00000184611	KCNH7	6.303176697	4.50E-36	2.68E-34
ENSG00000157551	KCNJ15	6.291840893	5.83E-53	1.23E-50
ENSG00000095203	EPB41L4B	6.291100102	1.84E-09	9.17E-09
ENSG00000189127	ANKRD34B	6.245796713	7.87E-30	2.92E-28
ENSG00000129682	FGF13	6.220382245	1.71E-23	3.73E-22
ENSG00000138738	PRDM5	6.134306798	8.89E-28	2.80E-26
ENSG00000162949	CAPN13	6.108501113	7.39E-14	6.12E-13
ENSG00000167676	PLIN4	6.082889952	1.57E-52	3.14E-50
ENSG00000118520	ARG1	6.080043264	1.37E-48	2.14E-46
ENSG00000173535	TNFRSF10C	6.071205435	1.48E-58	4.93E-56
ENSG00000178342	KCNG2	6.060996325	3.12E-25	7.93E-24
ENSG00000137675	MMP27	6.009269125	9.81E-17	1.12E-15
ENSG00000160180	TFF3	6.004943237	1.16E-31	5.05E-30
ENSG00000257017	HP	6.004549088	8.00E-47	1.11E-44
ENSG00000182583	VCX	5.988024661	3.09E-20	5.02E-19
ENSG00000189337	KAZN	5.957839912	1.12E-29	4.14E-28
ENSG00000203857	HSD3B1	5.932639417	0.016112997	0.026991387
ENSG00000135424	ITGA7	5.917988624	1.27E-54	3.18E-52
ENSG00000079385	CEACAM1	5.820886635	3.02E-48	4.56E-46
ENSG00000213937	CLDN9	5.801266291	1.41E-48	2.17E-46
ENSG00000171227	TMEM37	5.767333342	1.29E-12	9.30E-12
ENSG00000138772	ANXA3	5.753156851	3.22E-36	1.96E-34
ENSG00000120306	CYSTM1	5.727344895	4.51E-64	2.25E-61
ENSG00000008438	PGLYRP1	5.714651563	1.15E-34	6.08E-33
ENSG00000108950	FAM20A	5.694844068	9.19E-60	3.53E-57
ENSG00000112053	SLC26A8	5.675984011	1.57E-62	7.10E-60
ENSG00000118785	SPP1	5.67174315	1.03E-16	1.17E-15
ENSG00000163739	CXCL1	5.666240275	9.20E-44	1.04E-41
ENSG00000225873	C3orf86	5.663459137	6.90E-58	2.15E-55
ENSG00000144230	GPR17	5.65937499	1.12E-17	1.41E-16
ENSG00000163221	S100A12	5.649927683	4.21E-62	1.80E-59
ENSG00000171236	LRG1	5.612193459	6.94E-59	2.36E-56
ENSG00000167105	TMEM92	5.602160839	6.19E-20	9.76E-19
ENSG00000159189	C1QC	5.599525249	8.36E-42	8.06E-40

ENSG00000073737	DHRS9	5.570663069	2.27E-42	2.28E-40
ENSG00000002726	AOC1	5.53895352	3.97E-12	2.73E-11
ENSG00000074410	CA12	5.527256439	1.35E-11	8.67E-11
ENSG00000185897	FFAR3	5.500918028	1.70E-21	3.07E-20
ENSG00000108244	KRT23	5.500363353	1.32E-29	4.83E-28
ENSG00000102837	OLFM4	5.488248909	6.21E-17	7.25E-16
ENSG00000183019	MCEMP1	5.485978157	2.45E-72	2.04E-69
ENSG00000112077	RHAG	5.467361441	4.41E-13	3.32E-12
ENSG00000156113	KCNMA1	5.465858602	4.79E-33	2.28E-31
ENSG00000186583	SPATC1	5.434727176	1.35E-59	5.07E-57
ENSG00000121933	TMIGD3	5.423799946	4.42E-66	2.36E-63
ENSG00000137491	SLCO2B1	5.423420262	2.14E-21	3.83E-20
ENSG00000106714	CNTNAP3	5.374472485	5.71E-12	3.84E-11
ENSG00000157152	SYN2	5.333638531	3.28E-44	3.93E-42
ENSG00000139572	GPR84	5.328199185	1.11E-51	2.03E-49
ENSG00000148926	ADM	5.316319562	3.27E-69	2.13E-66
ENSG00000196415	PRTN3	5.297014459	3.55E-14	3.04E-13
ENSG00000152766	ANKRD22	5.289478284	1.24E-39	1.03E-37
ENSG00000133742	CA1	5.275600525	8.94E-16	9.25E-15
ENSG00000229453	SPINK8	5.272207757	1.47E-29	5.34E-28
ENSG00000180871	CXCR2	5.271364257	5.70E-32	2.52E-30
ENSG00000101187	SLCO4A1	5.263814182	1.17E-37	8.09E-36
ENSG00000160471	COX6B2	5.254767856	3.49E-23	7.34E-22
ENSG00000167680	SEMA6B	5.247426756	5.50E-48	8.15E-46
ENSG00000115594	IL1R1	5.237236663	4.98E-37	3.28E-35
ENSG00000178814	OPLAH	5.233452039	6.78E-70	4.61E-67
ENSG00000123836	PFKFB2	5.225417681	4.94E-39	3.89E-37
ENSG00000181856	SLC2A4	5.194619485	3.10E-07	1.14E-06
ENSG00000133800	LYVE1	5.178672964	1.91E-32	8.74E-31
ENSG00000084734	GCKR	5.175194172	7.71E-29	2.65E-27
ENSG00000135636	DYSF	5.171036315	1.61E-59	5.89E-57
ENSG00000126251	GPR42	5.169911666	3.92E-17	4.69E-16
ENSG00000106070	GRB10	5.139689274	1.76E-91	2.92E-88
ENSG00000168903	BTNL3	5.11681493	6.15E-07	2.16E-06
ENSG00000147206	NXF3	5.112748946	3.77E-15	3.65E-14
ENSG00000123610	TNFAIP6	5.111540105	4.37E-27	1.29E-25
ENSG00000101331	CCM2L	5.089049972	8.75E-13	6.42E-12
ENSG00000155659	VSIG4	5.087199174	5.55E-44	6.49E-42
ENSG00000283268	TEX54	5.079412218	4.87E-07	1.74E-06
ENSG00000173239	LIPM	5.057146105	2.22E-31	9.36E-30
ENSG00000156414	TDRD9	5.04798784	9.13E-39	6.91E-37
ENSG00000243137	PSG4	5.013447701	4.99E-05	0.000132132
ENSG00000160200	CBS	5.007162437	1.64E-22	3.22E-21
ENSG00000015413	DPEP1	4.992258499	5.64E-05	0.00014807
ENSG00000175928	LRRN1	4.985882076	7.75E-24	1.75E-22

ENSG00000204414	CSHL1	4.973419467	0.005928973	0.010873294
ENSG00000123119	NECAB1	4.960824822	8.45E-27	2.45E-25
ENSG00000157613	CREB3L1	4.945789258	1.20E-13	9.70E-13
ENSG00000213931	HBE1	4.945669491	1.17E-11	7.55E-11
ENSG00000132170	PPARG	4.931410936	1.17E-83	1.60E-80
ENSG00000079215	SLC1A3	4.927029138	4.47E-52	8.57E-50
ENSG00000174611	KY	4.907976673	7.67E-15	7.09E-14
ENSG00000276644	DACH1	4.905340509	8.19E-32	3.59E-30
ENSG00000105246	EBI3	4.904723529	1.32E-21	2.40E-20
ENSG00000143546	S100A8	4.897436132	3.14E-67	1.81E-64
ENSG00000100234	TIMP3	4.896477254	6.71E-18	8.67E-17
ENSG00000123838	C4BPA	4.849645886	2.19E-15	2.17E-14
ENSG00000099377	HSD3B7	4.848338112	9.26E-43	9.56E-41
ENSG00000101188	NTSR1	4.829660852	1.81E-39	1.48E-37
ENSG00000119121	TRPM6	4.828641772	3.95E-29	1.40E-27
ENSG00000206549	PRSS50	4.808681632	1.28E-08	5.68E-08
ENSG00000062282	DGAT2	4.802247542	1.18E-41	1.11E-39
ENSG00000145491	ROPN1L	4.784819865	3.29E-36	1.99E-34
ENSG00000103154	NECAB2	4.783320836	9.92E-19	1.38E-17
ENSG00000126262	FFAR2	4.778237257	1.63E-31	6.96E-30
ENSG00000111339	ART4	4.77739808	5.17E-08	2.11E-07
ENSG00000170231	FABP6	4.775555182	2.20E-07	8.25E-07
ENSG00000115507	OTX1	4.772989931	1.30E-10	7.49E-10
ENSG00000205502	C2CD4B	4.769129954	1.67E-06	5.53E-06
ENSG00000223401	-	4.763271209	1.04E-26	2.98E-25
ENSG00000173369	C1QB	4.762820681	6.05E-28	1.93E-26
ENSG00000130222	GADD45G	4.760458321	1.63E-44	2.00E-42
ENSG00000203710	CR1	4.745499774	1.93E-29	6.96E-28
ENSG00000163083	INHBB	4.745132152	5.42E-16	5.70E-15
ENSG00000099998	GGT5	4.737686856	2.67E-16	2.91E-15
ENSG00000163421	PROK2	4.69977169	5.71E-29	1.99E-27
ENSG00000128594	LRRC4	4.698480493	8.44E-39	6.41E-37
ENSG00000148346	LCN2	4.695506899	4.77E-27	1.40E-25
ENSG00000227729	RD3L	4.680554444	5.60E-22	1.04E-20
ENSG00000181409	AATK	4.677099611	6.83E-59	2.36E-56
ENSG00000124107	SLPI	4.661272765	1.06E-15	1.09E-14
ENSG00000176788	BASP1	4.659990896	2.40E-41	2.21E-39
ENSG00000112299	VNN1	4.659300438	1.99E-27	6.08E-26
ENSG00000100024	UPB1	4.658686819	9.69E-36	5.58E-34
ENSG00000103569	AQP9	4.655405113	2.49E-38	1.83E-36
ENSG00000163646	CLRN1	4.651016841	4.71E-09	2.22E-08
ENSG00000186417	GLDN	4.640118435	9.27E-26	2.46E-24
ENSG00000159496	RGL4	4.639825519	1.50E-79	1.73E-76
ENSG00000165887	ANKRD2	4.638258628	1.91E-09	9.50E-09
ENSG00000115226	FNDC4	4.634930536	5.93E-11	3.53E-10

ENSG00000180251	SLC9A4	4.627355253	2.31E-10	1.29E-09
ENSG00000113916	BCL6	4.622970371	4.41E-47	6.23E-45
ENSG00000239887	C1orf226	4.604236328	1.69E-33	8.37E-32
ENSG00000069011	PITX1	4.586652718	4.94E-06	1.52E-05
ENSG00000115884	SDC1	4.582758206	4.11E-15	3.95E-14
ENSG00000065618	COL17A1	4.579303529	1.93E-23	4.17E-22
ENSG00000135083	CCNJL	4.578281574	7.46E-33	3.50E-31
ENSG00000151726	ACSL1	4.576418018	3.84E-35	2.12E-33
ENSG00000136160	EDNRB	4.573526701	2.61E-23	5.56E-22
ENSG00000177675	CD163L1	4.570682839	1.61E-27	4.96E-26
ENSG00000235169	SMIM1	4.560884857	2.19E-19	3.28E-18
ENSG00000174705	SH3PXD2B	4.553289602	3.02E-46	4.03E-44
ENSG00000167617	CDC42EP5	4.546592796	0.004262101	0.008029782
ENSG00000169902	TPST1	4.533380663	5.66E-48	8.31E-46
ENSG00000229314	ORM1	4.533094127	8.79E-27	2.54E-25
ENSG00000070729	CNGB1	4.516473399	1.04E-15	1.07E-14
ENSG00000177191	B3GNT8	4.495857947	4.65E-59	1.66E-56
ENSG00000163554	SPTA1	4.495434077	8.93E-17	1.02E-15
ENSG00000138135	CH25H	4.473875339	6.06E-14	5.06E-13
ENSG00000147454	SLC25A37	4.456188265	2.51E-53	5.52E-51
ENSG00000127954	STEAP4	4.450994242	2.16E-23	4.62E-22
ENSG00000188487	INSC	4.446495221	1.49E-17	1.86E-16
ENSG00000073150	PANX2	4.415306348	8.14E-47	1.12E-44
ENSG00000276289	KCNE1B	4.414359401	1.67E-20	2.77E-19
ENSG00000159763	PIP	4.401513221	6.46E-06	1.96E-05
ENSG00000104611	SH2D4A	4.400859264	1.81E-16	2.01E-15
ENSG00000180549	FUT7	4.396676828	2.22E-54	5.46E-52
ENSG00000151952	TMEM132D	4.38824652	6.08E-17	7.13E-16
ENSG00000176907	TCIM	4.374450762	3.15E-06	1.00E-05
ENSG00000136158	SPRY2	4.37346083	1.56E-26	4.40E-25
ENSG00000121742	GJB6	4.354094402	3.46E-17	4.17E-16
ENSG00000157856	DRC1	4.346622885	4.23E-23	8.85E-22
ENSG00000173281	PPP1R3B	4.324073564	5.72E-37	3.74E-35
ENSG00000170091	NSG2	4.32347165	3.76E-14	3.21E-13
ENSG00000179593	ALOX15B	4.31959782	9.08E-20	1.41E-18
ENSG00000242616	GNG10	4.302626349	6.18E-11	3.67E-10
ENSG00000129538	RNASE1	4.280124499	6.56E-29	2.26E-27
ENSG00000140986	RPL3L	4.279567274	1.34E-15	1.36E-14
ENSG00000111058	ACSS3	4.276540737	6.21E-17	7.25E-16
ENSG00000176244	ACBD7	4.265058091	8.13E-10	4.23E-09
ENSG00000206177	HBM	4.262730266	9.59E-08	3.78E-07
ENSG00000162873	KLHDC8A	4.26012997	5.57E-07	1.97E-06
ENSG00000128274	A4GALT	4.258256974	6.42E-05	0.000167186
ENSG00000163220	S100A9	4.255781134	6.37E-46	8.22E-44
ENSG00000172967	XKR3	4.246023029	1.61E-08	7.04E-08

ENSG00000188582	PAQR9	4.243669216	0.000675445	0.001483008
ENSG00000076864	RAP1GAP	4.242760564	1.04E-18	1.44E-17
ENSG00000113763	UNC5A	4.240787921	2.64E-47	3.76E-45
ENSG00000169877	AHSP	4.240224913	2.17E-09	1.07E-08
ENSG00000076826	CAMSAP3	4.235442344	1.17E-28	3.96E-27
ENSG00000173212	MAB21L3	4.23364067	2.88E-11	1.78E-10
ENSG00000183971	NPW	4.221484821	4.90E-12	3.33E-11
ENSG00000170956	CEACAM3	4.221466106	1.97E-43	2.17E-41
ENSG00000228253	MT-ATP8	4.200519959	2.04E-11	1.29E-10
ENSG00000117115	PADI2	4.199110537	8.88E-42	8.47E-40
ENSG00000204421	LY6G6C	4.197854752	1.34E-14	1.21E-13
ENSG00000181577	C6orf223	4.190857904	1.61E-07	6.18E-07
ENSG00000162399	BSND	4.185846839	1.32E-08	5.83E-08
ENSG00000104918	RETN	4.184743753	8.56E-21	1.47E-19
ENSG00000171049	FPR2	4.180388013	1.62E-22	3.20E-21
ENSG00000206172	HBA1	4.167515123	1.14E-08	5.08E-08
ENSG00000159339	PADI4	4.152923889	3.32E-48	4.97E-46
ENSG00000198929	NOS1AP	4.148246506	6.43E-23	1.32E-21
ENSG00000146399	TAAR1	4.147714774	6.43E-06	1.95E-05
ENSG00000109472	CPE	4.142191915	1.55E-20	2.59E-19
ENSG00000180264	ADGRD2	4.134031199	1.02E-07	4.01E-07
ENSG00000054598	FOXC1	4.127282517	9.21E-19	1.28E-17
ENSG00000197993	KEL	4.12625367	7.49E-17	8.68E-16
ENSG00000087245	MMP2	4.087266149	3.82E-11	2.33E-10
ENSG00000258083	OR9A4	4.083561259	5.22E-05	0.000137879
ENSG00000163235	TGFA	4.08300696	4.03E-22	7.64E-21
ENSG00000123342	MMP19	4.045524578	6.96E-24	1.57E-22
ENSG00000116717	GADD45A	4.043945955	2.15E-39	1.73E-37
ENSG00000136634	IL10	4.037155558	1.92E-26	5.39E-25
ENSG00000164100	NDST3	4.025923872	1.32E-11	8.45E-11
ENSG00000112303	VNN2	4.023882563	2.69E-23	5.70E-22
ENSG00000259803	SLC22A31	4.01423805	3.28E-19	4.80E-18
ENSG00000180316	PNPLA1	4.013598126	6.34E-35	3.40E-33
ENSG00000105835	NAMPT	4.011988059	1.36E-19	2.07E-18
ENSG00000173110	HSPA6	4.010791029	2.36E-33	1.15E-31
ENSG00000123700	KCNJ2	4.009943376	3.56E-24	8.20E-23
ENSG00000172179	PRL	4.006832718	1.79E-15	1.79E-14
ENSG00000205642	VCX3B	4.00465311	3.02E-05	8.28E-05
ENSG00000157064	NMNAT2	3.996149685	6.53E-37	4.25E-35
ENSG00000105352	CEACAM4	3.98963068	1.07E-66	5.92E-64
ENSG00000177575	CD163	3.988102533	6.22E-26	1.68E-24
ENSG00000132965	ALOX5AP	3.98662623	6.02E-47	8.43E-45
ENSG00000215009	ACSM4	3.977840409	8.20E-10	4.26E-09
ENSG00000133048	CHI3L1	3.972594625	8.90E-10	4.61E-09
ENSG00000181778	TMEM252	3.964786547	2.98E-07	1.10E-06

ENSG00000163283	ALPP	3.95877208	0.000185205	0.000449548
ENSG00000273111	LYPD4	3.958350655	6.84E-08	2.76E-07
ENSG00000124875	CXCL6	3.957453011	2.87E-10	1.58E-09
ENSG00000135480	KRT7	3.956197663	3.60E-13	2.74E-12
ENSG00000009950	MLXIPL	3.951182729	4.13E-20	6.66E-19
ENSG00000171557	FGG	3.94605275	9.35E-06	2.77E-05
ENSG00000258223	PRSS58	3.940142452	0.002048132	0.00408971
ENSG00000123405	NFE2	3.939815867	9.28E-54	2.21E-51
ENSG00000119535	CSF3R	3.932713706	2.75E-52	5.34E-50
ENSG00000137261	KIAA0319	3.928327633	9.21E-12	6.04E-11
ENSG00000012223	LTF	3.927866589	5.26E-13	3.94E-12
ENSG00000115271	GCA	3.925390686	1.04E-28	3.54E-27
ENSG00000110079	MS4A4A	3.921933387	2.16E-26	6.05E-25
ENSG00000173376	NDNF	3.920920834	9.33E-05	0.000236795
ENSG00000115598	IL1RL2	3.907560906	7.27E-09	3.34E-08
ENSG00000100365	NCF4	3.904949211	4.76E-73	4.46E-70
ENSG00000184389	A3GALT2	3.898410733	5.21E-16	5.49E-15
ENSG00000143797	MBOAT2	3.896313482	2.16E-36	1.34E-34
ENSG00000100504	PYGL	3.894272901	1.53E-39	1.26E-37
ENSG00000125780	TGM3	3.888704637	8.46E-12	5.58E-11
ENSG00000105610	KLF1	3.885270762	9.20E-14	7.52E-13
ENSG00000196923	PDLIM7	3.870592685	1.20E-55	3.32E-53
ENSG00000134873	CLDN10	3.866699749	5.95E-07	2.10E-06
ENSG00000130513	GDF15	3.866616362	1.53E-15	1.54E-14
ENSG00000178896	EXOSC4	3.863295772	2.82E-35	1.56E-33
ENSG00000165626	BEND7	3.859360101	1.79E-20	2.97E-19
ENSG00000134827	TCN1	3.856832648	1.66E-11	1.05E-10
ENSG00000136630	HLX	3.855109374	1.29E-61	5.38E-59
ENSG00000101460	MAP1LC3A	3.849375403	2.38E-34	1.25E-32
ENSG00000248099	INSL3	3.84210248	1.15E-22	2.30E-21
ENSG00000133101	CCNA1	3.84055105	4.19E-14	3.56E-13
ENSG00000182175	RGMA	3.839409414	2.89E-22	5.53E-21
ENSG00000087085	ACHE	3.837875705	5.78E-06	1.77E-05
ENSG00000282988	AL031777.3	3.837014398	2.27E-13	1.77E-12
ENSG00000185201	IFITM2	3.833160507	4.99E-44	5.88E-42
ENSG00000142025	DMRTC2	3.830232374	2.26E-06	7.33E-06
ENSG00000133063	CHIT1	3.827781151	9.33E-21	1.58E-19
ENSG00000114656	KIAA1257	3.824970293	7.79E-14	6.43E-13
ENSG00000165181	SHOC1	3.820656052	5.06E-26	1.39E-24
ENSG00000115457	IGFBP2	3.815289205	3.69E-14	3.16E-13
ENSG00000154277	UCHL1	3.811465147	9.11E-12	5.98E-11
ENSG00000198736	MSRB1	3.810929956	2.17E-52	4.28E-50
ENSG00000205358	MT1H	3.801606514	2.68E-05	7.39E-05
ENSG00000233608	TWIST2	3.79965811	4.26E-11	2.59E-10
ENSG00000179862	CITED4	3.793792513	4.20E-22	7.94E-21

ENSG00000110237	ARHGEF17	3.785917736	7.86E-34	3.95E-32
ENSG00000260314	MRC1	3.770847535	3.20E-14	2.75E-13
ENSG00000135903	PAX3	3.767896968	3.38E-05	9.19E-05
ENSG00000261701	HPR	3.764102439	1.24E-10	7.11E-10
ENSG00000204839	MROH6	3.762565581	5.05E-43	5.36E-41
ENSG00000196517	SLC6A9	3.756276313	1.57E-06	5.20E-06
ENSG00000166527	CLEC4D	3.756108846	4.90E-30	1.86E-28
ENSG00000163959	SLC51A	3.750577207	1.55E-41	1.44E-39
ENSG00000281227	-	3.747078012	1.78E-05	5.05E-05
ENSG00000166825	ANPEP	3.742302096	2.23E-48	3.41E-46
ENSG00000152760	TCTEX1D1	3.740872977	7.83E-22	1.44E-20
ENSG00000145423	SFRP2	3.736013271	2.25E-07	8.44E-07
ENSG00000173334	TRIB1	3.729715331	2.51E-31	1.05E-29
ENSG00000197061	HIST1H4C	3.729618465	6.04E-12	4.04E-11
ENSG00000134571	MYBPC3	3.727002822	1.22E-40	1.06E-38
ENSG00000131435	PDLIM4	3.720208338	2.24E-06	7.27E-06
ENSG00000116991	SIPA1L2	3.720052874	2.69E-38	1.96E-36
ENSG00000134755	DSC2	3.718965573	1.83E-17	2.28E-16
ENSG00000170180	GYPA	3.715072332	8.72E-07	3.01E-06
ENSG00000142549	IGLON5	3.713702375	4.09E-09	1.94E-08
ENSG00000164850	GPER1	3.695919507	1.50E-27	4.63E-26
ENSG00000124839	RAB17	3.690348386	1.37E-11	8.78E-11
ENSG00000124490	CRISP2	3.689291723	2.84E-06	9.08E-06
ENSG00000198363	ASPH	3.686280263	2.44E-27	7.39E-26
ENSG00000102755	FLT1	3.683243019	4.10E-19	5.93E-18
ENSG00000277157	HIST1H4D	3.682444768	1.63E-15	1.64E-14
ENSG00000166926	MS4A6E	3.682268035	7.76E-06	2.32E-05
ENSG00000124233	SEMG1	3.682106642	2.45E-05	6.79E-05
ENSG00000165131	LLCFC1	3.670306332	1.64E-14	1.46E-13
ENSG00000151948	GLT1D1	3.666986932	1.19E-41	1.12E-39
ENSG00000161040	FBXL13	3.660261654	5.09E-15	4.83E-14
ENSG00000170801	HTRA3	3.654081307	9.26E-10	4.79E-09
ENSG00000163697	APBB2	3.653096166	1.29E-28	4.37E-27
ENSG00000197506	SLC28A3	3.646899573	1.48E-20	2.48E-19
ENSG00000132688	NES	3.643053393	3.22E-09	1.55E-08
ENSG00000133116	KL	3.642550966	2.41E-14	2.11E-13
ENSG00000122420	PTGFR	3.640359528	1.71E-19	2.59E-18
ENSG00000254521	SIGLEC12	3.638936254	1.95E-11	1.23E-10
ENSG00000117984	CTSD	3.637609675	4.68E-37	3.10E-35
ENSG00000128918	ALDH1A2	3.635712752	6.20E-19	8.78E-18
ENSG00000092067	CEBPE	3.63172278	3.62E-20	5.86E-19
ENSG00000122733	PHF24	3.617633054	8.14E-08	3.25E-07
ENSG00000197561	ELANE	3.612858472	1.31E-09	6.63E-09
ENSG00000180509	KCNE1	3.612368976	2.50E-31	1.05E-29
ENSG00000106178	CCL24	3.59237767	6.76E-11	4.00E-10

ENSG00000205517	RGL3	3.588913347	5.75E-24	1.31E-22
ENSG00000259823	LYPD8	3.586569725	1.95E-14	1.72E-13
ENSG00000176597	B3GNT5	3.582643404	1.22E-15	1.24E-14
ENSG00000123689	G0S2	3.572141397	5.66E-12	3.81E-11
ENSG00000136488	CSH1	3.571488258	0.001992459	0.003990248
ENSG00000131187	F12	3.567369416	2.41E-36	1.48E-34
ENSG00000164741	DLC1	3.564310796	9.40E-28	2.95E-26
ENSG00000168779	SHOX2	3.562863024	1.45E-14	1.30E-13
ENSG00000182310	SPACA6	3.561144572	5.11E-21	8.89E-20
ENSG00000283384	AL138694.1	3.554396864	1.36E-11	8.75E-11
ENSG00000118898	PPL	3.550675984	1.53E-12	1.10E-11
ENSG00000113749	HRH2	3.547469039	1.69E-70	1.27E-67
ENSG00000167874	TMEM88	3.542206205	1.59E-23	3.49E-22
ENSG00000175003	SLC22A1	3.539218356	1.93E-20	3.19E-19
ENSG00000108342	CSF3	3.539103341	0.002514547	0.004938075
ENSG00000012171	SEMA3B	3.529591225	5.42E-16	5.70E-15
ENSG00000124785	NRN1	3.526105509	1.53E-15	1.54E-14
ENSG00000173262	SLC2A14	3.523832456	5.89E-10	3.12E-09
ENSG00000145555	MYO10	3.519606333	1.85E-39	1.50E-37
ENSG00000183856	IQGAP3	3.516838069	2.78E-15	2.72E-14
ENSG00000206047	DEFA1	3.513638881	7.74E-10	4.04E-09
ENSG00000136026	CKAP4	3.510827283	9.41E-45	1.16E-42
ENSG00000171916	LGALS9C	3.508021627	1.38E-15	1.39E-14
ENSG00000129195	PIMREG	3.506874552	1.85E-12	1.31E-11
ENSG00000173372	C1QA	3.506614599	2.01E-16	2.23E-15
ENSG00000169385	RNASE2	3.504106049	4.78E-34	2.44E-32
ENSG00000111052	LIN7A	3.50380066	1.84E-25	4.79E-24
ENSG00000204577	LILRB3	3.503507755	4.05E-49	6.59E-47
ENSG00000198734	F5	3.502759943	1.69E-22	3.32E-21
ENSG00000188175	HEPACAM2	3.498988763	5.82E-07	2.06E-06
ENSG00000281676	-	3.494154611	1.14E-05	3.35E-05
ENSG00000092758	COL9A3	3.488644278	1.84E-34	9.67E-33
ENSG00000170965	PLAC1	3.488518378	0.001918515	0.003853501
ENSG00000170837	GPR27	3.484347701	7.81E-16	8.11E-15
ENSG00000105501	SIGLEC5	3.482923867	7.59E-19	1.06E-17
ENSG00000179564	LSMEM2	3.476792136	1.86E-07	7.06E-07
ENSG00000163803	PLB1	3.475717253	7.90E-38	5.58E-36
ENSG00000172986	GXYLT2	3.47506214	8.92E-08	3.53E-07
ENSG00000120051	CFAP58	3.471087459	1.61E-28	5.40E-27
ENSG00000134686	PHC2	3.469964134	6.10E-58	1.94E-55
ENSG00000158104	HPD	3.463502246	5.14E-21	8.93E-20
ENSG00000072952	MRVI1	3.463326832	3.68E-33	1.78E-31
ENSG00000272398	CD24	3.463273225	8.08E-14	6.66E-13
ENSG00000160883	HK3	3.460258022	8.41E-61	3.41E-58
ENSG00000173597	SULT1B1	3.454928606	7.19E-25	1.76E-23

ENSG00000213853	EMP2	3.452044676	1.28E-05	3.72E-05
ENSG00000004939	SLC4A1	3.451170452	4.56E-08	1.88E-07
ENSG00000124092	CTCFL	3.442091889	4.40E-07	1.58E-06
ENSG00000196358	NTNG2	3.441822363	3.44E-30	1.31E-28
ENSG00000139567	ACVRL1	3.439410987	1.86E-22	3.63E-21
ENSG00000163754	GYG1	3.438817354	3.64E-27	1.08E-25
ENSG00000067798	NAV3	3.434302207	2.54E-10	1.41E-09
ENSG00000143545	RAB13	3.433454514	1.05E-25	2.77E-24
ENSG00000197208	SLC22A4	3.424145955	3.07E-36	1.88E-34
ENSG00000244482	LILRA6	3.419975349	1.56E-53	3.60E-51
ENSG00000142657	PGD	3.419864946	1.04E-47	1.49E-45
ENSG00000144671	SLC22A14	3.415677902	9.90E-12	6.47E-11
ENSG00000131471	AOC3	3.410351144	3.36E-28	1.09E-26
ENSG00000087842	PIR	3.398371662	2.35E-14	2.06E-13
ENSG00000186431	FCAR	3.397845507	9.69E-16	9.99E-15
ENSG00000213722	DDAH2	3.397842619	1.03E-52	2.14E-50
ENSG00000007866	TEAD3	3.396902466	7.26E-09	3.33E-08
ENSG00000167768	KRT1	3.392455371	2.32E-06	7.51E-06
ENSG00000162520	SYNC	3.391742498	5.65E-05	0.000148231
ENSG00000093134	VNN3	3.389740376	9.04E-25	2.20E-23
ENSG00000184270	HIST2H2AB	3.385353618	7.58E-07	2.64E-06
ENSG00000166183	ASPG	3.374888595	1.27E-07	4.93E-07
ENSG00000256812	CAPNS2	3.371082968	1.82E-13	1.43E-12
ENSG00000225940	C5orf67	3.367550941	3.55E-18	4.71E-17
ENSG00000221826	PSG3	3.367301016	0.001753268	0.003544409
ENSG00000011201	ANOS1	3.365978532	7.38E-09	3.38E-08
ENSG00000223609	HBD	3.362654885	2.28E-09	1.13E-08
ENSG00000270168	AC004233.2	3.3619995	4.95E-09	2.32E-08
ENSG00000112195	TREML2	3.356082583	2.35E-22	4.53E-21
ENSG00000166900	STX3	3.352190492	8.09E-31	3.21E-29
ENSG00000108309	RUNDC3A	3.347487801	4.12E-12	2.83E-11
ENSG00000130234	ACE2	3.342908739	4.25E-08	1.76E-07
ENSG00000203814	HIST2H2BF	3.337341188	6.39E-22	1.18E-20
ENSG00000170525	PFKFB3	3.336711675	5.04E-20	8.01E-19
ENSG00000012779	ALOX5	3.336557498	2.91E-53	6.32E-51
ENSG00000278463	HIST1H2AB	3.335761467	8.48E-05	0.000216465
ENSG00000175793	SFN	3.334972184	7.84E-15	7.24E-14
ENSG00000258227	CLEC5A	3.333580771	6.00E-19	8.51E-18
ENSG00000184005	ST6GALNAC3	3.33097003	5.97E-31	2.42E-29
ENSG00000136378	ADAMTS7	3.33045108	3.91E-08	1.63E-07
ENSG00000174885	NLRP6	3.3273988	6.98E-19	9.77E-18
ENSG00000019169	MARCO	3.318409569	3.99E-16	4.25E-15
ENSG00000171560	FGA	3.318048826	2.90E-05	7.97E-05
ENSG00000108688	CCL7	3.317481215	0.011870063	0.020512674
ENSG00000158825	CDA	3.303525935	8.37E-36	4.90E-34

ENSG00000135842	FAM129A	3.299427511	4.24E-19	6.11E-18
ENSG00000113073	SLC4A9	3.29900209	5.29E-10	2.81E-09
ENSG00000134463	ECHDC3	3.291909705	7.37E-33	3.47E-31
ENSG00000224940	PRRT4	3.289655704	5.77E-11	3.44E-10
ENSG00000124159	MATN4	3.286970157	6.80E-05	0.000176189
ENSG00000163625	WDFY3	3.283396623	3.33E-24	7.72E-23
ENSG00000274997	HIST1H2AH	3.276582547	0.001849418	0.003725207
ENSG00000130766	SESN2	3.27461158	2.03E-24	4.83E-23
ENSG00000140379	BCL2A1	3.273981251	3.84E-23	8.05E-22
ENSG00000171051	FPR1	3.271914677	2.03E-26	5.69E-25
ENSG00000169627	BOLA2B	3.269155368	1.38E-06	4.63E-06
ENSG00000249437	NAIP	3.267352147	2.23E-21	3.98E-20
ENSG00000172594	SMPDL3A	3.263379238	3.51E-16	3.78E-15
ENSG00000073331	ALPK1	3.253728862	5.92E-44	6.82E-42
ENSG00000187513	GJA4	3.25263167	2.47E-10	1.37E-09
ENSG00000166265	CYYR1	3.25222641	3.60E-19	5.24E-18
ENSG00000167100	SAMD14	3.250169471	3.54E-13	2.70E-12
ENSG00000198723	TEX45	3.2485172	3.34E-05	9.09E-05
ENSG00000115604	IL18R1	3.248139	5.39E-15	5.08E-14
ENSG00000188523	CFAP77	3.247009656	1.67E-05	4.77E-05
ENSG00000007968	E2F2	3.242225179	2.18E-55	5.93E-53
ENSG00000188305	PEAK3	3.242194419	1.97E-31	8.35E-30
ENSG00000183873	SCN5A	3.241453785	1.59E-09	7.97E-09
ENSG00000108984	MAP2K6	3.240641741	1.73E-29	6.27E-28
ENSG00000105825	TFPI2	3.228160243	0.000296118	0.000692933
ENSG00000058085	LAMC2	3.227564292	2.41E-10	1.34E-09
ENSG00000070731	ST6GALNAC2	3.226621786	4.68E-21	8.17E-20
ENSG00000059804	SLC2A3	3.22586466	5.74E-25	1.41E-23
ENSG00000197721	CR1L	3.224707763	1.94E-10	1.10E-09
ENSG00000229894	GK3P	3.222929815	2.70E-10	1.49E-09
ENSG00000146094	DOK3	3.221403857	7.16E-76	7.15E-73
ENSG00000188897	AC099489.1	3.218434359	1.30E-15	1.33E-14
ENSG00000121900	TMEM54	3.21557847	0.013941986	0.023683188
ENSG00000182759	MAFA	3.214154424	1.22E-05	3.55E-05
ENSG00000187116	LILRA5	3.213200971	5.36E-32	2.38E-30
ENSG00000018280	SLC11A1	3.203274573	8.41E-52	1.56E-49
ENSG00000128965	CHAC1	3.201746217	4.07E-07	1.47E-06
ENSG00000130202	NECTIN2	3.194138543	1.91E-33	9.33E-32
ENSG00000112041	TULP1	3.19027839	2.04E-05	5.71E-05
ENSG00000181274	FRAT2	3.184995582	6.69E-24	1.52E-22
ENSG00000153208	MERTK	3.181211404	2.44E-34	1.27E-32
ENSG00000188056	TREML4	3.17996788	3.12E-09	1.50E-08
ENSG00000175274	TP53I11	3.178762063	3.92E-37	2.61E-35
ENSG00000114270	COL7A1	3.178729612	8.84E-23	1.80E-21
ENSG00000198019	FCGR1B	3.175086878	2.52E-27	7.57E-26

ENSG00000087903	RFX2	3.170904024	5.82E-43	6.13E-41
ENSG00000135451	TROAP	3.169238613	8.27E-18	1.06E-16
ENSG00000120696	KBTBD7	3.168189491	6.02E-17	7.07E-16
ENSG00000183160	TMEM119	3.164788248	1.58E-11	1.01E-10
ENSG00000162745	OLFML2B	3.163525815	3.74E-15	3.62E-14
ENSG00000008056	SYN1	3.159854443	3.58E-13	2.73E-12
ENSG00000198053	SIRPA	3.158254927	1.45E-45	1.86E-43
ENSG00000100218	RSPH14	3.157146446	9.35E-15	8.54E-14
ENSG00000134815	DHX34	3.153981966	1.76E-36	1.11E-34
ENSG00000124588	NQO2	3.148192159	1.43E-39	1.18E-37
ENSG00000130167	TSPAN16	3.147450921	6.73E-14	5.60E-13
ENSG00000146592	CREB5	3.140224763	3.83E-21	6.72E-20
ENSG00000085117	CD82	3.139470457	2.77E-41	2.53E-39
ENSG00000186205	1-Mar	3.137247039	1.28E-39	1.07E-37
ENSG00000188536	HBA2	3.129472964	4.90E-07	1.75E-06
ENSG00000125505	MBOAT7	3.125515983	6.46E-36	3.83E-34
ENSG00000132470	ITGB4	3.123479594	2.19E-14	1.93E-13
ENSG00000196663	TECPR2	3.120370071	1.05E-28	3.59E-27
ENSG00000131969	ABHD12B	3.117769449	2.45E-20	4.01E-19
ENSG00000180953	ST20	3.116294426	6.00E-30	2.26E-28
ENSG00000174358	SLC6A19	3.115020055	0.000148565	0.000365891
ENSG00000127946	HIP1	3.107057918	3.21E-25	8.13E-24
ENSG00000169181	GSG1L	3.107045031	0.000289584	0.000679127
ENSG00000141526	SLC16A3	3.105397859	5.01E-43	5.35E-41
ENSG00000142910	TINAGL1	3.097278758	0.000190669	0.000461913
ENSG00000169891	REPS2	3.095611972	1.82E-23	3.97E-22
ENSG00000086506	HBQ1	3.095334459	4.44E-07	1.60E-06
ENSG00000115828	QPCT	3.095133297	1.25E-13	1.00E-12
ENSG00000172889	EGFL7	3.094457046	3.11E-12	2.16E-11
ENSG00000142405	NLRP12	3.094167661	2.68E-44	3.24E-42
ENSG00000159556	ISL2	3.093639592	4.74E-20	7.55E-19
ENSG00000175063	UBE2C	3.093490832	1.34E-19	2.05E-18
ENSG00000166394	CYB5R2	3.092931808	2.62E-17	3.20E-16
ENSG00000115129	TP53I3	3.087682436	2.59E-62	1.14E-59
ENSG00000157343	ARMC12	3.087096299	4.10E-26	1.13E-24
ENSG00000273983	HIST1H3G	3.086705827	3.77E-08	1.58E-07
ENSG00000115602	IL1RL1	3.085597092	1.73E-08	7.55E-08
ENSG00000132357	CARD6	3.08527632	1.52E-27	4.69E-26
ENSG00000101236	RNF24	3.082147212	1.10E-34	5.85E-33
ENSG00000008323	PLEKHG6	3.078742889	4.75E-11	2.87E-10
ENSG00000150510	FAM124A	3.077462034	2.34E-08	1.00E-07
ENSG00000283632	EXOC3L2	3.071144541	1.06E-08	4.73E-08
ENSG00000153093	ACOXL	3.070230444	7.96E-15	7.33E-14
ENSG00000158578	ALAS2	3.063090111	2.02E-06	6.62E-06
ENSG00000186185	KIF18B	3.061889308	2.82E-16	3.07E-15

ENSG00000181444	ZNF467	3.059953718	4.31E-55	1.13E-52
ENSG00000198814	GK	3.051930778	1.52E-18	2.08E-17
ENSG00000269028	MTRNR2L12	3.050239993	3.82E-08	1.60E-07
ENSG00000110245	APOC3	3.049738012	0.002565391	0.005030007
ENSG00000173890	GPR160	3.038514531	7.67E-14	6.34E-13
ENSG00000205364	MT1M	3.035704198	0.002077593	0.004142461
ENSG00000178026	LRRC75B	3.033451974	6.02E-29	2.09E-27
ENSG00000213088	ACKR1	3.032425202	3.54E-07	1.29E-06
ENSG00000106853	PTGR1	3.031042461	9.11E-11	5.30E-10
ENSG00000138061	CYP1B1	3.030114151	3.88E-12	2.68E-11
ENSG00000187775	DNAH17	3.026810258	1.85E-24	4.42E-23
ENSG00000147573	TRIM55	3.026297444	1.84E-08	7.97E-08
ENSG00000131480	AOC2	3.025561128	8.05E-27	2.34E-25
ENSG00000059728	MXD1	3.024155738	3.33E-21	5.87E-20
ENSG00000138821	SLC39A8	3.023301277	3.02E-18	4.01E-17
ENSG00000089472	HEPH	3.019155228	2.83E-09	1.38E-08
ENSG00000197632	SERPINB2	3.018578087	7.60E-15	7.04E-14
ENSG00000163218	PGLYRP4	3.018141189	5.99E-08	2.43E-07
ENSG00000114268	PFKFB4	3.016127226	2.60E-60	1.02E-57
ENSG00000196188	CTSE	3.015598795	3.93E-05	0.000105852
ENSG00000188643	S100A16	3.014232871	4.51E-05	0.000120217
ENSG00000188959	C9orf152	3.013431236	0.000738807	0.001609029
ENSG00000274180	NATD1	3.012808228	1.20E-27	3.73E-26
ENSG00000134812	CBLIF	3.010128232	0.000358724	0.000827663
ENSG00000280260	-	3.004136476	4.05E-06	1.27E-05
ENSG00000105509	HAS1	2.993707668	1.19E-06	4.03E-06
ENSG00000112062	MAPK14	2.992929211	2.85E-26	7.94E-25
ENSG00000109466	KLHL2	2.99124751	1.85E-20	3.06E-19
ENSG00000166147	FBN1	2.990855032	1.37E-14	1.24E-13
ENSG00000178726	THBD	2.990125281	5.85E-30	2.21E-28
ENSG00000171777	RASGRP4	2.986481294	2.78E-63	1.34E-60
ENSG00000274209	ANTXRL	2.981810957	0.000251292	0.000596615
ENSG00000164695	CHMP4C	2.978492256	2.80E-10	1.55E-09
ENSG00000076944	STXBP2	2.978292764	3.98E-35	2.19E-33
ENSG00000186204	CYP4F12	2.975938094	1.51E-14	1.35E-13
ENSG00000148483	TMEM236	2.975368732	1.62E-10	9.19E-10
ENSG00000112212	TSPO2	2.974477054	7.72E-07	2.69E-06
ENSG00000140623	SEPT12	2.972453888	1.66E-05	4.75E-05
ENSG00000112984	KIF20A	2.971561374	2.61E-12	1.83E-11
ENSG00000130208	APOC1	2.971194015	4.10E-05	0.000109875
ENSG00000168298	HIST1H1E	2.967888118	8.75E-09	3.96E-08
ENSG00000182782	HCAR2	2.965089396	8.28E-10	4.31E-09
ENSG00000096006	CRISP3	2.963778642	5.70E-07	2.02E-06
ENSG00000244734	HBB	2.961931002	2.20E-06	7.16E-06
ENSG00000137312	FLOT1	2.960520805	7.78E-45	9.79E-43

ENSG00000096060	FKBP5	2.956871838	4.63E-23	9.63E-22
ENSG00000137460	FHDC1	2.954692497	2.46E-13	1.90E-12
ENSG00000185261	KIAA0825	2.954456977	1.75E-10	9.93E-10
ENSG00000166947	EPB42	2.954164705	9.72E-10	5.01E-09
ENSG00000186806	VSIG10L	2.950829777	9.65E-22	1.76E-20
ENSG00000242550	SERPINB10	2.949214213	6.04E-11	3.59E-10
ENSG00000204389	HSPA1A	2.94879598	7.41E-34	3.73E-32
ENSG00000091106	NLRC4	2.947497599	1.06E-22	2.14E-21
ENSG00000143416	SELENBP1	2.946178855	4.81E-06	1.49E-05
ENSG00000174697	LEP	2.944528746	3.77E-05	0.000101711
ENSG00000171433	GLOD5	2.942769863	2.01E-06	6.57E-06
ENSG00000183638	RP1L1	2.941363064	9.60E-06	2.83E-05
ENSG00000203812	HIST2H2AA3	2.938166186	4.43E-12	3.02E-11
ENSG00000170909	OSCAR	2.935757659	6.85E-41	6.07E-39
ENSG00000105639	JAK3	2.934784051	2.10E-40	1.82E-38
ENSG00000166523	CLEC4E	2.934162557	9.26E-23	1.87E-21
ENSG00000010327	STAB1	2.934014494	3.59E-24	8.27E-23
ENSG00000205085	FAM71F2	2.933852968	2.68E-08	1.14E-07
ENSG00000170054	SERPINA9	2.931092207	8.06E-07	2.80E-06
ENSG00000196189	SEMA4A	2.930911862	1.12E-50	1.95E-48
ENSG00000272196	HIST2H2AA4	2.930760813	1.54E-08	6.76E-08
ENSG00000173638	SLC19A1	2.927880594	5.30E-39	4.15E-37
ENSG00000178719	GRINA	2.916380819	7.70E-44	8.80E-42
ENSG00000182118	FAM89A	2.916009318	3.18E-26	8.79E-25
ENSG00000141401	IMPA2	2.915664664	2.42E-80	3.02E-77
ENSG00000101916	TLR8	2.903780117	2.01E-13	1.57E-12
ENSG00000167014	TERB2	2.901003064	0.002370879	0.004681111
ENSG00000204010	IFIT1B	2.898468892	3.45E-06	1.09E-05
ENSG00000134243	SORT1	2.897436357	1.23E-26	3.49E-25
ENSG00000196083	IL1RAP	2.896632058	1.17E-11	7.56E-11
ENSG00000204345	CD300LD	2.894312142	9.64E-08	3.80E-07
ENSG00000197852	INKA2	2.89375612	1.24E-35	7.08E-34
ENSG00000182871	COL18A1	2.891274382	1.07E-19	1.65E-18
ENSG00000114626	ABTB1	2.889098759	2.46E-35	1.37E-33
ENSG00000147697	GSDMC	2.887931775	2.47E-05	6.86E-05
ENSG00000187837	HIST1H1C	2.885920638	7.10E-30	2.66E-28
ENSG00000079277	MKNK1	2.881089749	2.07E-39	1.68E-37
ENSG00000160410	SHKBP1	2.875506287	2.83E-28	9.25E-27
ENSG00000165029	ABCA1	2.87388121	2.61E-14	2.28E-13
ENSG00000187498	COL4A1	2.873459977	1.29E-07	5.01E-07
ENSG00000162407	PLPP3	2.873251348	2.09E-13	1.63E-12
ENSG00000117399	CDC20	2.872665086	4.36E-19	6.26E-18
ENSG00000176046	NUPR1	2.87129915	2.34E-07	8.74E-07
ENSG00000204388	HSPA1B	2.868527151	5.11E-37	3.36E-35
ENSG00000183722	LHFPL6	2.867172495	6.30E-13	4.67E-12

ENSG00000152380	FAM151B	2.865965657	1.28E-28	4.35E-27
ENSG00000136869	TLR4	2.859425301	5.58E-13	4.16E-12
ENSG00000162881	OXER1	2.85357824	3.28E-30	1.26E-28
ENSG00000024526	DEPDC1	2.850754102	1.39E-10	7.95E-10
ENSG00000064547	LPAR2	2.847236779	4.68E-25	1.17E-23
ENSG00000158402	CDC25C	2.843348897	3.08E-11	1.90E-10
ENSG00000183010	PYCR1	2.843334358	6.40E-08	2.59E-07
ENSG00000089685	BIRC5	2.839424627	2.81E-18	3.75E-17
ENSG00000157557	ETS2	2.837860684	1.10E-19	1.68E-18
ENSG00000176973	FAM89B	2.837851752	7.45E-10	3.90E-09
ENSG00000163694	RBM47	2.828003169	5.15E-35	2.79E-33
ENSG00000240583	AQP1	2.825533856	7.95E-09	3.62E-08
ENSG00000128512	DOCK4	2.820216094	2.79E-21	4.94E-20
ENSG00000132589	FLOT2	2.818496437	1.05E-33	5.28E-32
ENSG00000078053	AMPH	2.817895512	4.68E-17	5.58E-16
ENSG00000138180	CEP55	2.817283075	4.71E-14	3.97E-13
ENSG00000222009	BTBD19	2.816450122	1.08E-19	1.67E-18
ENSG00000229183	PGA4	2.816339429	0.000562119	0.001250323
ENSG00000050767	COL23A1	2.813415413	5.71E-17	6.75E-16
ENSG00000196878	LAMB3	2.81338125	4.14E-17	4.95E-16
ENSG00000100290	BIK	2.811509618	8.46E-32	3.69E-30
ENSG00000168268	NT5DC2	2.808154833	3.92E-25	9.85E-24
ENSG00000224689	ZNF812P	2.806974586	0.00126409	0.002627861
ENSG00000100228	RAB36	2.806847997	5.32E-19	7.63E-18
ENSG00000236444	UBE2L5	2.799753917	8.51E-09	3.86E-08
ENSG00000074803	SLC12A1	2.792303564	0.000177302	0.000431484
ENSG00000167995	BEST1	2.78882897	1.19E-35	6.79E-34
ENSG00000005381	MPO	2.788503347	6.66E-09	3.08E-08
ENSG00000185559	DLK1	2.787328609	0.005984993	0.010963944
ENSG00000123643	SLC36A1	2.781175652	3.77E-27	1.12E-25
ENSG00000075399	VPS9D1	2.780798038	7.72E-20	1.20E-18
ENSG00000069535	MAOB	2.779819897	1.08E-09	5.56E-09
ENSG00000168306	ACOX2	2.778938457	3.78E-15	3.65E-14
ENSG00000198018	ENTPD7	2.777285478	2.11E-15	2.09E-14
ENSG00000066336	SPI1	2.776703776	7.13E-48	1.04E-45
ENSG00000163251	FZD5	2.769647365	9.87E-25	2.39E-23
ENSG00000116260	QSOX1	2.768983394	3.85E-51	6.94E-49
ENSG00000143556	S100A7	2.768613177	0.020393196	0.033413691
ENSG00000168309	FAM107A	2.768341838	7.10E-05	0.000183291
ENSG00000184292	TACSTD2	2.768221571	1.80E-07	6.83E-07
ENSG00000064932	SBNO2	2.767769603	4.04E-44	4.80E-42
ENSG00000104055	TGM5	2.765938272	1.26E-05	3.65E-05
ENSG00000149633	KIAA1755	2.764878521	0.000479551	0.001080958
ENSG00000125744	RTN2	2.764793574	2.53E-23	5.39E-22
ENSG00000170955	CAVIN3	2.761143424	2.99E-09	1.45E-08

ENSG00000165886	UBTD1	2.755669225	5.85E-31	2.38E-29
ENSG00000128011	LRFN1	2.755292498	1.35E-27	4.19E-26
ENSG00000250361	GYPB	2.753392009	0.003188292	0.006160192
ENSG00000161940	BCL6B	2.749056803	0.00010582	0.000266042
ENSG00000111886	GABRR2	2.748622728	1.79E-09	8.95E-09
ENSG00000096063	SRPK1	2.747354963	1.48E-15	1.49E-14
ENSG00000072682	P4HA2	2.746467257	6.20E-15	5.79E-14
ENSG00000129295	LRRC6	2.742101665	6.60E-17	7.68E-16
ENSG00000261652	C15orf65	2.741504572	3.96E-11	2.42E-10
ENSG00000163191	S100A11	2.741186589	1.72E-32	7.86E-31
ENSG00000132259	CNGA4	2.739423691	4.93E-15	4.68E-14
ENSG00000182541	LIMK2	2.737730143	1.07E-18	1.49E-17
ENSG00000242221	PSG2	2.732829987	0.00109561	0.002308382
ENSG00000090376	IRAK3	2.731574254	1.41E-20	2.37E-19
ENSG00000198816	ZNF358	2.727462825	1.13E-07	4.43E-07
ENSG00000142185	TRPM2	2.727385187	7.95E-42	7.73E-40
ENSG00000136040	PLXNC1	2.726942337	6.91E-15	6.43E-14
ENSG00000035664	DAPK2	2.726123987	6.52E-22	1.20E-20
ENSG00000138075	ABCG5	2.726023442	0.000191744	0.000464141
ENSG00000184371	CSF1	2.721626081	4.54E-15	4.33E-14
ENSG00000161944	ASGR2	2.718073458	6.85E-18	8.83E-17
ENSG00000185499	MUC1	2.717373125	1.12E-27	3.50E-26
ENSG00000222047	C10orf55	2.716706593	0.002956094	0.005742676
ENSG00000101425	BPI	2.715972261	3.08E-09	1.49E-08
ENSG00000176076	KCNE5	2.712913406	3.83E-08	1.60E-07
ENSG00000150337	FCGR1A	2.711247636	6.52E-20	1.03E-18
ENSG00000120903	CHRNA2	2.711019645	5.58E-13	4.16E-12
ENSG00000105339	DENND3	2.710921961	6.53E-31	2.62E-29
ENSG00000198858	R3HDM4	2.709682721	1.14E-34	6.05E-33
ENSG00000167653	PSCA	2.707504645	0.000608274	0.001346384
ENSG00000173559	NABP1	2.705601293	8.57E-22	1.57E-20
ENSG00000132164	SLC6A11	2.704792898	0.000155269	0.000381085
ENSG00000187554	TLR5	2.701863806	2.01E-18	2.71E-17
ENSG00000146700	SSC4D	2.70075677	3.76E-13	2.86E-12
ENSG00000054793	ATP9A	2.698857239	5.72E-15	5.36E-14
ENSG00000198804	MT-CO1	2.698193821	6.68E-05	0.000173357
ENSG00000108405	P2RX1	2.697154116	1.32E-56	3.96E-54
ENSG00000103855	CD276	2.696355113	1.81E-06	5.97E-06
ENSG00000180596	HIST1H2BC	2.694641058	2.39E-15	2.36E-14
ENSG00000143226	FCGR2A	2.694550324	1.65E-20	2.74E-19
ENSG00000160161	CILP2	2.693303928	1.39E-06	4.66E-06
ENSG00000167992	VWCE	2.691995887	5.46E-08	2.23E-07
ENSG00000115386	REG1A	2.686671969	0.015493949	0.026044723
ENSG00000129038	LOXL1	2.686587146	5.06E-18	6.61E-17
ENSG00000117525	F3	2.686456872	3.27E-08	1.38E-07

ENSG00000165805	C12orf50	2.685689026	0.000531467	0.001187788
ENSG00000181751	C5orf30	2.683584522	2.79E-14	2.42E-13
ENSG00000177464	GPR4	2.683418816	1.14E-05	3.34E-05
ENSG00000158445	KCNB1	2.68227987	8.16E-10	4.25E-09
ENSG00000137474	MYO7A	2.681908655	1.60E-23	3.51E-22
ENSG00000136689	IL1RN	2.68076983	3.57E-18	4.73E-17
ENSG00000175445	LPL	2.677998031	2.47E-11	1.54E-10
ENSG00000198886	MT-ND4	2.676487913	1.00E-04	0.000252309
ENSG00000165030	NFIL3	2.676436636	2.14E-12	1.51E-11
ENSG00000227268	KLLN	2.675950846	4.79E-16	5.08E-15
ENSG00000021852	C8B	2.673936379	0.000278609	0.000656576
ENSG00000100583	SAMD15	2.672541486	1.12E-09	5.71E-09
ENSG00000198844	ARHGEF15	2.669497503	0.001109386	0.002333794
ENSG00000212907	MT-ND4L	2.668597945	1.66E-06	5.50E-06
ENSG00000189077	TMEM120A	2.667139674	8.14E-39	6.22E-37
ENSG00000134668	SPOCD1	2.666661382	9.04E-13	6.63E-12
ENSG00000143344	RGL1	2.66611222	2.61E-17	3.20E-16
ENSG00000104903	LYL1	2.662615353	2.98E-18	3.96E-17
ENSG00000198223	CSF2RA	2.66200893	9.44E-36	5.48E-34
ENSG00000085063	CD59	2.661638086	8.72E-20	1.35E-18
ENSG00000021355	SERPINB1	2.660739822	3.89E-30	1.48E-28
ENSG00000019991	HGF	2.660063605	6.11E-13	4.54E-12
ENSG00000140563	MCTP2	2.659647438	4.12E-19	5.95E-18
ENSG00000155307	SAMSN1	2.651921088	9.25E-13	6.76E-12
ENSG00000172232	AZU1	2.647634023	2.63E-06	8.47E-06
ENSG00000127412	TRPV5	2.644158095	8.85E-06	2.63E-05
ENSG00000196747	HIST1H2AI	2.643158309	2.24E-10	1.26E-09
ENSG00000244242	IFITM10	2.642017478	1.52E-13	1.20E-12
ENSG00000128165	ADM2	2.640421568	5.58E-13	4.16E-12
ENSG00000070019	GUCY2C	2.639989796	3.69E-16	3.95E-15
ENSG00000185056	C5orf47	2.639140963	3.34E-05	9.09E-05
ENSG00000006606	CCL26	2.638562558	0.000801745	0.001734122
ENSG00000198938	MT-CO3	2.636421713	2.02E-05	5.67E-05
ENSG00000171860	C3AR1	2.635086677	9.23E-16	9.53E-15
ENSG00000121316	PLBD1	2.634731343	1.35E-22	2.69E-21
ENSG00000025039	RRAGD	2.634634899	3.54E-29	1.25E-27
ENSG00000100031	GGT1	2.631565528	1.18E-43	1.33E-41
ENSG00000171786	NHLH1	2.628678872	1.79E-15	1.79E-14
ENSG00000135404	CD63	2.627037111	6.34E-35	3.40E-33
ENSG00000161640	SIGLEC11	2.626861094	3.57E-11	2.18E-10
ENSG00000125845	BMP2	2.62437162	9.82E-07	3.38E-06
ENSG00000188483	IER5L	2.621369381	3.07E-10	1.68E-09
ENSG00000077238	IL4R	2.621031314	2.62E-27	7.86E-26
ENSG00000135838	NPL	2.620285351	5.16E-28	1.66E-26
ENSG00000101057	MYBL2	2.619220168	6.09E-14	5.09E-13

ENSG00000180089	TMEM86B	2.619118467	1.51E-10	8.61E-10
ENSG00000054523	KIF1B	2.611906687	2.05E-19	3.08E-18
ENSG00000136383	ALPK3	2.610157205	2.05E-10	1.15E-09
ENSG00000137270	GCM1	2.61010368	8.31E-08	3.31E-07
ENSG00000182492	BGN	2.608486149	0.000249641	0.000592978
ENSG00000137801	THBS1	2.607617387	1.87E-09	9.32E-09
ENSG00000087157	PGS1	2.603922092	2.26E-24	5.32E-23
ENSG00000148180	GSN	2.603002919	4.70E-23	9.75E-22
ENSG00000169180	XPO6	2.602557288	4.44E-22	8.37E-21
ENSG00000168528	SERINC2	2.602203673	1.97E-09	9.80E-09
ENSG00000135821	GLUL	2.600263939	1.45E-31	6.18E-30
ENSG00000174007	CEP19	2.596595246	4.70E-15	4.47E-14
ENSG00000185339	TCN2	2.595380942	2.90E-18	3.86E-17
ENSG00000140678	ITGAX	2.595361215	5.75E-31	2.35E-29
ENSG00000282608	ADORA3	2.590378517	2.88E-16	3.13E-15
ENSG00000169896	ITGAM	2.590325483	1.23E-33	6.10E-32
ENSG00000161888	SPC24	2.5862898	1.37E-10	7.86E-10
ENSG00000089505	CMTM1	2.580597118	4.19E-11	2.54E-10
ENSG00000183668	PSG9	2.579841123	0.015435428	0.025952178
ENSG00000100368	CSF2RB	2.578669585	1.06E-13	8.56E-13
ENSG00000198899	MT-ATP6	2.575763015	4.63E-05	0.000123337
ENSG00000170017	ALCAM	2.574140691	2.01E-19	3.02E-18
ENSG00000170476	MZB1	2.571119651	2.63E-09	1.28E-08
ENSG00000110203	FOLR3	2.570763484	3.93E-06	1.23E-05
ENSG00000170323	FABP4	2.570117467	3.16E-05	8.62E-05
ENSG00000198604	BAZ1A	2.569622867	7.51E-23	1.54E-21
ENSG00000197405	C5AR1	2.569598969	1.10E-18	1.53E-17
ENSG00000174125	TLR1	2.569440606	9.11E-14	7.45E-13
ENSG00000160999	SH2B2	2.568857016	1.93E-20	3.18E-19
ENSG00000181631	P2RY13	2.563967403	4.70E-23	9.75E-22
ENSG00000123485	HJURP	2.563900645	1.71E-13	1.35E-12
ENSG00000100979	PLTP	2.563282528	4.09E-22	7.75E-21
ENSG00000158373	HIST1H2BD	2.558784077	3.10E-19	4.55E-18
ENSG00000121060	TRIM25	2.557866963	6.74E-26	1.81E-24
ENSG00000184557	SOCS3	2.557699815	4.85E-22	9.10E-21
ENSG00000257704	INAFM1	2.557054151	1.54E-12	1.10E-11
ENSG00000005961	ITGA2B	2.556992001	4.92E-11	2.96E-10
ENSG00000134830	C5AR2	2.556041835	1.83E-36	1.14E-34
ENSG00000205403	CFI	2.553738195	9.48E-05	0.000240422
ENSG00000142552	RCN3	2.549283841	1.58E-21	2.85E-20
ENSG00000198888	MT-ND1	2.548522215	6.78E-05	0.000175978
ENSG00000107831	FGF8	2.548305575	0.00017262	0.000420774
ENSG00000166033	HTRA1	2.547357386	3.63E-08	1.52E-07
ENSG00000260916	CCPG1	2.547025153	3.74E-14	3.20E-13
ENSG00000158517	NCF1	2.543757436	3.46E-25	8.75E-24

ENSG00000187695	-	2.543546794	2.39E-06	7.72E-06
ENSG00000254470	AP5B1	2.542030897	1.19E-25	3.14E-24
ENSG00000188037	CLCN1	2.540111574	3.50E-09	1.68E-08
ENSG00000042062	RIPOR3	2.539770882	4.15E-15	3.98E-14
ENSG00000105697	HAMP	2.538146891	3.46E-08	1.45E-07
ENSG00000181029	TRAPPC5	2.535353405	3.09E-16	3.35E-15
ENSG00000170832	USP32	2.535324578	1.81E-16	2.01E-15
ENSG00000162032	SPSB3	2.534943245	2.50E-06	8.06E-06
ENSG00000159588	CCDC17	2.53347443	8.15E-28	2.57E-26
ENSG00000129991	TNNI3	2.532266501	0.023459066	0.037816348
ENSG00000177663	IL17RA	2.52902677	5.49E-39	4.26E-37
ENSG00000127564	PKMYT1	2.527364294	2.25E-11	1.41E-10
ENSG00000169397	RNASE3	2.526516844	1.36E-07	5.27E-07
ENSG00000158470	B4GALT5	2.522435119	7.10E-15	6.59E-14
ENSG00000148200	NR6A1	2.519854047	3.46E-29	1.23E-27
ENSG00000139178	C1RL	2.519749129	8.39E-42	8.06E-40
ENSG00000158428	CATIP	2.519659412	4.55E-25	1.14E-23
ENSG00000157335	CLEC18C	2.517934918	5.05E-05	0.000133616
ENSG00000114251	WNT5A	2.516530786	8.67E-05	0.000220923
ENSG00000152583	SPARCL1	2.516159833	9.47E-05	0.000240181
ENSG00000063015	SEZ6	2.51590735	7.82E-10	4.08E-09
ENSG00000169994	MYO7B	2.515020536	1.28E-29	4.70E-28
ENSG00000004660	CAMKK1	2.513192978	1.98E-37	1.34E-35
ENSG00000105419	MEIS3	2.512720887	1.72E-05	4.91E-05
ENSG00000005238	FAM214B	2.512659081	2.74E-55	7.31E-53
ENSG00000101336	HCK	2.510756816	7.20E-34	3.64E-32
ENSG00000198727	MT-CYB	2.508492482	0.000106462	0.000267592
ENSG00000003436	TFPI	2.507046652	3.30E-11	2.03E-10
ENSG00000163531	NFASC	2.502594708	1.70E-09	8.52E-09
ENSG00000133169	BEX1	2.50235554	4.94E-11	2.97E-10
ENSG00000166987	MBD6	2.501368131	1.86E-41	1.72E-39
ENSG00000198712	MT-CO2	2.499800683	1.36E-05	3.93E-05
ENSG00000159840	ZYX	2.499223943	5.38E-39	4.20E-37
ENSG00000253250	C8orf88	2.498251283	5.17E-07	1.84E-06
ENSG00000169403	PTAFR	2.495864843	1.58E-33	7.85E-32
ENSG00000197249	SERPINA1	2.49524225	2.78E-22	5.33E-21
ENSG00000250644	AC068580.4	2.494652347	9.26E-12	6.07E-11
ENSG00000125910	S1PR4	2.494206454	1.67E-23	3.65E-22
ENSG00000129450	SIGLEC9	2.493558572	6.23E-41	5.59E-39
ENSG00000179776	CDH5	2.493374248	2.45E-07	9.09E-07
ENSG00000117298	ECE1	2.491889758	1.44E-31	6.18E-30
ENSG00000157322	CLEC18A	2.491865667	3.25E-14	2.79E-13
ENSG00000142046	TMEM91	2.491672884	9.06E-24	2.03E-22
ENSG00000146955	RAB19	2.491138907	6.99E-06	2.11E-05
ENSG00000184792	OSBP2	2.490161965	3.73E-06	1.17E-05

ENSG00000022567	SLC45A4	2.489984894	6.39E-26	1.72E-24
ENSG00000122861	PLAU	2.486829297	1.11E-09	5.70E-09
ENSG00000100300	TSPO	2.48620486	5.21E-26	1.42E-24
ENSG00000243440	-	2.485911641	9.38E-06	2.77E-05
ENSG00000188559	RALGAPA2	2.483942379	5.79E-19	8.23E-18
ENSG00000102287	GABRE	2.48371091	0.000411527	0.000937502
ENSG00000087253	LPCAT2	2.480843744	1.51E-14	1.35E-13
ENSG00000105737	GRIK5	2.480280416	7.54E-11	4.44E-10
ENSG00000148572	NRBF2	2.478288057	8.66E-18	1.10E-16
ENSG00000086544	ITPKC	2.474668401	3.14E-38	2.29E-36
ENSG00000164362	TERT	2.474606165	9.90E-05	0.00025031
ENSG00000177628	GBA	2.474154379	1.08E-22	2.17E-21
ENSG00000177105	RHOG	2.470733241	7.82E-30	2.91E-28
ENSG00000163449	TMEM169	2.470320284	1.55E-12	1.11E-11
ENSG00000064270	ATP2C2	2.470162908	1.40E-15	1.41E-14
ENSG00000170835	CEL	2.466236249	1.55E-14	1.39E-13
ENSG00000138411	HECW2	2.462905294	1.24E-17	1.56E-16
ENSG00000107593	PKD2L1	2.457157453	5.17E-08	2.11E-07
ENSG00000148773	MKI67	2.45527479	5.70E-17	6.75E-16
ENSG00000106348	IMPDH1	2.45394987	1.08E-53	2.52E-51
ENSG00000174837	ADGRE1	2.451608679	1.49E-20	2.49E-19
ENSG00000138207	RBP4	2.449289011	5.83E-05	0.000152763
ENSG00000172216	CEBPB	2.448964513	3.27E-26	9.05E-25
ENSG00000103490	PYCARD	2.447852094	8.92E-14	7.30E-13
ENSG00000221869	CEBPD	2.446644638	1.76E-22	3.45E-21
ENSG00000140749	IGSF6	2.445356232	7.46E-20	1.17E-18
ENSG00000171657	GPR82	2.444480937	2.56E-13	1.99E-12
ENSG00000184730	APOBR	2.441018973	1.56E-36	9.86E-35
ENSG00000169429	CXCL8	2.440568239	1.27E-05	3.69E-05
ENSG00000127948	POR	2.4399473	1.41E-35	7.98E-34
ENSG00000090889	KIF4A	2.439876861	2.21E-15	2.19E-14
ENSG00000087237	CETP	2.439006642	5.51E-15	5.18E-14
ENSG00000178999	AURKB	2.43647036	1.22E-23	2.70E-22
ENSG00000185262	UBALD2	2.435721822	4.18E-20	6.73E-19
ENSG00000149798	CDC42EP2	2.435104007	4.53E-13	3.41E-12
ENSG00000058091	CDK14	2.434932518	1.87E-12	1.33E-11
ENSG00000133687	TMTC1	2.428344364	7.40E-07	2.58E-06
ENSG00000244617	ASPRV1	2.427497551	5.21E-09	2.44E-08
ENSG00000182511	FES	2.4250153	6.85E-50	1.17E-47
ENSG00000132825	PPP1R3D	2.424739171	3.61E-16	3.88E-15
ENSG00000146950	SHROOM2	2.424437421	0.003950533	0.0074975
ENSG00000184497	TMEM255B	2.42423288	2.87E-19	4.22E-18
ENSG00000089351	GRAMD1A	2.423950951	6.31E-38	4.48E-36
ENSG00000130707	ASS1	2.423926579	1.99E-05	5.58E-05
ENSG00000177685	CRACR2B	2.421870172	2.00E-15	1.99E-14

ENSG00000197272	IL27	2.421812455	2.81E-20	4.58E-19
ENSG00000175592	FOSL1	2.421289593	8.06E-10	4.20E-09
ENSG00000135862	LAMC1	2.420739772	2.25E-16	2.48E-15
ENSG00000197063	MAFG	2.419325778	2.60E-49	4.28E-47
ENSG00000126861	OMG	2.418402667	1.23E-16	1.39E-15
ENSG00000115607	IL18RAP	2.418092627	5.93E-15	5.56E-14
ENSG00000177398	UMODL1	2.41638911	9.44E-05	0.000239541
ENSG00000183570	PCBP3	2.415113292	6.77E-21	1.17E-19
ENSG00000279448	-	2.413787229	1.44E-05	4.13E-05
ENSG00000119471	HSDL2	2.412274506	5.38E-20	8.51E-19
ENSG00000157800	SLC37A3	2.410475318	1.51E-17	1.88E-16
ENSG00000121236	TRIM6	2.40883908	8.43E-09	3.83E-08
ENSG00000183784	C9orf66	2.408351778	3.20E-30	1.23E-28
ENSG00000143603	KCNN3	2.407898149	7.79E-09	3.56E-08
ENSG00000129244	ATP1B2	2.406717144	0.000183281	0.000445021
ENSG00000116016	EPAS1	2.404984163	4.89E-14	4.12E-13
ENSG00000183688	RFLNB	2.40455977	2.53E-23	5.39E-22
ENSG00000167552	TUBA1A	2.403744841	3.09E-28	1.01E-26
ENSG00000249884	RNF103-CHMP3	2.403397418	0.000148214	0.000365206
ENSG00000052749	RRP12	2.403208331	9.60E-41	8.40E-39
ENSG00000174791	RIN1	2.403023521	5.22E-09	2.44E-08
ENSG00000165949	IFI27	2.402093864	0.000152311	0.000374315
ENSG00000121297	TSHZ3	2.400993718	1.42E-16	1.59E-15
ENSG00000164251	F2RL1	2.399869741	1.78E-08	7.75E-08
ENSG00000144668	ITGA9	2.399684174	6.28E-18	8.16E-17
ENSG00000198753	PLXNB3	2.398385353	4.33E-10	2.32E-09
ENSG00000149575	SCN2B	2.394372872	0.000738859	0.001609029
ENSG00000022840	RNF10	2.392229091	5.53E-22	1.03E-20
ENSG00000068137	PLEKHH3	2.388563357	1.84E-11	1.17E-10
ENSG00000164120	HPGD	2.388289489	2.01E-10	1.13E-09
ENSG00000157227	MMP14	2.387132298	3.59E-19	5.23E-18
ENSG00000130598	TNNI2	2.386207764	5.43E-14	4.56E-13
ENSG00000110080	ST3GAL4	2.386001318	1.35E-19	2.06E-18
ENSG00000146828	SLC12A9	2.385868728	8.61E-41	7.58E-39
ENSG00000138678	GPAT3	2.385077751	2.93E-11	1.81E-10
ENSG00000102265	TIMP1	2.384354142	7.26E-18	9.32E-17
ENSG00000102554	KLF5	2.384318564	3.95E-12	2.72E-11
ENSG00000144118	RALB	2.380742782	3.20E-17	3.87E-16
ENSG00000122642	FKBP9	2.380165332	8.93E-28	2.80E-26
ENSG00000163359	COL6A3	2.37988754	2.44E-11	1.52E-10
ENSG00000164683	HEY1	2.379302658	1.51E-05	4.34E-05
ENSG00000113368	LMNB1	2.378926684	9.45E-15	8.63E-14
ENSG00000072110	ACTN1	2.37786484	1.03E-26	2.94E-25
ENSG00000172985	SH3RF3	2.375203543	1.71E-18	2.33E-17

ENSG00000161031	PGLYRP2	2.374004006	5.10E-12	3.45E-11
ENSG00000142512	SIGLEC10	2.373351895	4.67E-23	9.72E-22
ENSG00000142694	EVA1B	2.371217656	8.36E-10	4.35E-09
ENSG00000148175	STOM	2.369199575	2.58E-17	3.16E-16
ENSG00000140332	TLE3	2.369095233	7.20E-27	2.10E-25
ENSG00000129173	E2F8	2.368839811	5.77E-09	2.69E-08
ENSG00000077942	FBLN1	2.368817413	0.004527096	0.008482074
ENSG00000158887	MPZ	2.367543863	7.38E-29	2.54E-27
ENSG00000125144	MT1G	2.36735984	0.001125772	0.00236643
ENSG00000160991	ORAI2	2.367005821	6.10E-34	3.11E-32
ENSG00000014164	ZC3H3	2.366307801	1.73E-19	2.61E-18
ENSG00000011198	ABHD5	2.365380356	3.81E-17	4.57E-16
ENSG00000169692	AGPAT2	2.363937616	5.23E-26	1.42E-24
ENSG00000161544	CYGB	2.363128923	1.80E-05	5.11E-05
ENSG00000169704	GP9	2.362953361	6.94E-08	2.79E-07
ENSG00000278599	TBC1D3E	2.361957679	7.16E-10	3.75E-09
ENSG00000171847	FAM90A1	2.361774587	2.34E-11	1.47E-10
ENSG00000183621	ZNF438	2.36051143	1.03E-25	2.73E-24
ENSG00000101307	SIRPB1	2.359418484	8.85E-23	1.80E-21
ENSG00000067836	ROGDI	2.35864421	1.03E-25	2.73E-24
ENSG00000181016	LSMEM1	2.356945558	1.20E-16	1.36E-15
ENSG00000166091	CMTM5	2.356872809	1.66E-07	6.34E-07
ENSG00000132510	KDM6B	2.354176085	1.75E-23	3.81E-22
ENSG00000137757	CASP5	2.353871362	3.83E-15	3.69E-14
ENSG00000136010	ALDH1L2	2.353345011	1.16E-15	1.19E-14
ENSG00000183396	TMEM89	2.352782542	4.33E-06	1.35E-05
ENSG00000115425	PECR	2.350715521	2.70E-15	2.64E-14
ENSG00000152213	ARL11	2.346919904	4.73E-32	2.12E-30
ENSG00000163932	PRKCD	2.345280856	1.19E-43	1.33E-41
ENSG00000163563	MNDA	2.345014072	6.74E-11	3.99E-10
ENSG00000204160	ZDHHC18	2.343471705	2.97E-16	3.22E-15
ENSG00000140534	TICRR	2.342939609	4.21E-10	2.26E-09
ENSG00000120318	ARAP3	2.342316885	9.61E-36	5.55E-34
ENSG00000127507	ADGRE2	2.341656886	9.22E-21	1.57E-19
ENSG00000173421	CCDC36	2.340547353	3.20E-07	1.18E-06
ENSG00000140682	TGFB1I1	2.340089128	9.78E-08	3.85E-07
ENSG00000256713	PGA5	2.33877945	0.000144886	0.000357652
ENSG00000188404	SELL	2.337348691	3.91E-14	3.33E-13
ENSG00000165480	SKA3	2.334125213	3.81E-11	2.33E-10
ENSG00000118473	SGIP1	2.333946434	0.000130554	0.000323555
ENSG00000170892	TSEN34	2.332763971	3.78E-33	1.82E-31
ENSG00000105355	PLIN3	2.332026167	2.56E-24	6.00E-23
ENSG00000186642	PDE2A	2.331086672	1.03E-08	4.60E-08
ENSG00000158874	APOA2	2.329129222	4.00E-08	1.67E-07
ENSG00000178217	SH2D4B	2.328604935	2.09E-09	1.04E-08

ENSG00000107738	VSIR	2.328101386	8.03E-45	1.00E-42
ENSG00000188596	CFAP54	2.327531265	4.81E-09	2.26E-08
ENSG00000177156	TALDO1	2.326632793	8.08E-30	2.99E-28
ENSG00000158792	SPATA2L	2.326284592	2.68E-09	1.31E-08
ENSG00000172638	EFEMP2	2.325482776	7.10E-20	1.11E-18
ENSG00000011426	ANLN	2.323451103	4.78E-12	3.25E-11
ENSG00000142089	IFITM3	2.322915884	1.25E-13	1.00E-12
ENSG00000133121	STARD13	2.321881841	1.14E-09	5.81E-09
ENSG00000169860	P2RY1	2.318280837	1.15E-13	9.30E-13
ENSG00000032444	PNPLA6	2.317765553	1.76E-22	3.45E-21
ENSG00000128641	MYO1B	2.317693565	3.92E-06	1.23E-05
ENSG00000177706	FAM20C	2.316444852	4.81E-22	9.03E-21
ENSG00000056998	GYG2	2.316271301	0.022107441	0.035923692
ENSG00000120709	FAM53C	2.315954071	5.89E-13	4.38E-12
ENSG00000178904	DPY19L3	2.31550866	2.43E-15	2.39E-14
ENSG00000147862	NFIB	2.314486529	6.05E-06	1.84E-05
ENSG00000100505	TRIM9	2.313916782	1.89E-06	6.19E-06
ENSG00000033627	ATP6V0A1	2.313388022	3.90E-46	5.12E-44
ENSG00000138722	MMRN1	2.312429462	2.08E-10	1.17E-09
ENSG00000101265	RASSF2	2.310146886	6.70E-24	1.52E-22
ENSG00000187068	C3orf70	2.309672114	0.002351911	0.00464611
ENSG00000180767	CHST13	2.309656567	9.73E-15	8.87E-14
ENSG00000160746	ANO10	2.306431503	6.77E-21	1.17E-19
ENSG00000074181	NOTCH3	2.306158725	1.14E-16	1.30E-15
ENSG00000163081	CCDC140	2.304945572	0.000384707	0.00088258
ENSG00000095739	BAMBI	2.30203858	1.58E-12	1.13E-11
ENSG00000130382	MLLT1	2.299917477	5.78E-44	6.71E-42
ENSG00000161533	ACOX1	2.295585393	1.66E-22	3.27E-21
ENSG00000158055	GRHL3	2.295337541	6.53E-05	0.000169717
ENSG00000130876	SLC7A10	2.29431832	0.000306857	0.000716047
ENSG00000144476	ACKR3	2.293658382	5.22E-08	2.14E-07
ENSG00000183696	UPP1	2.291432411	6.18E-29	2.14E-27
ENSG00000105707	HPN	2.289927093	1.01E-08	4.54E-08
ENSG00000137462	TLR2	2.289833553	4.26E-20	6.83E-19
ENSG00000188393	CLEC2A	2.289036859	0.006494604	0.011810809
ENSG00000143479	DYRK3	2.28898705	9.20E-11	5.35E-10
ENSG00000136048	DRAM1	2.285923116	1.14E-25	3.00E-24
ENSG00000131389	SLC6A6	2.285052769	3.13E-14	2.70E-13
ENSG00000102882	MAPK3	2.281536983	7.68E-32	3.37E-30
ENSG00000113361	CDH6	2.274063783	0.000680391	0.001492989
ENSG00000109743	BST1	2.267619648	1.44E-14	1.30E-13
ENSG00000177674	AGTRAP	2.265021234	1.66E-28	5.54E-27
ENSG00000137575	SDCBP	2.264729588	2.30E-10	1.28E-09
ENSG00000220008	LINGO3	2.264386784	1.28E-23	2.83E-22
ENSG00000172426	RSPH9	2.26299346	8.36E-15	7.68E-14

ENSG00000117834	SLC5A9	2.258318701	2.22E-17	2.73E-16
ENSG00000125900	SIRPD	2.25806112	3.96E-20	6.39E-19
ENSG00000217442	SYCE3	2.257218264	0.00020167	0.000486359
ENSG00000183111	ARHGEF37	2.256824688	4.54E-06	1.41E-05
ENSG00000148450	MSRB2	2.253438698	8.96E-22	1.64E-20
ENSG00000131759	RARA	2.252115842	4.24E-42	4.17E-40
ENSG00000197321	SVIL	2.250232253	1.27E-17	1.60E-16
ENSG00000151715	TMEM45B	2.249224595	3.10E-11	1.91E-10
ENSG00000171136	RLN3	2.248921888	9.12E-07	3.15E-06
ENSG00000024422	EHD2	2.248604003	1.09E-07	4.25E-07
ENSG00000167566	NCKAP5L	2.248151508	2.56E-34	1.33E-32
ENSG00000108106	UBE2S	2.246358063	4.68E-11	2.83E-10
ENSG00000157456	CCNB2	2.242643273	3.12E-16	3.37E-15
ENSG00000164047	CAMP	2.242457507	6.67E-05	0.000173189
ENSG00000196352	CD55	2.242051757	6.31E-16	6.60E-15
ENSG00000167414	GNG8	2.241914659	8.77E-06	2.61E-05
ENSG00000109193	SULT1E1	2.240945574	0.02319428	0.037458061
ENSG00000118193	KIF14	2.240905832	9.51E-13	6.95E-12
ENSG00000101412	E2F1	2.239869742	9.93E-11	5.74E-10
ENSG00000102145	GATA1	2.238417455	5.01E-08	2.06E-07
ENSG00000125753	VASP	2.237499631	1.28E-35	7.26E-34
ENSG00000143409	MINDY1	2.237034595	4.25E-30	1.62E-28
ENSG00000116729	WLS	2.236552267	2.37E-08	1.01E-07
ENSG00000154529	CNTNAP3B	2.235539689	3.39E-05	9.23E-05
ENSG00000103196	CRISPLD2	2.234573514	7.75E-13	5.72E-12
ENSG00000167536	DHRS13	2.232568851	3.76E-12	2.60E-11
ENSG00000072571	HMMR	2.23234563	3.50E-11	2.15E-10
ENSG00000133318	RTN3	2.232259545	1.88E-18	2.55E-17
ENSG00000157554	ERG	2.231270109	1.09E-09	5.58E-09
ENSG00000163145	C1QTNF7	2.230271412	0.003258574	0.006287062
ENSG00000133246	PRAM1	2.228280372	2.31E-28	7.57E-27
ENSG00000164626	KCNK5	2.228203529	4.81E-11	2.90E-10
ENSG00000212864	RNF208	2.227041541	2.26E-07	8.46E-07
ENSG00000074276	CDHR2	2.225486114	2.14E-11	1.35E-10
ENSG00000154589	LY96	2.217457586	6.16E-14	5.14E-13
ENSG00000260861	AL049634.2	2.215471313	3.14E-07	1.15E-06
ENSG00000128383	APOBEC3A	2.212994424	2.69E-12	1.89E-11
ENSG00000152229	PSTPIP2	2.208843033	3.80E-21	6.69E-20
ENSG00000147459	DOCK5	2.208110835	6.34E-18	8.22E-17
ENSG00000157388	CACNA1D	2.207976659	6.30E-10	3.32E-09
ENSG00000182676	PPP1R27	2.207341423	1.04E-06	3.57E-06
ENSG00000197238	HIST1H4J	2.205325508	3.30E-05	9.00E-05
ENSG00000070371	CLTCL1	2.203894074	1.96E-25	5.09E-24
ENSG00000075624	ACTB	2.203852187	3.70E-43	4.01E-41
ENSG00000063245	EPN1	2.203359727	6.81E-18	8.79E-17

ENSG00000084731	KIF3C	2.202948605	9.21E-23	1.87E-21
ENSG00000039987	BEST2	2.201074314	1.42E-06	4.75E-06
ENSG00000239732	TLR9	2.199425634	1.17E-05	3.41E-05
ENSG00000130830	MPP1	2.19940272	1.08E-30	4.27E-29
ENSG00000108582	CPD	2.198858985	3.10E-10	1.70E-09
ENSG00000133805	AMPD3	2.198721693	7.70E-22	1.42E-20
ENSG00000070182	SPTB	2.197228413	1.47E-07	5.65E-07
ENSG00000160796	NBEAL2	2.195596414	4.04E-33	1.93E-31
ENSG00000184260	HIST2H2AC	2.194318613	2.25E-13	1.75E-12
ENSG00000145287	PLAC8	2.192972348	6.87E-12	4.58E-11
ENSG00000106772	PRUNE2	2.192308771	2.67E-14	2.33E-13
ENSG00000158869	FCER1G	2.19151646	7.52E-22	1.39E-20
ENSG00000146670	CDCA5	2.187747934	8.38E-12	5.53E-11
ENSG00000260325	HSPB9	2.187124332	9.70E-08	3.82E-07
ENSG00000125735	TNFSF14	2.185662064	1.94E-13	1.52E-12
ENSG00000116691	MIIP	2.185194897	2.17E-17	2.68E-16
ENSG00000172940	SLC22A13	2.184518173	8.72E-05	0.000222204
ENSG00000100558	PLEK2	2.181741778	0.000169702	0.000414269
ENSG00000248483	POU5F2	2.180824739	2.46E-07	9.12E-07
ENSG00000135596	MICAL1	2.180748047	1.38E-37	9.46E-36
ENSG00000007001	UPP2	2.179645757	0.000113097	0.000283103
ENSG00000187244	BCAM	2.179645613	0.000554295	0.001234202
ENSG00000145428	RNF175	2.177781536	4.72E-29	1.66E-27
ENSG00000090020	SLC9A1	2.177020505	2.48E-32	1.12E-30
ENSG00000135476	ESPL1	2.175561425	4.30E-11	2.61E-10
ENSG00000145431	PDGFC	2.175071278	2.35E-12	1.66E-11
ENSG00000111199	TRPV4	2.174909864	4.36E-08	1.80E-07
ENSG00000198833	UBE2J1	2.174474109	4.64E-22	8.73E-21
ENSG00000138315	OIT3	2.16996465	0.000155917	0.000382613
ENSG00000165879	FRAT1	2.169278184	2.11E-27	6.41E-26
ENSG00000160211	G6PD	2.168849415	4.16E-38	3.00E-36
ENSG00000204531	POU5F1	2.16773283	5.14E-09	2.41E-08
ENSG00000243646	IL10RB	2.167186788	2.03E-30	7.91E-29
ENSG00000155926	SLA	2.166756848	4.83E-17	5.75E-16
ENSG00000153976	HS3ST3A1	2.165925327	0.004334223	0.008154373
ENSG00000165490	DDIAS	2.163733198	1.50E-09	7.56E-09
ENSG00000214212	C19orf38	2.160733271	1.89E-25	4.91E-24
ENSG00000196935	SRGAP1	2.160710819	1.15E-11	7.47E-11
ENSG00000174175	SELP	2.159362422	2.67E-11	1.66E-10
ENSG00000158352	SHROOM4	2.157501044	1.74E-13	1.37E-12
ENSG00000254415	SIGLEC14	2.157183419	0.01291786	0.02214448
ENSG00000185950	IRS2	2.15554009	4.80E-14	4.05E-13
ENSG00000113742	CPEB4	2.151516701	9.26E-09	4.19E-08
ENSG00000143641	GALNT2	2.150902285	6.35E-19	8.97E-18
ENSG00000243449	C4orf48	2.150867751	1.07E-08	4.80E-08

ENSG00000163638	ADAMTS9	2.149303903	0.001293457	0.002685183
ENSG00000185340	GAS2L1	2.148512604	3.90E-08	1.63E-07
ENSG00000140545	MFGE8	2.14841171	2.00E-09	9.94E-09
ENSG00000276966	HIST1H4E	2.147929092	4.30E-09	2.04E-08
ENSG00000178404	CEP295NL	2.147872584	5.58E-10	2.96E-09
ENSG00000140564	FURIN	2.146779683	9.06E-33	4.23E-31
ENSG00000110719	TCIRG1	2.146464038	2.45E-27	7.39E-26
ENSG00000119686	FLVCR2	2.145531559	2.00E-24	4.77E-23
ENSG00000124469	CEACAM8	2.144870686	0.00029277	0.000685956
ENSG00000136810	TXN	2.144331832	3.52E-21	6.20E-20
ENSG00000077420	APBB1IP	2.143475596	1.28E-23	2.82E-22
ENSG00000127578	WFIKKN1	2.142785855	3.51E-09	1.69E-08
ENSG00000160588	MPZL3	2.142444732	4.48E-14	3.79E-13
ENSG00000172354	GNB2	2.141886177	1.28E-38	9.57E-37
ENSG00000170421	KRT8	2.140258514	3.63E-07	1.32E-06
ENSG00000131831	RAI2	2.139341557	1.87E-06	6.14E-06
ENSG00000105514	RAB3D	2.136058474	9.01E-14	7.37E-13
ENSG00000235568	NFAM1	2.135307037	1.98E-23	4.27E-22
ENSG00000152078	TMEM56	2.13322582	1.53E-05	4.39E-05
ENSG00000152661	GJA1	2.131275159	0.008528571	0.015170685
ENSG00000007237	GAS7	2.130689703	7.61E-27	2.21E-25
ENSG00000188786	MTF1	2.129458185	5.81E-17	6.86E-16
ENSG00000006652	IFRD1	2.128978441	1.37E-13	1.09E-12
ENSG00000197324	LRP10	2.127485891	9.14E-20	1.41E-18
ENSG00000179869	ABCA13	2.127354083	3.93E-05	0.000105936
ENSG00000167900	TK1	2.126111852	1.54E-16	1.72E-15
ENSG00000108352	RAPGEFL1	2.125977972	1.49E-22	2.96E-21
ENSG00000173744	AGFG1	2.125903643	1.43E-15	1.45E-14
ENSG00000217555	CKLF	2.125896404	3.94E-06	1.23E-05
ENSG00000204099	NEU4	2.122579829	0.000879906	0.001890847
ENSG00000274290	HIST1H2BE	2.122119034	1.66E-09	8.31E-09
ENSG00000103005	USB1	2.121824983	1.04E-27	3.24E-26
ENSG00000177169	ULK1	2.121319858	2.02E-30	7.91E-29
ENSG00000118263	KLF7	2.120360545	5.39E-12	3.63E-11
ENSG00000064763	FAR2	2.114122149	3.03E-20	4.93E-19
ENSG00000163734	CXCL3	2.113290279	2.40E-06	7.75E-06
ENSG00000174130	TLR6	2.112740206	2.82E-14	2.44E-13
ENSG00000137642	SORL1	2.111579371	1.48E-08	6.49E-08
ENSG00000102057	KCND1	2.111222179	7.73E-26	2.07E-24
ENSG00000144040	SFXN5	2.109881321	9.01E-56	2.55E-53
ENSG00000140044	JDP2	2.108098293	6.10E-27	1.79E-25
ENSG00000165507	DEPP1	2.107375068	1.08E-08	4.85E-08
ENSG00000125510	OPRL1	2.106634405	8.24E-31	3.26E-29
ENSG00000112096	SOD2	2.104843201	9.76E-11	5.66E-10
ENSG00000250305	TRMT9B	2.104550921	3.82E-14	3.26E-13

ENSG00000167613	LAIR1	2.102923082	2.47E-31	1.04E-29
ENSG00000175221	MED16	2.101658865	8.22E-17	9.49E-16
ENSG00000164104	HMGB2	2.099906289	6.93E-21	1.19E-19
ENSG00000164877	MICALL2	2.098695525	6.68E-10	3.52E-09
ENSG00000166793	YPEL4	2.098674846	1.38E-05	3.99E-05
ENSG00000069399	BCL3	2.097860189	7.55E-15	7.00E-14
ENSG00000042493	CAPG	2.09777267	1.44E-22	2.86E-21
ENSG00000148288	GBGT1	2.097561737	1.66E-27	5.09E-26
ENSG00000138190	EXOC6	2.096783787	3.05E-14	2.63E-13
ENSG00000085741	WNT11	2.095052708	4.81E-07	1.72E-06
ENSG00000008130	NADK	2.093910236	2.18E-28	7.20E-27
ENSG00000067182	TNFRSF1A	2.0911735	5.46E-30	2.06E-28
ENSG00000197140	ADAM32	2.091022742	2.25E-07	8.41E-07
ENSG00000006327	TNFRSF12A	2.090460993	1.15E-18	1.58E-17
ENSG00000129749	CHRNA10	2.090158761	4.16E-11	2.53E-10
ENSG00000171443	ZNF524	2.088869911	1.51E-12	1.08E-11
ENSG00000171564	FGB	2.088853225	0.013019054	0.022292432
ENSG00000181458	TMEM45A	2.088738931	3.11E-09	1.50E-08
ENSG00000106113	CRHR2	2.088674082	0.000249167	0.000592039
ENSG00000143369	ECM1	2.087952074	8.56E-14	7.03E-13
ENSG00000183018	AC118754.1	2.087531094	7.21E-17	8.37E-16
ENSG00000161036	LRWD1	2.087274758	2.22E-17	2.73E-16
ENSG00000197818	SLC9A8	2.086429248	1.90E-23	4.12E-22
ENSG00000160781	PAQR6	2.085647617	1.12E-18	1.54E-17
ENSG00000185033	SEMA4B	2.083993821	5.45E-52	1.03E-49
ENSG00000271605	MILR1	2.083112118	1.03E-23	2.29E-22
ENSG00000152932	RAB3C	2.083038498	2.56E-08	1.09E-07
ENSG00000164056	SPRY1	2.082095518	9.86E-07	3.38E-06
ENSG00000077327	SPAG6	2.080178224	4.51E-06	1.40E-05
ENSG00000159128	IFNGR2	2.078958326	3.16E-31	1.31E-29
ENSG00000052344	PRSS8	2.078566595	0.000386984	0.000887261
ENSG00000167513	CDT1	2.078452967	3.64E-09	1.74E-08
ENSG00000081041	CXCL2	2.077811483	0.000104263	0.00026248
ENSG00000171695	LKAAEAR1	2.073717287	0.012452132	0.021424593
ENSG00000163162	RNF149	2.073242616	4.81E-19	6.90E-18
ENSG00000180537	RNF182	2.072433721	0.002491646	0.004897317
ENSG00000140443	IGF1R	2.071625211	3.29E-11	2.03E-10
ENSG00000159423	ALDH4A1	2.070994654	5.90E-20	9.32E-19
ENSG00000078399	HOXA9	2.070655312	6.05E-13	4.50E-12
ENSG00000078902	TOLLIP	2.070120586	7.29E-58	2.23E-55
ENSG00000104805	NUCB1	2.069661497	3.10E-24	7.23E-23
ENSG00000183323	CCDC125	2.067978433	2.21E-22	4.28E-21
ENSG00000168509	HJV	2.066806458	0.006113664	0.011177779
ENSG00000133961	NUMB	2.066766526	1.58E-15	1.59E-14
ENSG00000160888	IER2	2.06563425	3.12E-09	1.50E-08

ENSG00000042832	TG	2.064368255	8.01E-08	3.20E-07
ENSG00000116701	NCF2	2.063712146	5.93E-18	7.72E-17
ENSG00000058799	YIPF1	2.062753259	3.94E-28	1.27E-26
ENSG00000253276	CCDC71L	2.062192098	2.43E-18	3.26E-17
ENSG00000165124	SVEP1	2.061978429	0.000908534	0.001945708
ENSG00000187997	C17orf99	2.061724656	0.010531245	0.018392217
ENSG00000198113	TOR4A	2.060495202	2.42E-49	4.03E-47
ENSG00000166278	C2	2.059404526	1.62E-06	5.37E-06
ENSG00000182022	CHST15	2.059207125	3.38E-18	4.48E-17
ENSG00000138356	AOX1	2.058967038	0.003368949	0.006485811
ENSG00000170190	SLC16A5	2.058340699	5.84E-38	4.17E-36
ENSG00000000938	FGR	2.057885853	9.13E-42	8.65E-40
ENSG00000086730	LAT2	2.056986348	1.81E-30	7.11E-29
ENSG00000130772	MED18	2.054825777	3.15E-25	7.98E-24
ENSG00000126264	HCST	2.053674626	1.24E-05	3.62E-05
ENSG00000279229	-	2.053167848	0.005656124	0.010413722
ENSG00000183844	FAM3B	2.053133168	0.005367508	0.009933637
ENSG00000168615	ADAM9	2.049938975	2.98E-10	1.64E-09
ENSG00000104826	LHB	2.048984228	0.002772906	0.005411377
ENSG00000131042	LILRB2	2.047867382	7.40E-28	2.35E-26
ENSG00000135503	ACVR1B	2.046006954	7.06E-25	1.73E-23
ENSG00000198763	MT-ND2	2.045232061	0.000588892	0.0013058
ENSG00000214189	ZNF788P	2.045024635	1.46E-13	1.16E-12
ENSG00000064666	CNN2	2.042463357	1.13E-24	2.72E-23
ENSG00000165757	JCAD	2.042172571	3.04E-07	1.12E-06
ENSG00000102886	GDPD3	2.04080739	7.12E-17	8.27E-16
ENSG00000185155	MIXL1	2.039043049	0.000905413	0.001940134
ENSG00000173171	MTX1	2.038622936	5.12E-23	1.06E-21
ENSG00000035862	TIMP2	2.038266937	2.12E-24	5.01E-23
ENSG00000126878	AIF1L	2.038197056	0.000504915	0.001134374
ENSG00000175352	NRIP3	2.036994278	6.80E-06	2.05E-05
ENSG00000198786	MT-ND5	2.036217426	1.74E-05	4.95E-05
ENSG00000188906	LRRK2	2.035541946	6.62E-07	2.32E-06
ENSG00000111837	MAK	2.034540582	1.45E-08	6.40E-08
ENSG00000160685	ZBTB7B	2.033135116	1.96E-67	1.18E-64
ENSG00000100266	PACSIN2	2.032834776	4.31E-23	9.00E-22
ENSG00000188229	TUBB4B	2.032776693	4.05E-21	7.09E-20
ENSG00000155097	ATP6V1C1	2.032397817	1.92E-19	2.88E-18
ENSG00000166851	PLK1	2.032386369	1.51E-14	1.35E-13
ENSG00000124145	SDC4	2.032125572	6.81E-10	3.58E-09
ENSG00000178226	PRSS36	2.031966554	1.76E-18	2.39E-17
ENSG00000198355	PIM3	2.031385195	9.75E-14	7.95E-13
ENSG00000175489	LRRC25	2.031353304	4.73E-27	1.39E-25
ENSG00000168461	RAB31	2.030482643	3.80E-15	3.67E-14
ENSG00000150967	ABCB9	2.030245828	1.45E-06	4.83E-06

ENSG00000198003	CCDC151	2.029309521	3.75E-07	1.36E-06
ENSG00000082014	SMARCD3	2.029103537	1.10E-16	1.25E-15
ENSG00000241839	PLEKHO2	2.028140766	7.86E-28	2.48E-26
ENSG00000101457	DNTTIP1	2.026430244	6.72E-32	2.96E-30
ENSG00000112561	TFEB	2.025858394	8.36E-22	1.53E-20
ENSG00000118492	ADGB	2.025347086	1.09E-05	3.20E-05
ENSG00000090238	YPEL3	2.025052927	2.53E-13	1.96E-12
ENSG00000147813	NAPRT	2.022481104	1.77E-19	2.67E-18
ENSG00000126787	DLGAP5	2.022114903	1.53E-08	6.69E-08
ENSG00000092036	HAUS4	2.016381113	6.76E-19	9.48E-18
ENSG00000109047	RCVRN	2.016250359	2.42E-10	1.35E-09
ENSG00000274750	HIST1H3E	2.014022694	1.74E-10	9.86E-10
ENSG00000177666	PNPLA2	2.013485134	6.58E-17	7.67E-16
ENSG00000204613	TRIM10	2.013145279	0.000230203	0.000550122
ENSG00000183963	SMTN	2.010416605	3.19E-10	1.74E-09
ENSG00000215114	UBXN2B	2.00978936	2.27E-10	1.27E-09
ENSG00000160785	SLC25A44	2.00952092	8.18E-25	1.99E-23
ENSG00000205126	ACCSL	2.007061908	0.003311087	0.006381802
ENSG00000184985	SORCS2	2.006819652	7.23E-12	4.80E-11
ENSG00000137767	SQOR	2.005327393	4.35E-16	4.64E-15
ENSG00000186453	FAM228A	2.003129934	2.80E-07	1.04E-06
ENSG00000150403	TMCO3	2.003093448	3.68E-16	3.95E-15
ENSG00000102109	PCSK1N	2.00031507	0.002220902	0.004404158
ENSG00000243130	PSG11	2.000051084	0.017304838	0.028791405

[a] P-value computed by DESeq2.

[b] Adjusted P-value by the Benjamini and Hochberg procedure.

Supplementary Table 4. Significantly downregulated genes of comparison between sepsis patients and healthy controls

Ensemble ID	Gene Symbol	log2FoldChange	*P*-value	*P*-value adjusted
ENSG00000165061	ZMAT4	-5.148407406	1.74E-19	2.62E-18
ENSG00000179639	FCER1A	-4.862411537	4.58E-26	1.26E-24
ENSG00000144290	SLC4A10	-4.615222294	1.33E-29	4.86E-28
ENSG00000074527	NTN4	-4.526354759	1.34E-19	2.05E-18
ENSG00000189253	TRIM64B	-4.412244719	4.05E-08	1.68E-07
ENSG00000069702	TGFBR3	-4.002479665	7.51E-55	1.94E-52
ENSG00000185697	MYBL1	-3.99806153	8.34E-63	3.90E-60
ENSG00000149534	MS4A2	-3.886758569	5.13E-20	8.14E-19
ENSG00000080493	SLC4A4	-3.830833137	1.33E-42	1.35E-40
ENSG00000255819	KLRC4-KLRK1	-3.823424679	2.58E-25	6.62E-24
ENSG00000196581	AJAP1	-3.754582478	1.05E-10	6.04E-10
ENSG00000183542	KLRC4	-3.685608135	5.31E-15	5.02E-14
ENSG00000184613	NELL2	-3.639645778	7.62E-28	2.41E-26
ENSG00000213809	KLRK1	-3.602918253	3.45E-24	7.99E-23
ENSG00000173068	BNC2	-3.576716241	7.93E-24	1.78E-22
ENSG00000150045	KLRF1	-3.520840091	5.21E-41	4.70E-39
ENSG00000156966	B3GNT7	-3.519641129	2.97E-26	8.26E-25
ENSG00000074590	NUAK1	-3.507805502	1.99E-36	1.24E-34
ENSG00000138741	TRPC3	-3.503364207	1.23E-15	1.26E-14
ENSG00000173114	LRRN3	-3.491613152	3.62E-14	3.10E-13
ENSG00000150687	PRSS23	-3.487364019	1.77E-33	8.67E-32
ENSG00000134545	KLRC1	-3.476176424	2.21E-28	7.28E-27
ENSG00000172031	EPHX4	-3.465951868	4.37E-16	4.66E-15
ENSG00000196586	MYO6	-3.431451218	1.33E-46	1.81E-44
ENSG00000113088	GZMK	-3.388392528	2.24E-21	4.00E-20
ENSG00000036448	MYOM2	-3.379444535	1.47E-08	6.47E-08
ENSG00000205810	KLRC3	-3.374881353	2.21E-11	1.39E-10
ENSG00000154269	ENPP3	-3.367854358	7.77E-18	9.95E-17
ENSG00000179841	AKAP5	-3.366670038	1.16E-52	2.39E-50
ENSG00000138311	ZNF365	-3.351133561	8.91E-32	3.88E-30
ENSG00000170624	SGCD	-3.33576776	4.82E-18	6.33E-17
ENSG00000198108	CHSY3	-3.328832166	7.31E-07	2.55E-06
ENSG00000139187	KLRG1	-3.316875251	1.48E-35	8.34E-34
ENSG00000112902	SEMA5A	-3.311773739	3.64E-17	4.38E-16
ENSG00000274588	DGKK	-3.311475773	1.78E-12	1.27E-11
ENSG00000117281	CD160	-3.306533808	3.15E-22	6.00E-21
ENSG00000186462	NAP1L2	-3.30478949	8.61E-26	2.29E-24
ENSG00000277586	NEFL	-3.302750813	3.18E-15	3.10E-14
ENSG00000069667	RORA	-3.302460339	3.99E-35	2.19E-33
ENSG00000007350	TKTL1	-3.292321633	1.57E-22	3.12E-21

ENSG00000185013	NT5C1B	-3.259957093	3.62E-08	1.52E-07
ENSG00000196368	NUDT11	-3.234657646	1.15E-15	1.18E-14
ENSG00000174482	LINGO2	-3.200080089	1.66E-15	1.66E-14
ENSG00000181036	FCRL6	-3.180325452	1.97E-31	8.35E-30
ENSG00000174348	PODN	-3.17246485	1.49E-12	1.07E-11
ENSG00000197077	KIAA1671	-3.170678308	9.50E-27	2.74E-25
ENSG00000109471	IL2	-3.169353888	1.35E-05	3.89E-05
ENSG00000162398	LEXM	-3.159655889	6.03E-31	2.44E-29
ENSG00000255582	OR10G2	-3.156084081	4.03E-08	1.68E-07
ENSG00000091129	NRCAM	-3.137302239	3.54E-12	2.45E-11
ENSG00000137501	SYTL2	-3.125586734	9.46E-43	9.71E-41
ENSG00000137959	IFI44L	-3.121564628	2.28E-18	3.06E-17
ENSG00000144824	PHLDB2	-3.120979893	5.08E-29	1.79E-27
ENSG00000185689	C6orf201	-3.118236497	1.38E-08	6.06E-08
ENSG00000111796	KLRB1	-3.103304973	4.03E-21	7.05E-20
ENSG00000183662	FAM19A1	-3.087033652	7.85E-17	9.09E-16
ENSG00000147041	SYTL5	-3.069466781	7.72E-08	3.09E-07
ENSG00000124568	SLC17A1	-3.061144547	4.57E-08	1.89E-07
ENSG00000030419	IKZF2	-3.060792038	7.15E-36	4.21E-34
ENSG00000198574	SH2D1B	-3.05917699	5.60E-21	9.70E-20
ENSG00000164483	SAMD3	-3.058119318	3.79E-40	3.26E-38
ENSG00000214652	ZNF727	-3.044851612	4.73E-06	1.46E-05
ENSG00000112796	ENPP5	-3.042068358	3.43E-24	7.94E-23
ENSG00000172673	THEMIS	-3.028168616	4.62E-23	9.63E-22
ENSG00000156463	SH3RF2	-3.00266195	1.39E-13	1.11E-12
ENSG00000163751	CPA3	-2.972245571	5.48E-07	1.95E-06
ENSG00000197057	DTHD1	-2.970490055	7.90E-24	1.78E-22
ENSG00000101230	ISM1	-2.962802677	1.65E-11	1.05E-10
ENSG00000085563	ABCB1	-2.960660106	4.29E-38	3.07E-36
ENSG00000118922	KLF12	-2.951853077	1.12E-37	7.78E-36
ENSG00000206531	CD200R1L	-2.943205414	1.56E-05	4.47E-05
ENSG00000180801	ARSJ	-2.942290161	6.17E-06	1.88E-05
ENSG00000137441	FGFBP2	-2.939905175	2.91E-12	2.03E-11
ENSG00000005001	PRSS22	-2.932244318	2.17E-09	1.07E-08
ENSG00000176928	GCNT4	-2.931534522	6.11E-26	1.65E-24
ENSG00000157985	AGAP1	-2.927274215	3.27E-21	5.79E-20
ENSG00000127377	CRYGN	-2.924431886	4.45E-08	1.84E-07
ENSG00000187135	VSTM2B	-2.920037902	2.27E-06	7.37E-06
ENSG00000186081	KRT5	-2.917304657	5.03E-15	4.77E-14
ENSG00000141622	RNF165	-2.91456907	3.02E-32	1.36E-30
ENSG00000140287	HDC	-2.912286801	1.10E-09	5.63E-09
ENSG00000171246	NPTX1	-2.912107926	2.86E-13	2.21E-12
ENSG00000139116	KIF21A	-2.910183998	8.60E-37	5.55E-35
ENSG00000164342	TLR3	-2.905980025	1.32E-29	4.84E-28

ENSG00000170962	PDGFD	-2.898183853	2.58E-39	2.05E-37
ENSG00000143184	XCL1	-2.897611312	1.34E-19	2.05E-18
ENSG00000240403	KIR3DL2	-2.887499117	5.25E-17	6.23E-16
ENSG00000156475	PPP2R2B	-2.882413105	1.53E-29	5.54E-28
ENSG00000125498	KIR2DL1	-2.867566143	2.83E-15	2.76E-14
ENSG00000158321	AUTS2	-2.863950599	1.73E-27	5.29E-26
ENSG00000183918	SH2D1A	-2.863717724	2.97E-26	8.26E-25
ENSG00000168772	CXXC4	-2.861604944	2.42E-11	1.51E-10
ENSG00000126838	PZP	-2.843403275	1.69E-15	1.70E-14
ENSG00000223638	RFPL4A	-2.817906326	0.0006134	0.00135713
ENSG00000109452	INPP4B	-2.812495615	9.03E-21	1.54E-19
ENSG00000163564	PYHIN1	-2.803901546	8.08E-30	2.99E-28
ENSG00000091181	IL5RA	-2.803886676	1.48E-19	2.25E-18
ENSG00000116106	EPHA4	-2.801624988	1.09E-33	5.44E-32
ENSG00000151623	NR3C2	-2.795816103	1.48E-20	2.48E-19
ENSG00000153563	CD8A	-2.793000689	2.05E-23	4.42E-22
ENSG00000204930	FAM221B	-2.791858441	4.10E-07	1.48E-06
ENSG00000146250	PRSS35	-2.789090006	4.78E-05	0.00012697
ENSG00000131459	GFPT2	-2.786511172	9.11E-10	4.71E-09
ENSG00000172575	RASGRP1	-2.784081095	5.98E-46	7.79E-44
ENSG00000147138	GPR174	-2.773460317	4.51E-20	7.22E-19
ENSG00000239961	LILRA4	-2.764531891	5.64E-19	8.04E-18
ENSG00000127152	BCL11B	-2.761342	7.07E-27	2.06E-25
ENSG00000145649	GZMA	-2.760034689	7.81E-16	8.11E-15
ENSG00000100385	IL2RB	-2.741657858	3.85E-27	1.14E-25
ENSG00000160654	CD3G	-2.739080699	6.76E-25	1.66E-23
ENSG00000156564	LRFN2	-2.736864534	5.32E-07	1.89E-06
ENSG00000104490	NCALD	-2.734453682	1.71E-33	8.42E-32
ENSG00000100453	GZMB	-2.72707812	1.34E-16	1.51E-15
ENSG00000173208	ABCD2	-2.721261707	2.12E-22	4.10E-21
ENSG00000158488	CD1E	-2.720042256	7.07E-18	9.11E-17
ENSG00000180739	S1PR5	-2.716065739	1.47E-13	1.17E-12
ENSG00000159708	LRRC36	-2.71418747	6.29E-10	3.32E-09
ENSG00000134539	KLRD1	-2.706300342	1.86E-22	3.63E-21
ENSG00000198756	COLGALT2	-2.699729911	6.28E-19	8.88E-18
ENSG00000204118	NAP1L6	-2.69800018	0.00095842	0.00204494
ENSG00000181847	TIGIT	-2.696691467	7.24E-20	1.13E-18
ENSG00000204983	PRSS1	-2.66657816	1.50E-06	4.99E-06
ENSG00000163508	EOMES	-2.665278934	1.36E-19	2.07E-18
ENSG00000149557	FEZ1	-2.659766501	2.66E-15	2.61E-14
ENSG00000111249	CUX2	-2.659065088	2.42E-14	2.11E-13
ENSG00000180644	PRF1	-2.654220622	5.73E-17	6.76E-16
ENSG00000198934	MAGEE1	-2.653802205	1.00E-32	4.65E-31
ENSG00000158639	PAGE5	-2.645841754	0.000118235	0.00029483

ENSG00000198846	TOX	-2.644337233	6.76E-41	6.02E-39
ENSG00000177707	NECTIN3	-2.6420766	7.09E-18	9.13E-17
ENSG00000078596	ITM2A	-2.639894693	3.45E-36	2.07E-34
ENSG00000118402	ELOVL4	-2.635137439	5.49E-13	4.10E-12
ENSG00000185920	PTCH1	-2.633448183	5.13E-32	2.29E-30
ENSG00000100450	GZMH	-2.625912191	8.45E-12	5.58E-11
ENSG00000102271	KLHL4	-2.623571697	7.68E-06	2.30E-05
ENSG00000176049	JAKMIP2	-2.621480892	1.77E-36	1.11E-34
ENSG00000102678	FGF9	-2.61431928	2.36E-11	1.48E-10
ENSG00000176083	ZNF683	-2.6111761	1.18E-06	3.99E-06
ENSG00000173406	DAB1	-2.602859306	2.91E-11	1.81E-10
ENSG00000007402	CACNA2D2	-2.601532091	2.66E-29	9.49E-28
ENSG00000198821	CD247	-2.599363325	6.95E-20	1.09E-18
ENSG00000178562	CD28	-2.589935141	3.06E-22	5.84E-21
ENSG00000169248	CXCL11	-2.582611379	3.03E-06	9.66E-06
ENSG00000148019	CEP78	-2.579535604	9.05E-43	9.42E-41
ENSG00000116667	C1orf21	-2.566316555	2.54E-24	5.97E-23
ENSG00000204116	CHIC1	-2.564982521	1.21E-52	2.45E-50
ENSG00000204583	LRCOL1	-2.561490595	1.72E-05	4.88E-05
ENSG00000188042	ARL4C	-2.557949275	5.07E-35	2.76E-33
ENSG00000138378	STAT4	-2.552751518	3.94E-39	3.12E-37
ENSG00000172296	SPTLC3	-2.54748246	4.01E-12	2.76E-11
ENSG00000117560	FASLG	-2.546139633	3.88E-15	3.74E-14
ENSG00000134954	ETS1	-2.544677671	1.50E-35	8.41E-34
ENSG00000123612	ACVR1C	-2.54301264	1.47E-10	8.42E-10
ENSG00000101004	NINL	-2.525127405	1.88E-09	9.37E-09
ENSG00000105370	LIM2	-2.52476115	9.68E-16	9.99E-15
ENSG00000152495	CAMK4	-2.518131305	6.03E-20	9.52E-19
ENSG00000147234	FRMPD3	-2.515100153	7.45E-18	9.56E-17
ENSG00000104044	OCA2	-2.514863336	2.28E-05	6.34E-05
ENSG00000081985	IL12RB2	-2.514324816	2.84E-23	5.99E-22
ENSG00000101096	NFATC2	-2.503575276	2.93E-34	1.51E-32
ENSG00000178053	MLF1	-2.503158548	3.73E-18	4.92E-17
ENSG00000133937	GSC	-2.498554875	0.001136222	0.002386227
ENSG00000196381	ZNF781	-2.496151091	2.58E-14	2.25E-13
ENSG00000154736	ADAMTS5	-2.491588903	3.61E-10	1.96E-09
ENSG00000088179	PTPN4	-2.490898386	1.21E-54	3.07E-52
ENSG00000110318	CEP126	-2.484521844	1.07E-30	4.23E-29
ENSG00000167633	KIR3DL1	-2.48445453	9.81E-09	4.42E-08
ENSG00000167618	LAIR2	-2.469996881	2.85E-09	1.39E-08
ENSG00000203756	TMEM244	-2.468204806	1.50E-05	4.30E-05
ENSG00000107679	PLEKHA1	-2.46771265	4.43E-41	4.02E-39
ENSG00000112706	IMPG1	-2.463477545	0.000672195	0.001476089
ENSG00000171817	ZNF540	-2.462683052	2.77E-20	4.53E-19

ENSG00000168824	NSG1	-2.461730631	2.01E-14	1.77E-13
ENSG00000108924	HLF	-2.461702067	3.66E-09	1.75E-08
ENSG00000027075	PRKCH	-2.458853193	8.51E-68	5.31E-65
ENSG00000205809	KLRC2	-2.457072675	1.17E-06	3.98E-06
ENSG00000203867	RBM20	-2.456191301	5.89E-07	2.07E-06
ENSG00000206077	ZDHHC11B	-2.454423256	4.79E-09	2.25E-08
ENSG00000183632	TP53TG3	-2.45241536	0.002854519	0.005554719
ENSG00000151208	DLG5	-2.446711019	1.47E-18	2.01E-17
ENSG00000270379	HEATR9	-2.445493495	3.81E-17	4.57E-16
ENSG00000146674	IGFBP3	-2.443138638	7.04E-10	3.69E-09
ENSG00000104970	KIR3DX1	-2.438271514	1.88E-08	8.14E-08
ENSG00000205336	ADGRG1	-2.438201611	6.14E-17	7.19E-16
ENSG00000183691	NOG	-2.43776585	2.95E-06	9.44E-06
ENSG00000080561	MID2	-2.436233093	1.91E-12	1.35E-11
ENSG00000016402	IL20RA	-2.432000421	0.00164991	0.003357673
ENSG00000169744	LDB2	-2.431969771	3.00E-09	1.45E-08
ENSG00000111371	SLC38A1	-2.431898269	4.06E-34	2.08E-32
ENSG00000163519	TRAT1	-2.431542459	9.00E-14	7.37E-13
ENSG00000113520	IL4	-2.428121654	2.95E-06	9.44E-06
ENSG00000213626	LBH	-2.421346252	5.89E-23	1.21E-21
ENSG00000106571	GLI3	-2.421237293	2.66E-07	9.83E-07
ENSG00000055732	MCOLN3	-2.421065265	6.19E-10	3.27E-09
ENSG00000113494	PRLR	-2.417495624	6.30E-12	4.20E-11
ENSG00000240204	SMKR1	-2.413925722	1.61E-14	1.44E-13
ENSG00000167037	SGSM1	-2.412040317	7.65E-16	7.95E-15
ENSG00000166428	PLD4	-2.406470726	8.21E-13	6.04E-12
ENSG00000135362	PRR5L	-2.405431975	2.63E-22	5.05E-21
ENSG00000154734	ADAMTS1	-2.388985681	9.71E-12	6.34E-11
ENSG00000130433	CACNG6	-2.388806599	0.001133796	0.002381465
ENSG00000174469	CNTNAP2	-2.384374613	8.22E-09	3.74E-08
ENSG00000134765	DSC1	-2.383226401	1.89E-06	6.19E-06
ENSG00000205221	VIT	-2.382967	7.43E-12	4.93E-11
ENSG00000214402	LCNL1	-2.379811507	6.84E-09	3.16E-08
ENSG00000106018	VIPR2	-2.378519708	1.35E-09	6.85E-09
ENSG00000124203	ZNF831	-2.373546096	9.64E-24	2.15E-22
ENSG00000164619	BMPER	-2.372301868	1.82E-05	5.15E-05
ENSG00000258405	ZNF578	-2.368126949	2.91E-13	2.24E-12
ENSG00000112486	CCR6	-2.366593305	8.76E-14	7.19E-13
ENSG00000184156	KCNQ3	-2.360423569	5.04E-05	0.000133378
ENSG00000131016	AKAP12	-2.35932319	1.78E-08	7.73E-08
ENSG00000165929	TC2N	-2.358253881	9.14E-23	1.85E-21
ENSG00000154027	AK5	-2.358213256	1.37E-10	7.84E-10
ENSG00000138755	CXCL9	-2.357360526	1.50E-06	4.99E-06
ENSG00000173838	10-Mar	-2.357250289	1.66E-07	6.33E-07

ENSG00000197353	LYPD2	-2.356936988	1.36E-06	4.55E-06
ENSG00000153283	CD96	-2.355010086	3.87E-25	9.74E-24
ENSG00000141448	GATA6	-2.354549213	5.38E-07	1.91E-06
ENSG00000133067	LGR6	-2.354386623	3.25E-10	1.77E-09
ENSG00000033867	SLC4A7	-2.354035393	1.45E-20	2.42E-19
ENSG00000129595	EPB41L4A	-2.351909867	1.78E-15	1.78E-14
ENSG00000130558	OLFM1	-2.349640377	7.57E-08	3.03E-07
ENSG00000185758	CLDN24	-2.349320081	0.009848861	0.017289196
ENSG00000143185	XCL2	-2.348969095	1.75E-12	1.25E-11
ENSG00000168685	IL7R	-2.348372183	3.93E-16	4.19E-15
ENSG00000273173	SNURF	-2.339438388	2.71E-09	1.32E-08
ENSG00000184731	FAM110C	-2.338934083	4.38E-10	2.35E-09
ENSG00000198780	FAM169A	-2.335672711	1.56E-14	1.39E-13
ENSG00000230561	CCDC192	-2.331728522	1.01E-06	3.47E-06
ENSG00000170500	LONRF2	-2.329394286	0.000154187	0.000378554
ENSG00000133818	RRAS2	-2.318066163	1.48E-34	7.80E-33
ENSG00000168229	PTGDR	-2.316219579	9.89E-18	1.26E-16
ENSG00000162383	SLC1A7	-2.311117732	3.33E-05	9.08E-05
ENSG00000174255	ZNF80	-2.309475126	3.06E-08	1.29E-07
ENSG00000099977	DDT	-2.308951594	0.000299532	0.000700045
ENSG00000162415	ZSWIM5	-2.308564395	9.07E-15	8.30E-14
ENSG00000145284	SCD5	-2.305442317	4.62E-25	1.15E-23
ENSG00000154645	CHODL	-2.305153935	5.48E-09	2.56E-08
ENSG00000172348	RCAN2	-2.301182566	9.40E-11	5.46E-10
ENSG00000172116	CD8B	-2.299024213	4.03E-12	2.77E-11
ENSG00000126016	AMOT	-2.296485888	2.01E-28	6.64E-27
ENSG00000240891	PLCXD2	-2.296246476	6.97E-25	1.71E-23
ENSG00000184979	USP18	-2.296098072	6.19E-16	6.48E-15
ENSG00000174738	NR1D2	-2.295786478	4.70E-25	1.17E-23
ENSG00000136111	TBC1D4	-2.290880607	2.08E-16	2.30E-15
ENSG00000197467	COL13A1	-2.282901401	1.46E-07	5.64E-07
ENSG00000196735	HLA-DQA1	-2.280485535	1.82E-06	5.98E-06
ENSG00000112394	SLC16A10	-2.276824309	6.67E-13	4.93E-12
ENSG00000152582	SPEF2	-2.276628886	3.00E-26	8.33E-25
ENSG00000170074	FAM153A	-2.275858991	1.02E-08	4.59E-08
ENSG00000113263	ITK	-2.275058182	3.08E-17	3.74E-16
ENSG00000149294	NCAM1	-2.272235674	5.09E-22	9.51E-21
ENSG00000182230	FAM153B	-2.272181715	1.45E-08	6.40E-08
ENSG00000143842	SOX13	-2.269167778	2.14E-12	1.51E-11
ENSG00000130720	FIBCD1	-2.264537998	0.000130093	0.000322519
ENSG00000114948	ADAM23	-2.262657709	4.57E-10	2.45E-09
ENSG00000115423	DNAH6	-2.255272623	2.80E-12	1.96E-11
ENSG00000115523	GNLY	-2.254971699	6.76E-09	3.12E-08
ENSG00000121753	ADGRB2	-2.251053628	7.52E-10	3.94E-09

ENSG00000171596	NMUR1	-2.239937714	2.94E-10	1.62E-09
ENSG00000213759	UGT2B11	-2.238613318	0.001141236	0.002395747
ENSG00000151612	ZNF827	-2.236588338	2.16E-17	2.67E-16
ENSG00000204475	NCR3	-2.232264135	6.87E-15	6.39E-14
ENSG00000169918	OTUD7A	-2.226430553	3.34E-13	2.56E-12
ENSG00000148488	ST8SIA6	-2.223994791	1.19E-17	1.50E-16
ENSG00000176720	BOK	-2.219927313	1.74E-07	6.61E-07
ENSG00000215475	SIAH3	-2.21970701	0.001184723	0.002476464
ENSG00000204186	ZDBF2	-2.216070051	6.28E-14	5.24E-13
ENSG00000241484	ARHGAP8	-2.215794676	0.00184825	0.003723355
ENSG00000128536	CDHR3	-2.214499235	1.96E-15	1.95E-14
ENSG00000020633	RUNX3	-2.214434813	1.91E-22	3.71E-21
ENSG00000204147	ASAH2B	-2.20861067	1.98E-12	1.40E-11
ENSG00000186197	EDARADD	-2.202771038	2.79E-08	1.18E-07
ENSG00000169245	CXCL10	-2.199196176	4.14E-07	1.49E-06
ENSG00000073861	TBX21	-2.191645762	1.47E-12	1.06E-11
ENSG00000101695	RNF125	-2.19102503	2.16E-25	5.58E-24
ENSG00000101842	VSIG1	-2.190482623	1.15E-08	5.14E-08
ENSG00000250486	FAM218A	-2.189551013	1.33E-07	5.15E-07
ENSG00000253846	PCDHGA10	-2.189268734	1.31E-06	4.41E-06
ENSG00000117600	PLPPR4	-2.179530864	0.001468053	0.003014622
ENSG00000165863	C10orf82	-2.175154096	0.00437872	0.008223625
ENSG00000101445	PPP1R16B	-2.174623037	3.07E-24	7.16E-23
ENSG00000131437	KIF3A	-2.171074648	1.27E-30	4.99E-29
ENSG00000189190	ZNF600	-2.170971458	2.10E-24	4.98E-23
ENSG00000027869	SH2D2A	-2.168778626	1.24E-12	8.98E-12
ENSG00000137440	FGFBP1	-2.168052757	0.000243271	0.00057904
ENSG00000197978	GOLGA6L9	-2.167547665	1.97E-17	2.45E-16
ENSG00000124564	SLC17A3	-2.166406765	7.50E-07	2.62E-06
ENSG00000147231	RADX	-2.163052965	1.35E-15	1.37E-14
ENSG00000166707	ZCCHC18	-2.163022031	2.70E-17	3.29E-16
ENSG00000170442	KRT86	-2.162232794	9.92E-05	0.000250588
ENSG00000167286	CD3D	-2.153932568	6.53E-10	3.44E-09
ENSG00000177932	ZNF354C	-2.152511394	2.90E-13	2.23E-12
ENSG00000196169	KIF19	-2.151046364	2.11E-09	1.05E-08
ENSG00000164114	MAP9	-2.146313104	8.60E-13	6.32E-12
ENSG00000120659	TNFSF11	-2.145669896	7.81E-07	2.71E-06
ENSG00000127334	DYRK2	-2.145219824	6.70E-43	7.02E-41
ENSG00000080166	DCT	-2.142888578	3.83E-06	1.20E-05
ENSG00000124780	KCNK17	-2.137291004	0.000126403	0.000313943
ENSG00000107018	RLN1	-2.12998663	2.32E-07	8.68E-07
ENSG00000155158	TTC39B	-2.129512814	1.90E-33	9.30E-32
ENSG00000196159	FAT4	-2.12469805	2.13E-08	9.18E-08
ENSG00000204179	PTPN20	-2.123593641	1.50E-06	4.99E-06

ENSG00000262655	SPON1	-2.120436723	1.13E-14	1.03E-13
ENSG00000242114	MTFP1	-2.117491533	9.28E-06	2.75E-05
ENSG00000149328	GLB1L2	-2.108400379	5.32E-12	3.59E-11
ENSG00000162631	NTNG1	-2.106619619	0.000238392	0.00056842
ENSG00000188636	RTL6	-2.104017759	2.18E-25	5.62E-24
ENSG00000100433	KCNK10	-2.101959959	8.62E-08	3.43E-07
ENSG00000253305	PCDHGB6	-2.098734238	7.91E-08	3.16E-07
ENSG00000070915	SLC12A3	-2.095360326	9.91E-09	4.46E-08
ENSG00000170689	HOXB9	-2.09528624	3.48E-07	1.27E-06
ENSG00000078589	P2RY10	-2.090813503	1.24E-18	1.71E-17
ENSG00000135960	EDAR	-2.087870486	2.12E-08	9.11E-08
ENSG00000197635	DPP4	-2.0871275	1.21E-11	7.79E-11
ENSG00000187118	CMC1	-2.083446158	3.19E-37	2.14E-35
ENSG00000114423	CBLB	-2.081896325	2.02E-31	8.53E-30
ENSG00000104154	SLC30A4	-2.081646677	4.96E-26	1.36E-24
ENSG00000158113	LRRC43	-2.078732791	1.10E-12	7.98E-12
ENSG00000165246	NLGN4Y	-2.077017308	0.008163769	0.014580663
ENSG00000127074	RGS13	-2.076876514	3.66E-07	1.33E-06
ENSG00000185760	KCNQ5	-2.076655315	4.21E-16	4.49E-15
ENSG00000008405	CRY1	-2.076309387	2.53E-30	9.78E-29
ENSG00000183780	SLC35F3	-2.074238186	5.73E-07	2.03E-06
ENSG00000185875	THNSL1	-2.066075688	8.03E-16	8.32E-15
ENSG00000185745	IFIT1	-2.064613672	2.79E-08	1.18E-07
ENSG00000183134	PTGDR2	-2.06419202	2.32E-09	1.14E-08
ENSG00000007264	MATK	-2.062858928	7.32E-10	3.83E-09
ENSG00000162692	VCAM1	-2.062511789	1.74E-06	5.74E-06
ENSG00000243772	KIR2DL3	-2.060572503	7.11E-09	3.27E-08
ENSG00000112782	CLIC5	-2.06026872	4.61E-18	6.06E-17
ENSG00000107742	SPOCK2	-2.059785757	2.10E-11	1.32E-10
ENSG00000176055	MBLAC2	-2.059708959	5.36E-22	1.00E-20
ENSG00000186310	NAP1L3	-2.056449487	1.20E-13	9.63E-13
ENSG00000152990	ADGRA3	-2.054370442	1.01E-13	8.23E-13
ENSG00000184349	EFNA5	-2.04945199	8.98E-10	4.65E-09
ENSG00000174370	C11orf45	-2.047096851	1.11E-06	3.79E-06
ENSG00000280575	null	-2.046215225	0.000104296	0.000262518
ENSG00000109956	B3GAT1	-2.046142716	5.21E-09	2.44E-08
ENSG00000197275	RAD54B	-2.045680097	2.10E-05	5.88E-05
ENSG00000124507	PACSIN1	-2.042278899	2.83E-08	1.20E-07
ENSG00000100027	YPEL1	-2.042227342	4.97E-31	2.03E-29
ENSG00000180447	GAS1	-2.040096955	4.31E-08	1.78E-07
ENSG00000112378	PERP	-2.038498242	8.31E-20	1.29E-18
ENSG00000090104	RGS1	-2.038455752	1.68E-09	8.40E-09
ENSG00000188322	SBK1	-2.037067957	1.14E-09	5.82E-09
ENSG00000181754	AMIGO1	-2.03477477	1.51E-11	9.65E-11

ENSG00000196911	KPNA5	-2.03451723	7.95E-36	4.67E-34
ENSG00000074966	TXK	-2.032964073	9.13E-20	1.41E-18
ENSG00000168672	FAM84B	-2.023408032	1.35E-20	2.27E-19
ENSG00000158481	CD1C	-2.02130301	7.03E-14	5.85E-13
ENSG00000165092	ALDH1A1	-2.01917527	3.02E-10	1.66E-09
ENSG00000142875	PRKACB	-2.016368814	2.21E-29	7.91E-28
ENSG00000206561	COLQ	-2.012426543	3.33E-15	3.24E-14
ENSG00000168952	STXBP6	-2.011402727	7.15E-07	2.50E-06
ENSG00000213658	LAT	-2.008012335	1.32E-13	1.06E-12
ENSG00000163687	DNASE1L3	-2.006447068	5.65E-13	4.21E-12
ENSG00000162643	WDR63	-2.00603307	5.56E-07	1.97E-06
ENSG00000198455	ZXDB	-2.003050387	1.35E-28	4.56E-27
ENSG00000128040	SPINK2	-2.002807564	1.65E-05	4.71E-05

[a] P-value computed by DESeq2.

[b] Adjusted P-value by the Benjamini and Hochberg procedure.

Supplementary Table 5. Annotation enrichment analysis of the significantly upregulated expressed genes

GOID	GOTerm	Term PValue[a]	Group PValue[b]	GOGroups	%Associated Genes	Overlap gene number
GO:0002367	cytokine production involved in immune response	0	0	Group31	19.44	28
GO:0002683	negative regulation of immune system process	0.01	0	Group31	11.48	69
GO:0002697	regulation of immune effector process	0	0	Group31	12.85	73
GO:0002718	regulation of cytokine production involved in immune response	0	0	Group31	20	24
GO:0007165	signal transduction	0	0	Group31	7.55	534
GO:0007166	cell surface receptor signaling pathway	0	0	Group31	8.65	325
GO:0019221	cytokine-mediated signaling pathway	0	0	Group31	11.31	113
GO:0030155	regulation of cell adhesion	0	0	Group31	11.12	98
GO:0034097	response to cytokine	0	0	Group31	11.05	167
GO:0045087	innate immune response	0	0	Group31	10.3	130
GO:0048583	regulation of response to stimulus	0	0	Group31	7.95	417
GO:0048584	positive regulation of response to stimulus	0	0	Group31	8.42	257
GO:0050776	regulation of immune response	0	0	Group31	9.6	138
GO:0071345	cellular response to cytokine stimulus	0	0	Group31	11.24	158
GO:0080134	regulation of response to stress	0.01	0	Group31	8.99	173
GO:0001817	regulation of cytokine production	0	0	Group30	12.28	112
GO:0001818	negative regulation of cytokine production	0	0	Group30	14.13	51
GO:0001819	positive regulation of cytokine production	0	0	Group30	12.94	77
GO:0002790	peptide secretion	0	0	Group30	10.99	89
GO:0002791	regulation of peptide secretion	0	0	Group30	11.35	74
GO:0002793	positive regulation of peptide secretion	0	0	Group30	13.09	50
GO:0009306	protein secretion	0	0	Group30	11.31	87
GO:0031347	regulation of defense response	0	0	Group30	12.17	120
GO:0031349	positive regulation of defense response	0.01	0	Group30	11.01	74

GO:0032635	interleukin-6 production	0	0	Group30	16.92	34
GO:0032637	interleukin-8 production	0	0	Group30	22.32	25
GO:0032640	tumor necrosis factor production	0	0	Group30	18.27	36
GO:0032675	regulation of interleukin-6 production	0	0	Group30	17.03	31
GO:0032677	regulation of interleukin-8 production	0	0	Group30	23.23	23
GO:0032680	regulation of tumor necrosis factor production	0	0	Group30	18.72	35
GO:0032760	positive regulation of tumor necrosis factor production	0	0	Group30	21.05	24
GO:0032940	secretion by cell	0	0	Group30	13.68	265
GO:0046903	secretion	0	0	Group30	13.29	281
GO:0050663	cytokine secretion	0	0	Group30	15.5	51
GO:0050707	regulation of cytokine secretion	0	0	Group30	14.93	43
GO:0050708	regulation of protein secretion	0	0	Group30	11.83	73
GO:0050714	positive regulation of protein secretion	0	0	Group30	14.04	50
GO:0050715	positive regulation of cytokine secretion	0	0	Group30	17.28	33
GO:0051046	regulation of secretion	0	0	Group30	10.57	108
GO:0051047	positive regulation of secretion	0	0	Group30	13.06	73
GO:0060627	regulation of vesicle-mediated transport	0	0	Group30	11.64	76
GO:0071706	tumor necrosis factor superfamily cytokine production	0	0	Group30	17.65	36
GO:1903530	regulation of secretion by cell	0	0	Group30	10.7	102
GO:1903532	positive regulation of secretion by cell	0	0	Group30	12.88	67
GO:1903555	regulation of tumor necrosis factor superfamily cytokine production	0	0	Group30	18.23	35
GO:0002685	regulation of leukocyte migration	0	0	Group29	15.56	40
GO:0002687	positive regulation of leukocyte migration	0	0	Group29	17.5	28
GO:0002688	regulation of leukocyte chemotaxis	0	0	Group29	18.42	28
GO:0006935	chemotaxis	0	0	Group29	13	107
GO:0006954	inflammatory response	0	0	Group29	15.74	150
GO:0010758	regulation of macrophage chemotaxis	0	0	Group29	32.5	13

GO:0010759	positive regulation of macrophage chemotaxis	0.01	0	Group29	47.06	8
GO:0016477	cell migration	0	0	Group29	10.46	198
GO:0030334	regulation of cell migration	0	0	Group29	11.12	121
GO:0030335	positive regulation of cell migration	0	0	Group29	11.66	75
GO:0030593	neutrophil chemotaxis	0	0	Group29	20.14	28
GO:0030595	leukocyte chemotaxis	0	0	Group29	18.3	56
GO:0032879	regulation of localization	0	0	Group29	9.7	322
GO:0040012	regulation of locomotion	0	0	Group29	10.59	132
GO:0040017	positive regulation of locomotion	0	0	Group29	11.24	79
GO:0042330	taxis	0	0	Group29	12.95	107
GO:0048246	macrophage chemotaxis	0	0	Group29	30.36	17
GO:0048870	cell motility	0	0	Group29	10.38	216
GO:0050727	regulation of inflammatory response	0	0	Group29	16.7	74
GO:0050729	positive regulation of inflammatory response	0	0	Group29	16.23	31
GO:0050900	leukocyte migration	0	0	Group29	14.22	92
GO:0051049	regulation of transport	0	0	Group29	9.48	217
GO:0051050	positive regulation of transport	0	0	Group29	9.94	124
GO:0051270	regulation of cellular component movement	0	0	Group29	10.91	136
GO:0051272	positive regulation of cellular component movement	0	0	Group29	11.32	77
GO:0060326	cell chemotaxis	0	0	Group29	15.88	67
GO:0071621	granulocyte chemotaxis	0	0	Group29	23.35	39
GO:0071622	regulation of granulocyte chemotaxis	0	0	Group29	31.48	17
GO:0097529	myeloid leukocyte migration	0	0	Group29	18.12	52
GO:0097530	granulocyte migration	0	0	Group29	22.46	42
GO:1905517	macrophage migration	0	0	Group29	25.33	19
GO:1905521	regulation of macrophage migration	0	0	Group29	26.79	15
GO:1905523	positive regulation of macrophage migration	0.01	0	Group29	40.91	9
GO:1990266	neutrophil migration	0	0	Group29	19.74	30
GO:2000145	regulation of cell motility	0	0	Group29	10.87	126
GO:2000147	positive regulation of cell motility	0	0	Group29	11.28	75
GO:0002263	cell activation involved in immune response	0	0	Group28	19.07	176
GO:0002274	myeloid leukocyte activation	0	0	Group28	20.97	181

GO:0002275	myeloid cell activation involved in immune response	0	0	Group28	21.63	157
GO:0002283	neutrophil activation involved in immune response	0	0	Group28	22.58	149
GO:0002366	leukocyte activation involved in immune response	0	0	Group28	19.23	176
GO:0002443	leukocyte mediated immunity	0	0	Group28	16.11	183
GO:0002444	myeloid leukocyte mediated immunity	0	0	Group28	21.05	156
GO:0002446	neutrophil mediated immunity	0	0	Group28	22.21	151
GO:0002682	regulation of immune system process	0	0	Group28	10.16	213
GO:0002684	positive regulation of immune system process	0	0	Group28	10.46	159
GO:0002694	regulation of leukocyte activation	0	0	Group28	10.92	82
GO:0006810	transport	0	0	Group28	8.72	528
GO:0006887	exocytosis	0	0	Group28	16.35	192
GO:0016192	vesicle-mediated transport	0	0	Group28	11.34	292
GO:0036230	granulocyte activation	0	0	Group28	22.47	153
GO:0042119	neutrophil activation	0	0	Group28	22.7	153
GO:0043299	leukocyte degranulation	0	0	Group28	21.82	156
GO:0043312	neutrophil degranulation	0	0	Group28	22.68	149
GO:0045055	regulated exocytosis	0	0	Group28	17.69	184
GO:0045321	leukocyte activation	0	0	Group28	13.53	226
GO:0050865	regulation of cell activation	0	0	Group28	11.1	89
GO:0002520	immune system development	0	0	Group27	10.04	127
GO:0007275	multicellular organism development	0.01	0	Group27	7.56	472
GO:0010646	regulation of cell communication	0	0	Group27	7.98	350
GO:0023051	regulation of signaling	0	0	Group27	8	355
GO:0030154	cell differentiation	0	0	Group27	7.88	387
GO:0048518	positive regulation of biological process	0	0	Group27	7.73	547
GO:0048522	positive regulation of cellular process	0	0	Group27	7.72	482
GO:0048731	system development	0	0	Group27	7.81	440
GO:0051239	regulation of multicellular organismal process	0	0	Group27	9.29	351
GO:0051240	positive regulation of multicellular organismal process	0	0	Group27	9.66	213

GO:0051241	negative regulation of multicellular organismal process	0	0	Group27	10.35	160
GO:2000026	regulation of multicellular organismal development	0	0	Group27	8.65	218
GO:0002237	response to molecule of bacterial origin	0	0	Group26	15.55	74
GO:0006952	defense response	0	0	Group26	11.63	251
GO:0006959	humoral immune response	0.01	0	Group26	12.26	57
GO:0009617	response to bacterium	0	0	Group26	13.22	119
GO:0010033	response to organic substance	0	0	Group26	8.84	351
GO:0019730	antimicrobial humoral response	0	0	Group26	19.3	33
GO:0032496	response to lipopolysaccharide	0	0	Group26	14.85	68
GO:0042742	defense response to bacterium	0	0	Group26	14.01	58
GO:0043207	response to external biotic stimulus	0	0	Group26	11.15	144
GO:0051707	response to other organism	0	0	Group26	11.19	144
GO:0061844	antimicrobial humoral immune response mediated by antimicrobial peptide	0	0	Group26	19.82	22
GO:0070887	cellular response to chemical stimulus	0	0	Group26	9.19	364
GO:0071216	cellular response to biotic stimulus	0	0	Group26	16.08	50
GO:0071219	cellular response to molecule of bacterial origin	0	0	Group26	17.36	50
GO:0071222	cellular response to lipopolysaccharide	0	0	Group26	16.67	46
GO:0071310	cellular response to organic substance	0	0	Group26	9.19	302
GO:0098542	defense response to other organism	0	0	Group26	11.65	81
GO:1901700	response to oxygen-containing compound	0	0	Group26	10.06	210
GO:1901701	cellular response to oxygen-containing compound	0	0	Group26	10.45	152
GO:0001525	angiogenesis	0	0	Group24	12.23	78
GO:0001568	blood vessel development	0	0	Group24	10.7	90
GO:0001944	vasculature development	0.01	0	Group24	10.49	92
GO:0045765	regulation of angiogenesis	0	0	Group24	13.87	52
GO:0045766	positive regulation of angiogenesis	0.01	0	Group24	15.14	33
GO:0048514	blood vessel morphogenesis	0	0	Group24	10.98	82

GO:0048646	anatomical structure formation involved in morphogenesis	0	0	Group24	9.81	133
GO:0072358	cardiovascular system development	0.01	0	Group24	10.37	92
GO:1901342	regulation of vasculature development	0	0	Group24	13.35	55
GO:1904018	positive regulation of vasculature development	0	0	Group24	14.94	36
GO:0006139	nucleobase-containing compound metabolic process	0	0	Group23	4.75	322
GO:0006725	cellular aromatic compound metabolic process	0	0	Group23	4.8	336
GO:0010467	gene expression	0	0	Group23	5.22	326
GO:0016070	RNA metabolic process	0	0	Group23	4.75	260
GO:0034641	cellular nitrogen compound metabolic process	0	0	Group23	4.98	375
GO:0034645	cellular macromolecule biosynthetic process	0	0	Group23	5.04	289
GO:0044271	cellular nitrogen compound biosynthetic process	0.01	0	Group23	5.19	302
GO:0046483	heterocycle metabolic process	0	0	Group23	4.82	335
GO:0090304	nucleic acid metabolic process	0	0	Group23	4.56	275
GO:1901360	organic cyclic compound metabolic process	0	0	Group23	5	361
GO:0030097	hemopoiesis	0	0	Group21	10.71	122
GO:0030099	myeloid cell differentiation	0	0	Group21	13.04	70
GO:0048513	animal organ development	0	0	Group21	8.07	342
GO:0048534	hematopoietic or lymphoid organ development	0	0	Group21	10.54	126
GO:0004175	endopeptidase activity	0	0	Group19	10.66	112
GO:0004252	serine-type endopeptidase activity	0	0	Group19	15.13	51
GO:0008233	peptidase activity	0	0	Group19	9.98	130
GO:0008236	serine-type peptidase activity	0	0	Group19	14.44	53
GO:0052547	regulation of peptidase activity	0.01	0	Group19	11.54	66
GO:0070011	peptidase activity, acting on L-amino acid peptides	0	0	Group19	9.8	123
GO:0032101	regulation of response to external stimulus	0	0	Group17	12.93	132
GO:0032102	negative regulation of response to external stimulus	0	0	Group17	13.79	59
GO:1903035	negative regulation of response to wounding	0.01	0	Group17	19.3	22
GO:0008284	positive regulation of cell population proliferation	0	0	Group16	10.18	122

GO:0042127	regulation of cell population proliferation	0	0	Group16	9.03	189
GO:0050793	regulation of developmental process	0	0	Group16	8.42	266
GO:0050764	regulation of phagocytosis	0	0	Group15	19.01	23
GO:0042116	macrophage activation	0	0	Group13	23.53	28
GO:0043030	regulation of macrophage activation	0	0	Group13	22.35	19
GO:0072593	reactive oxygen species metabolic process	0	0	Group12	15.45	55
GO:2000377	regulation of reactive oxygen species metabolic process	0	0	Group12	15.38	36
GO:2000379	positive regulation of reactive oxygen species metabolic process	0	0	Group12	19.84	25
GO:0032609	interferon-gamma production	0	0	Group11	17.61	28
GO:0032649	regulation of interferon-gamma production	0	0	Group11	18.6	24
GO:0042088	T-helper 1 type immune response	0	0	Group11	26.79	15
GO:0042592	homeostatic process	0	0	Group10	9.42	224
GO:0048878	chemical homeostasis	0	0	Group10	9.8	141
GO:0030198	extracellular matrix organization	0	0	Group09	14.78	64
GO:0043062	extracellular structure organization	0	0	Group09	14.42	75
GO:0003013	circulatory system process	0	0	Group08	11.14	79
GO:0008015	blood circulation	0	0	Group08	11.29	79
GO:0008329	signaling pattern recognition receptor activity	0	0	Group07	40	10
GO:0098543	detection of other organism	0.01	0	Group07	47.06	8
GO:0009611	response to wounding	0	0	Group06	11.48	101
GO:0042060	wound healing	0	0	Group06	11.31	81
GO:0015669	gas transport	0.01	0	Group05	37.04	10
GO:0009612	response to mechanical stimulus	0.01	0	Group04	14.13	38
GO:0006811	ion transport	0	0	Group03	9.26	191
GO:0004896	cytokine receptor activity	0.01	0	Group02	19.13	22
GO:0004222	metalloendopeptidase activity	0.01	0	Group01	17.11	26
GO:0002526	acute inflammatory response	0	0	Group00	17.16	29

[a,b] Corrected with Bonferroni step down

Supplementary Table 6. Annotation enrichment analysis of the significantly downregulated expressed genes

GOID	GOTerm	Term PValue[a]	Group PValue[b]	GOGroups	%Associated Genes	Overlap gene number
GO:0001909	leukocyte mediated cytotoxicity	0	0	Group3	10.29	14
GO:0031341	regulation of cell killing	0	0	Group3	10.81	12
GO:0002449	lymphocyte mediated immunity	0	0	Group3	5.37	24
GO:0002228	natural killer cell mediated immunity	0	0	Group3	15.38	12
GO:0002703	regulation of leukocyte mediated immunity	0	0	Group3	7	17
GO:0002705	positive regulation of leukocyte mediated immunity	0	0	Group3	8.39	13
GO:0002706	regulation of lymphocyte mediated immunity	0	0	Group3	8.29	15
GO:0002708	positive regulation of lymphocyte mediated immunity	0	0	Group3	10.74	13
GO:0031343	positive regulation of cell killing	0.01	0	Group3	11.84	9
GO:0001910	regulation of leukocyte mediated cytotoxicity	0.01	0	Group3	10.31	10
GO:0006955	immune response	0	0	Group2	3.08	87
GO:0002682	regulation of immune system process	0	0	Group2	3.39	71
GO:0046649	lymphocyte activation	0	0	Group2	4.16	40
GO:0050776	regulation of immune response	0	0	Group2	3.69	53
GO:0042110	T cell activation	0	0	Group2	5.57	35
GO:0030098	lymphocyte differentiation	0	0	Group2	5.86	26
GO:0030217	T cell differentiation	0	0	Group2	7.36	22
GO:0046631	alpha-beta T cell activation	0.01	0	Group2	7.74	13
GO:0048583	regulation of response to stimulus	0	0	Group1	2.59	136
GO:0006968	cellular defense response	0	0	Group0	17.19	11

[a,b] Corrected with Bonferroni step down
